# Supplementary material for: Exposure, hazard, and vulnerability all contribute to Schistosoma haematobium re-infection in northern Senegal
Source: PLoS Negl Trop Dis. 2021 Oct 5;15(10):e0009806. doi: 10.1371/journal.pntd.0009806 (PMC8525765; doi:10.1371/journal.pntd.0009806)
Supplement: S1 Checklist — (DOC) [file pntd.0009806.s001.doc]

Table S6. STROBE checklist for cross-sectional studies

|  | Item No | Recommendation | In manuscript |
| --- | --- | --- | --- |
| **Title and abstract** | 1 | (*a*) Indicate the study’s design with a commonly used term in the title or the abstract | Title page, title  Abstract |
|  |  | (*b*) Provide in the abstract an informative and balanced summary of what was done and what was found | Title page, abstract |
| Introduction | | |  |
| Background/rationale | 2 | Explain the scientific background and rationale for the investigation being reported | Introduction, paragraphs 1-5 |
| Objectives | 3 | State specific objectives, including any prespecified hypotheses | Introduction, paragraph 6 |
| Methods | | |  |
| Study design | 4 | Present key elements of study design early in the paper | Methods, paragraphs 1-6 |
| Setting | 5 | Describe the setting, locations, and relevant dates, including periods of recruitment, exposure, follow-up, and data collection | Methods: Study area and population (paragraphs 1-2),  Data Collection (paragraphs 3-6) |
| Participants | 6 | (*a*) Give the eligibility criteria, and the sources and methods of selection of participants | Methods: Study area and population (paragraph 2)  Data collection (paragraphs 3-6) |
| Variables | 7 | Clearly define all outcomes, exposures, predictors, potential confounders, and effect modifiers. Give diagnostic criteria, if applicable | Methods: Data analysis,  Infection metrics (paragraph 7)  Exposure indices (paragraph 8-11)  Hazard indices (paragraph 12)  Vulnerability indices (paragraph 13)  Statistical analysis (paragraph 14) |
| Data sources/ measurement | 8* | For each variable of interest, give sources of data and details of methods of assessment (measurement). Describe comparability of assessment methods if there is more than one group | Methods: Data collection (paragraphs 3-6),  Data analysis: Infection metrics (paragraph 7), Exposure indices (paragraph 8-11), Hazard indices (paragraph 12), Vulnerability indices (paragraph 13-14) |
| Bias | 9 | Describe any efforts to address potential sources of bias | Methods: Statistical analysis (paragraph 14) |
| Study size | 10 | Explain how the study size was arrived at | Methods: Study area and population (paragraph 2)  Data collection: Household survey data collection (paragraph 4)  Figure S1 |
| Quantitative variables | 11 | Explain how quantitative variables were handled in the analyses. If applicable, describe which groupings were chosen and why | Methods: Data analysis, Infection metrics (paragraph 7), Exposure indices (paragraph 8-11), Hazard indices (paragraph 12), Vulnerability indices (paragraph 13-14) |
| Statistical methods | 12 | (*a*) Describe all statistical methods, including those used to control for confounding | Methods: Statistical analysis (paragraphs 15-17) |
|  |  | (*b*) Describe any methods used to examine subgroups and interactions | Methods: Statistical analysis (paragraph 15) |
|  |  | (*c*) Explain how missing data were addressed | Figure S1 |
|  |  | (*d*) If applicable, describe analytical methods taking account of sampling strategy | Methods, paragraph 15 |
|  |  | (*e*) Describe any sensitivity analyses | Methods, paragraph 17 |
| Results | | |  |
| Participants | 13* | (a) Report numbers of individuals at each stage of study—eg numbers potentially eligible, examined for eligibility, confirmed eligible, included in the study, completing follow-up, and analysed | Methods: Study area and population (paragraph 1), Parasitology data collection (paragraph 3), Household survey data collection (paragraph 4), Figure S1 |
|  |  | (b) Give reasons for non-participation at each stage | Methods: Study area and population (paragraph 1) |
|  |  | (c) Consider use of a flow diagram | Figure S1 |
| Descriptive data | 14* | (a) Give characteristics of study participants (eg demographic, clinical, social) and information on exposures and potential confounders | Results: Characteristics of study population (paragraph 1-5) |
|  |  | (b) Indicate number of participants with missing data for each variable of interest | Figure S1 |
| Outcome data | 15* | Report numbers of outcome events or summary measures | Results: Characteristics of study population (paragraph 2),  Table 2 |
| Main results | 16 | (*a*) Give unadjusted estimates and, if applicable, confounder-adjusted estimates and their precision (eg, 95% confidence interval). Make clear which confounders were adjusted for and why they were included | Results: Model-averaged estimates (paragraph 12-17),  Figure 2 |
|  |  | (*b*) Report category boundaries when continuous variables were categorized | Methods: Data analysis, Infection metrics (paragraph 7), Exposure indices (paragraph 8-11), Hazard indices (paragraph 12), Vulnerability indices (paragraph 13-14) |
|  |  | (*c*) If relevant, consider translating estimates of relative risk into absolute risk for a meaningful time period | Not applicable |
| Other analyses | 17 | Report other analyses done—eg analyses of subgroups and interactions, and sensitivity analyses | Not applicable |
| Discussion | | |  |
| Key results | 18 | Summarise key results with reference to study objectives | Results: Relative importance of exposure, hazard and vulnerability (paragraphs 9-11), Table 6  Discussion: paragraph 1 |
| Limitations | 19 | Discuss limitations of the study, taking into account sources of potential bias or imprecision. Discuss both direction and magnitude of any potential bias | Discussion: lines 132-137, 145-161, 171-173, 202-206, 229-246 |
| Interpretation | 20 | Give a cautious overall interpretation of results considering objectives, limitations, multiplicity of analyses, results from similar studies, and other relevant evidence | Discussion: Conclusion, lines 255-264 |
| Generalisability | 21 | Discuss the generalisability (external validity) of the study results | Discussion: Conclusion, lines 265-272 |
| Other information | | |  |
| Funding | 22 | Give the source of funding and the role of the funders for the present study and, if applicable, for the original study on which the present article is based | Funding, lines 289-296 |

*Give information separately for exposed and unexposed groups.

**Note:** An Explanation and Elaboration article discusses each checklist item and gives methodological background and published examples of transparent reporting. The STROBE checklist is best used in conjunction with this article (freely available on the Web sites of PLoS Medicine at http://www.plosmedicine.org/, Annals of Internal Medicine at http://www.annals.org/, and Epidemiology at http://www.epidem.com/). Information on the STROBE Initiative is available at www.strobe-statement.org.
