# Supplementary figures and images for: Exposure, hazard, and vulnerability all contribute to Schistosoma haematobium re-infection in northern Senegal
Source: PLoS Negl Trop Dis. 2021 Oct 5;15(10):e0009806. doi: 10.1371/journal.pntd.0009806 (PMC8525765; doi:10.1371/journal.pntd.0009806)

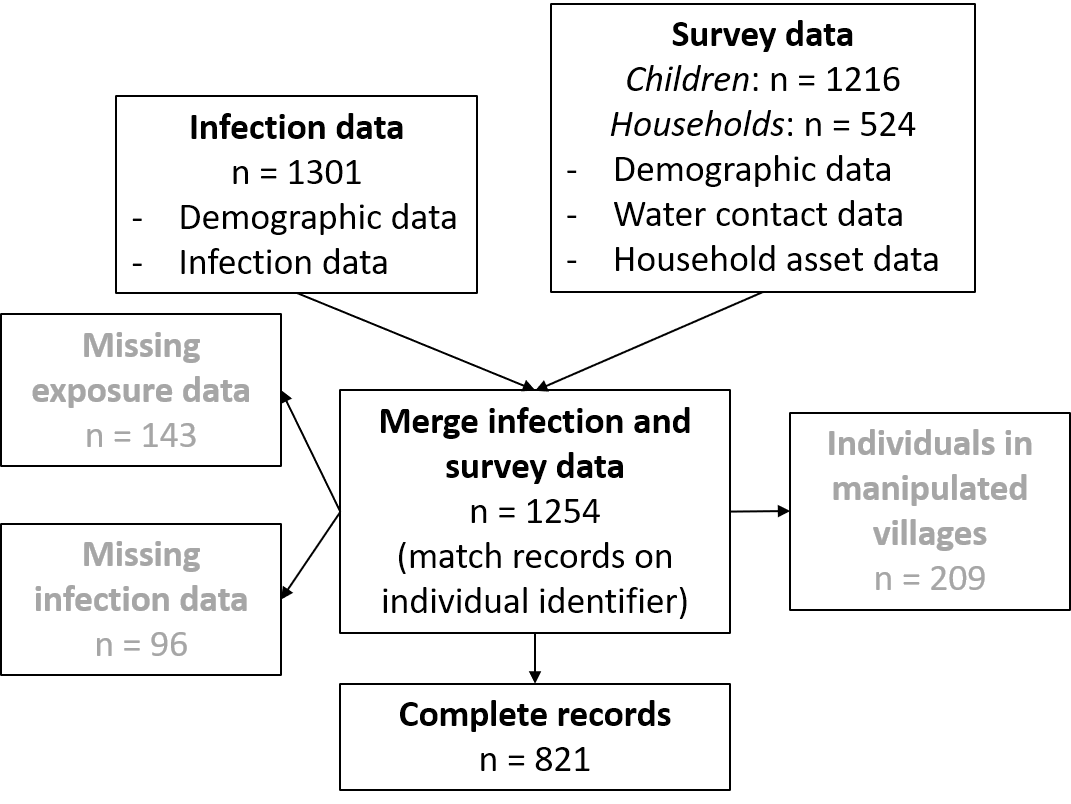

Supplement: S1 Fig — Sample sizes resulting from the merging and cleaning of human data from parasitological and household surveys and removing data from manipulated sites. (TIF) [file pntd.0009806.s002.tif]

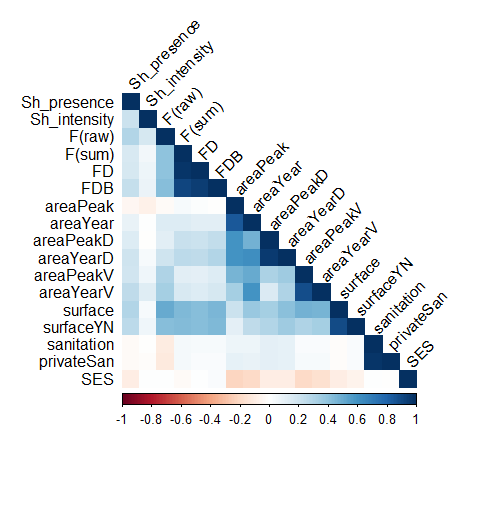

Supplement: S2 Fig — Correlations between all variables used in all models. (TIFF) [file pntd.0009806.s003.tiff]
