# Supplementary material for: Exposure, hazard, and vulnerability all contribute to Schistosoma haematobium re-infection in northern Senegal
Source: PLoS Negl Trop Dis. 2021 Oct 5;15(10):e0009806. doi: 10.1371/journal.pntd.0009806 (PMC8525765; doi:10.1371/journal.pntd.0009806)
Supplement: S1 Table — Household survey items used in in this analysis, including the module to which an item belonged, the level at which it was measured as well as the wording of and response categories provided for each question. (PDF) [file pntd.0009806.s004.pdf]

S1 Table. Household survey items used in in this analysis, including the module to which an item belonged, the level at which it was measured as well as the wording of and response categories provided for each question

Module	Level	Question	Response Categories
Demographic characteristics	Individual	What is ____'s sex?	Male / Female
	Individual	What is ____'s age?	[years]
	Individual	Is ____ present to respond? _	Yes / No
	Individual	How many times has ____ visited a water access point during the last seven days?	[number of visits]
Water contact	Individual	Did ____ do laundry in surface water (e.g. the river, the lake or agricultural fields or canals) in the last two weeks?	Yes / No
	Individual	Did ____ do dishes in surface water (e.g. the river, the lake or agricultural fields or canals) in the last two weeks?	Yes / No
	Individual	Did ____ collect water from surface water (e.g. the river, the lake or agricultural fields or canals) in the last two weeks?	Yes / No
	Individual	Did ____ irrigate crops using surface water (e.g. the river, the lake or agricultural fields or canals) in the last two weeks?	Yes / No
	Individual	Did ____ water or wash livestock in surface water (e.g. the river, the lake or agricultural fields or canals) in the last two weeks?	Yes / No
	Individual	Did ____ fish in surface water (e.g. the river, the lake or agricultural fields or canals) in the last two weeks?	Yes / No
	Household	With what frequency, on average, do members of the household do laundry?	Not performed / Less than once a week / Once a week / Multiple times per week / Once a day / Multiple times per day
	Household	With what frequency, on average, do members of the household do dishes?	Not performed / Less than once a week / Once a week / Multiple times per week / Once a day / Multiple times per day
	Household	With what frequency, on average, do members of the household collect water?	Not performed / Less than once a week / Once a week / Multiple times per week / Once a day / Multiple times per day

S1 Table. Household survey items used in in this analysis, including the module to which an item belonged, the level at which it was measured as well as the wording of and response categories provided for each question

	Household	With what frequency, on average, do members of the household irrigate crops?	Not performed / Less than once a week / Once a week / Multiple times per week / Once a day / Multiple times per day
	Household	With what frequency, on average, do members of the household water or wash livestock?	Not performed / Less than once a week / Once a week / Multiple times per week / Once a day / Multiple times per day
	Household	With what frequency, on average, do members of the household fish?	Not performed / Less than once a week / Once a week / Multiple times per week / Once a day / Multiple times per day
	Household	With what frequency, on average, do members of the household bathe?	Not performed / Less than once a week / Once a week / Multiple times per week / Once a day / Multiple times per day
Living conditions	Household	Is the household electrified?	Yes / No
	Household	How many of [asset] does the household have? (assets include: radio, television, washing machine, gas stove, cooling fan, mobile phone, bicycle, cart, canoe, irrigation pump)	[number owned]
	Household	How many rooms are in the household?	[number]
	Household	What is the principal drinking water source in the household?	Piped water / Borehole / Well / Surface water / Other
	Household	What is the principal water source used for washing clothes?	Piped water / Borehole / Well / Surface water / Other
	Household	What type of toilet is primarily used by members of the household?	Flush toilet / Latrine / No toilet / Other
	Household	What is the primary floor material?	Sand / Cement / Tile
	Household	What is the primary roofing material?	Straw / Wood / Zinc / Cement / Shingles
	Household	What is the primary material of the exterior walls?	Cement / Wood / Straw / Other
