# Supplementary material for: Exposure, hazard, and vulnerability all contribute to Schistosoma haematobium re-infection in northern Senegal
Source: PLoS Negl Trop Dis. 2021 Oct 5;15(10):e0009806. doi: 10.1371/journal.pntd.0009806 (PMC8525765; doi:10.1371/journal.pntd.0009806)
Supplement: S2 Table — Activity-specific values used to estimate duration and extent of water contact. Duration estimates were drawn from published research that took place in a similar setting and extent was estimated from body surface area interviews. (PDF) [file pntd.0009806.s005.pdf]

S2 Table. Duration and extent of water contact. Activity-specific values used to estimate duration and extent of water contact. Duration estimates were drawn from published research that took place in a similar setting and extent was estimated from body surface area interviews.

**Activity**	**Duration**[a]	**Percent body surface area**	
	**minutes**	**mean**	**sd**
Laundry	13.3[b]	35.8	16.3
Dishes	13.3[b]	31.8	12.1
Water collection	2.0	45.3	15.9
Irrigation	7.9	22.5	10.0
Livestock	9.3	41.5	14.9
Shore fishing	23.9	47.6	17.5
Bathing	11.5	100[d]	ND

ND = no data; [a] Activity-specific duration of contact derived from Sow et al 2011; [b] Categories classified as ‘household’ and divided by two; [c] Classified as ‘disembarking’, [d] initial responses in interviews indicating bathing, by definition, involved water contact for entire body prompted us to exclude it from subsequent interviews
