# Supplementary material for: Exposure, hazard, and vulnerability all contribute to Schistosoma haematobium re-infection in northern Senegal
Source: PLoS Negl Trop Dis. 2021 Oct 5;15(10):e0009806. doi: 10.1371/journal.pntd.0009806 (PMC8525765; doi:10.1371/journal.pntd.0009806)
Supplement: S3 Table — Frequencies (n (row %)) of household surface water use by quintiles of asset-based index of socio-economic status (SES). (PDF) [file pntd.0009806.s006.pdf]

S3 Table. Household surface water use by socio-economic status. Frequencies (n (row %)) of household surface water use by quintiles of asset-based index of socio-economic status (SES).

	SES quintile					
	**1st (low)**	**2nd**	**3rd**	**4th**	**5th (high)**	**Total**
Neither drinking nor laundry	54 (13.5)	56 (14.0)	70 (17.5)	112 (28.0)	108 (27.0)	400
Either drinking or laundry	45 (17.0)	36 (13.6)	38 (14.3)	67 (25.3)	79 (29.8)	265
Both drinking and laundry	34 (21.8)	24 (15.4)	42 (26.9)	22 (14.1)	34 (21.8)	156
Total	133	116	150	201	221	821
