# Supplementary material for: Exposure, hazard, and vulnerability all contribute to Schistosoma haematobium re-infection in northern Senegal
Source: PLoS Negl Trop Dis. 2021 Oct 5;15(10):e0009806. doi: 10.1371/journal.pntd.0009806 (PMC8525765; doi:10.1371/journal.pntd.0009806)
Supplement: Attachment — Submitted filename: ms.pdf [file pntd.0009806.s009.pdf]

PLOS Neglected Tropical Diseases

Exposure, hazard and vulnerability all contribute to Schistosoma haematobium re-infection in northern Senegal

--Manuscript Draft--

Manuscript Number:	PNTD-D-20-02100
Full Title:	Exposure, hazard and vulnerability all contribute to Schistosoma haematobium re-infection in northern Senegal
Short Title:	Exposure, hazard and vulnerability in S. haematobium re-infection in Senegal
Article Type:	Research Article
Keywords:	Schistosoma haematobium; Senegal; exposure; hazard; vulnerability; infectious disease risk; socio-ecological systems; multi-model inference
Abstract:	Background<br><br>The risk of infectious diseases, including snail-borne schistosomiasis, is determined by three interrelated components: exposure, hazard and vulnerability. For schistosomiasis, exposure occurs through behaviors involving water contact, but not without the environmental hazard of snails and parasites in the water. Socio-economic vulnerability makes it difficult to reduce exposure in the presence of hazard, because it increases reliance on hazardous activities and environments. We aimed to quantify the contributions of exposure, hazard and vulnerability to schistosome re-infection presence and intensity.<br><br>Methodology/Principal Findings<br><br>We used cross-sectional parasitological data from 821 school-aged children in 13 villages along the Senegal River, survey data from 411 households where those children resided and ecological data from all 24 village water contact sites. We fit mixed-effects logistic and negative binomial regressions with indices of exposure, hazard and vulnerability as explanatory variables of Schistosoma haematobium infection, along with demographic control variables. Multi-model inference was used to determine the relative importance of each component of risk and model averaging was used to quantify associations between infection outcomes and indices of hazard, exposure and vulnerability. The most important component of S. haematobium presence was hazard (Σw_i = 0.95), followed by vulnerability (Σw_i = 0.91), while the most important component of S. haematobium intensity was exposure (Σw_i = 1.00), followed by hazard (Σw_i = 0.76). Hazard was positively associated with infection presence (OR = 1.49, 95% CI 1.12, 1.97), while exposure was positively associated with infection intensity.<br><br>Conclusions/Significance<br><br>Our findings highlight how social (exposure and vulnerability) and environmental (hazard) processes act together to facilitate the acquisition and accumulation of schistosome infection from the environment across time and space. This approach can inform targeting of social and environmental interventions as complements to mass drug administration.
Additional Information:	
**Question**	**Response**
**Financial Disclosure**<br><br>Enter a financial disclosure statement that describes the sources of funding for the work included in this submission. Review the submission guidelines for detailed requirements. View published research	The authors acknowledge funding from the National Science Foundation Couple Natural Human Systems program (grant no 1414102) and the Bill and Melinda Gates Foundation (OPP1114050). AJL was supported by a James and Nance Kelso Fellowship through the Stanford Interdisciplinary Graduate Fellowship (SIGF) program at Stanford University. CLW was supported by the Michigan Society of Fellows at the University of Michigan, a Sloan Research Fellowship from the Alfred P. Sloan Foundation, a UW Innovation Award and a grant from the National Science Foundation

Powered by Editorial Manager® and ProduXion Manager® from Aries Systems Corporation

articles from *PLOS NTDs* for specific examples.<br><br>This statement is required for submission and **will appear in the published article** if the submission is accepted. Please make sure it is accurate.<br><br>**Unfunded studies**<br><br>Enter: *The author(s) received no specific funding for this work.*<br><br>**Funded studies**<br><br>Enter a statement with the following details:<br>• Initials of the authors who received each award<br>• Grant numbers awarded to each author<br>• The full name of each funder<br>• URL of each funder website<br>• Did the sponsors or funders play any role in the study design, data collection and analysis, decision to publish, or preparation of the manuscript?<br>• **NO** - Include this sentence at the end of your statement: *The funders had no role in study design, data collection and analysis, decision to publish, or preparation of the manuscript.*<br>• **YES** - Specify the role(s) played.<br><br>* typeset	(OCE-1829509). The funders had no role in study design, data collection, interpretation or the decision to submit the work for publication.
**Competing Interests**<br><br>Use the instructions below to enter a competing interest statement for this submission. On behalf of all authors, disclose any competing interests that could be perceived to bias this work—acknowledging all financial support and any other relevant financial or non-financial competing interests.	The authors have declared that no competing interests exist.

This statement **will appear in the published article** if the submission is accepted. Please make sure it is accurate. View published research articles from *PLOS NTDs* for specific examples.<br><br>**NO authors have competing interests**<br><br>Enter: *The authors have declared that no competing interests exist.*<br><br>**Authors with competing interests**<br><br>Enter competing interest details beginning with this statement:<br><br>*I have read the journal's policy and the authors of this manuscript have the following competing interests: [insert competing interests here]*<br><br>* typeset	
**Data Availability**<br><br>Authors are required to make all data underlying the findings described fully available, without restriction, and from the time of publication. PLOS allows rare exceptions to address legal and ethical concerns. See the PLOS Data Policy and FAQ for detailed information.<br><br>A Data Availability Statement describing where the data can be found is required at submission. Your answers to this question	No - some restrictions will apply

constitute the Data Availability Statement and **will be published in the article**, if accepted.<br><br>**Important:** Stating 'data available on request from the author' is not sufficient. If your data are only available upon request, select 'No' for the first question and explain your exceptional situation in the text box.<br><br>Do the authors confirm that all data underlying the findings described in their manuscript are fully available without restriction?	
**Describe where the data may be found in full sentences. If you are copying our sample text, replace any instances of XXX with the appropriate details.**<br><br>• If the data are **held or will be held in a public repository**, include URLs, accession numbers or DOIs. If this information will only be available after acceptance, indicate this by ticking the box below. For example: *All XXX files are available from the XXX database (accession number(s) XXX, XXX.*).<br>• If the data are all contained **within the manuscript and/or Supporting Information files**, enter the following: *All relevant data are within the manuscript and its Supporting Information files.*<br>• If neither of these applies but you are able to provide **details of access elsewhere**, with or without limitations, please do so. For example:<br><br>*Data cannot be shared publicly because of [XXX]. Data are available from the XXX Institutional Data Access / Ethics Committee (contact via XXX) for*	Data aggregated to the village level can be found in the following Dryad repository: https://doi.org/10.5061/dryad.s7h44j15p<br>Individual- and household-level data needed to replicate the analysis while safeguarding participant anonymity can be made available by the corresponding author upon reasonable request.

researchers who meet the criteria for access to confidential data.<br><br>*The data underlying the results presented in the study are available from (include the name of the third party and contact information or URL).*<br>• This text is appropriate if the data are owned by a third party and authors do not have permission to share the data.<br><br>* typeset	
Additional data availability information:	Tick here if the URLs/accession numbers/DOIs will be available only after acceptance of the manuscript for publication so that we can ensure their inclusion before publication.

Powered by Editorial Manager® and ProduXion Manager® from Aries Systems Corporation

Manuscript
Click here to access/download;Manuscript;Lund_etal_EHV_PNTDs_initial.doc

Exposure, hazard and vulnerability all contribute to *Schistosoma haematobium* re-infection in northern Senegal

Andrea J. Lund[1*], Susanne H. Sokolow[2,3], Isabel J. Jones[2], Chelsea L. Wood[4], Sofia Ali[5], Andrew Chamberlin[2], Alioune Badara Sy[6], M. Moustapha Sam[6], Nicolas Jouanard[6,7], Anne-Marie Schacht[6,8], Simon Senghor[6], Assane Fall[6], Raphael Ndione[6], Gilles Riveau[6,8], Giulio A. De Leo[2,3], David López-Carr[9]

[1] Emmett Interdisciplinary Program in Environment and Resources, Stanford University, Stanford, California, United States of America

[2] Hopkins Marine Station, Stanford University, Pacific Grove, California, United States of America

[3] Woods Institute for the Environment, Stanford University, Stanford, California, United States of America

[4] School of Aquatic and Fishery Sciences, University of Washington, Seattle, Washington, United States of America

[5] Stanford University, Stanford, California, United States of America

[6] Centre de Recherche Biomédicale– Espoir Pour La Sante, Saint Louis, Sénégal

[7] Station d'Innovation Aquacole, Saint Louis, Sénégal

[8] Center for Infection and Immunology of Lille, Institut Pasteur de Lille, Lille, France

[9] Department of Geography, University of California, Santa Barbara, CA, USA

* Corresponding author

Email: andrea.janelle.lund@gmail.com (AJL)

Abstract

Background: The risk of infectious diseases, including snail-borne schistosomiasis, is determined by three interrelated components: exposure, hazard and vulnerability. For schistosomiasis, exposure occurs through behaviors involving water contact, but not without the environmental hazard of snails and parasites in the water. Socio-economic vulnerability makes it difficult to reduce exposure in the presence of hazard, because it increases reliance on hazardous activities and environments. We aimed to quantify the contributions of exposure, hazard and vulnerability to schistosome re-infection presence and intensity.

Methodology/Principal Findings: We used cross-sectional parasitological data from 821 school-aged children in 13 villages along the Senegal River, survey data from 411 households where those children resided and ecological data from all 24 village water contact sites. We fit mixed-effects logistic and negative binomial regressions with indices of exposure, hazard and vulnerability as explanatory variables of *Schistosoma haematobium* infection, along with demographic control variables. Multi-model inference was used to determine the relative importance of each component of risk and model averaging was used to quantify associations between infection outcomes and indices of hazard, exposure and vulnerability. The most important component of *S. haematobium* presence was hazard ($\Sigma w_i = 0.95$), followed by vulnerability ($\Sigma w_i = 0.91$), while the most important component of *S. haematobium* intensity was exposure ($\Sigma w_i = 1.00$), followed by hazard ($\Sigma w_i = 0.76$). Hazard was positively associated with infection presence (OR = 1.49, 95% CI 1.12, 1.97), while exposure was positively associated with infection intensity.

Conclusions/Significance: Our findings highlight how social (exposure and vulnerability) and environmental (hazard) processes act together to facilitate the acquisition and accumulation of

schistosome infection from the environment across time and space. This approach can inform targeting of social and environmental interventions as complements to mass drug administration.

Author Summary

While the impacts of natural hazards tend to be described in terms of social determinants such as exposure and vulnerability, the risk for infectious disease is often expressed in terms of environmental determinants without fully considering the socio-ecological processes that put people in contact with infective agents of disease. In the case of schistosomiasis, risk is determined by human interactions with freshwater environments where schistosome parasites circulate through populations of aquatic snails. In this study, we quantified the relative exposure, hazard and vulnerability to schistosome re-infection of schoolchildren in an endemic region of northern Senegal. We find that hazard and vulnerability influence whether a child becomes infected, while exposure and hazard influence the burden of worms once infection is acquired, where increasing numbers of worms lead to increasing severity of disease. Our findings underscore the importance of evaluating social and environmental determinants of disease simultaneously; omitting measures of exposure, hazard or vulnerability may limit our understanding of risk.

Introduction

When a natural hazard like a flood or hurricane is imminent, risk managers and first responders must account for the exposure and vulnerability of affected populations, conditions and processes that are dynamic in both space and time [1]. Precious resources will be misplaced if authorities provide assistance to families whose homes have not flooded, because those resources will have been diverted away from families who have actually been exposed to the impacts of the hazard. Similarly, vulnerable families with a reduced capacity to anticipate, respond or recover from the impacts of a natural hazard will require more assistance than less vulnerable families [1]. An effective response to natural disasters, as a result, requires evaluation of where a natural hazard overlaps with exposure and how vulnerability mediates exposure and its impacts in time and space.

In contrast to the risk of natural disasters, the risk of infectious disease is often defined in terms of its environmental determinants. Risk of insect-borne diseases like Lyme disease and malaria are often described in terms of 'entomological risk,' such as the density of infected nymphal ticks (for Lyme disease) [2] or the probability of an infective mosquito bite (for malaria) [3]. Similarly, wildlife biodiversity – representing the size of the natural reservoir of potentially zoonotic pathogens – is used as a proxy for spillover risk [4]. While these measures reflect important components of pathogen transmission, their focus on the source of the disease-causing agent overlooks the socio-ecological processes that connect those infectious agents with susceptible people, thereby facilitating transmission and determining risk. Such environmental metrics represent a single component of infectious disease risk: hazard [3,5]. Meanwhile, exposure and vulnerability reflect the roles of human behavior and social structures in transforming hazard into risk. Exposure describes contact between a person and a pathogen (e.g.,

exposure to a hazard), while vulnerability determines the extent to which people can anticipate, adapt to and potentially mitigate the impacts of infection [3,6].

These three components of risk – exposure, hazard, and vulnerability – operate in concert rather than isolation. They comprise the breadth of socio-ecological conditions that put and keep people in contact with infectious agents of disease. Few studies address all three components simultaneously, but those that do show how human interaction with the environment transforms a hazard into risk for infection and disease. For example, accounting for both entomological risk (i.e. hazard) and exposure explained Lyme disease incidence in New York when entomological risk alone did not [2]. Incorporating social vulnerability and entomological risk into a spatially-explicit risk assessment of malaria in East Africa showed how different interventions could be targeted to different places with different needs [7,8]. Across disease systems, insights about the relative contributions of exposure, hazard and vulnerability can inform which interventions to employ to mitigate risk and where, when and how to implement them.

The risk for schistosomiasis – a disease caused by parasitic worms from the genus *Schistosoma* – can also be divided into these three distinct components. Schistosomes complete a two-host transmission cycle between humans and specific species of freshwater snail. People become infected through contact with freshwater where schistosomes' free-living infective larval stage (called cercariae) emerge from infected snails and burrow into human skin. Since cercariae are difficult to detect, schistosome hazard is often described as 'malacological risk,' and quantified using ecological indicators of snail populations within a water source. Similarly, exposure to schistosomes is often approximated by studying human water contact behavior because the process of cercariae burrowing into skin upon contact is difficult to directly observe. Even so, exposure to surface water on its own does not imply risk for infection; the presence of

infected snails in a water source, and the circulation of parasites through the snail population is what renders water contact harmful [3]. Lastly, vulnerability determines whether or not a person can avoid or mitigate the schistosome hazard in their surroundings, either by reducing their exposure or seeking treatment for infection. The relative disadvantage of certain groups may make risk mitigation difficult, obliging people to remain in contact with parasite-laden water even if they are aware of the risk [3].

Approximately 200 million people are infected with schistosomes worldwide, making schistosomiasis second only to malaria in the global burden of parasitic disease [9,10]. Schistosome infections are treatable with the antiparasitic drug praziquantel. Since the 1980s, mass drug administration (MDA) campaigns employing praziquantel have been the central strategy of schistosomiasis control, displacing the snail control interventions of the preceding decades [11,12]. MDA has played an important role in reducing morbidity due to schistosomiasis, but because praziquantel does not prevent new infections, the benefits of MDA have not been realized universally across all contexts [13]. The identification of persistent hot spots – or settings whose prevalence and intensity of infection remains high despite repeated rounds of treatment – has underscored the need for interventions that control transmission and complement the morbidity control achieved by MDA [14,15]. This approach will require both ecological and social points of intervention, whose identification and implementation can be informed by understanding the relative contributions of hazard, exposure and vulnerability in the risk for acquiring and accumulating schistosome infections.

In this study, we used parasitological, ecological and socio-economic data collected from 16 villages in the lower basin of the Senegal River to better understand the relative contribution of exposure, hazard and vulnerability to the burden of urogenital schistosomiasis in an endemic

region of northern Senegal. In this setting, the construction of a dam and irrigation infrastructure has reduced the region's susceptibility to drought and famine, while simultaneously facilitating schistosome transmission. Prior to dam construction, the transmission of *S. haematobium* occurred at low levels on a seasonal basis, but the disease has since become hyperendemic with perennially high prevalence and intensity of infection [16–18]. Dam construction also coincided with the introduction and rapid spread of *S. mansoni* and intestinal schistosomiasis, which remains co-endemic with *S. haematobium* [19,20]. Similarly, agricultural livelihoods have shifted from traditional flood recession and rainfed cultivation to more intensively irrigated crops [21]. The social and environmental changes in this setting have collectively affected exposure, hazard and vulnerability to schistosome infection. We compared the relative contributions of exposure, hazard and vulnerability as explanatory variables of both presence and intensity of *S. haematobium* infection and quantified the magnitude and precision of the association between infection outcomes and indices of each component of risk. By assessing the relative contributions of exposure, hazard and vulnerability to schistosomiasis risk, we identify the circumstances under which ecological, infrastructural and behavioral interventions can complement mass drug administration in campaigns to control and eliminate schistosomiasis.

Methods

Study area and population

This study used cross-sectional data collected in 2017 and 2018 as part of a longitudinal study of schistosome infection in school-aged children, including parasitological examinations of urine samples screening for *S. haematobium* infections, survey data from the residents and heads of households where those children reside, and ecological data from water contact sites in their corresponding villages. Sixteen villages along the lower Senegal River, its tributaries, and the

Lac de Guiers in northwest Senegal were selected; these villages were representative of the rural, high-transmission sites common in the region (see appendix of [18] for details on village selection; Fig. 1). Briefly, the criteria used to select villages included proximity to freshwater, presence of water access sites, presence of a school with sufficient enrollment in target primary school grades, a non-zero prevalence of self-reported infection, and accessibility in the rainy season [18]. Of these 16 study villages, six were located along the Senegal River and its tributaries while ten were located on the shores of Lac de Guiers. Three villages (one on the river and two on the lake) were excluded from this analysis because a vegetation removal intervention took place in these sites in 2017, potentially influencing hazard indices (Figure 1)

Figure 1. The lower basin of the Senegal River, where parasitological, socio-economic and ecological data were collected from 821 school-aged children in 411 households and 24 water access points in 13 villages.

In each village, school-aged children were recruited from grades 1–3 in village schools, and each provided urine samples for determining *S. haematobium* infection presence and intensity in January–March 2018. In July 2018, we attempted to reach all the households where students enrolled in the school-based parasitology study lived, in order to administer a questionnaire about household demographics and water contact behavior. This study received approval from the National Committee of Ethics for Health Research from the Republic of Senegal (Protocol #SEN14/33) as well as the Institutional Review Boards of the University of California, Santa Barbara (Protocol #19-17-0676) and Stanford University (Protocol #43130). Children were enrolled in the parasitology study after providing assent alongside written informed consent from the child's parent or guardian. All survey respondents provided verbal informed consent.

Data collection

Parasitology data collection. Through schools in all 16 villages, a total of 1,480 school-aged children were enrolled at baseline in February–April 2016. Of those, 1,301 remained enrolled during the third year of follow-up in January–March 2018 and successfully produced urine samples on the testing days. At each time point, urine samples were analyzed by urine filtration for *S. haematobium* infection [22]. Following sample collection, all children were treated at school with two rounds of praziquantel administration at 40 mg/kg. Thus, infection data collected in January–March 2018, which were used as outcome variables in this study, reflect re-infection following treatment over the course of the preceding year [23–25].

Household survey data collection. Household-level survey data were collected at the beginning of the rainy season in July 2018, following parasitological data collection for that year. In all 16 villages, we aimed to reach all households with children enrolled in the school-based parasitology study. Of the 1,301 students enrolled in the parasitology study, we reached the households (n = 524) of 1,216 students (93.5%; Fig. S1). The household survey instrument included six main modules (S1 Table). The modules used in this analysis included: (1) individual-level demographic information, (2) individual-level, two-week recall of contact with surface water for six primary domestic and occupational chores requiring contact with water, (3) average frequency of each water contact activity reported at the household level, (4) socio-economic indicators, such as reported access to improved water and sanitation infrastructure and (5) geographic coordinates of household locations. The collection of water contact data in the survey was designed to elicit information on water contact behavior over the course of a typical week.

Body surface area interview data collection. In all but one village, brief interviews were conducted with village residents, often at village water points in August 2016. In each interview,

respondents were asked how much of the body typically comes into contact with water for each of six common water activities: (1) washing laundry, (2) washing dishes, (3) collecting water, (4) irrigating fields, (5) washing or watering livestock and (6) fishing from shore. Responses for each activity were registered on a diagram used to measure burn size [26]. Two interviews were conducted in each village – one with an adult man and one with an adult woman – for a total of 30 interviews and 210 activity-specific observations.

Ecological data collection. Snail surveys were performed at 32 water access sites in all 16 study villages, the details of which are described elsewhere [18]. Briefly, randomized snail sampling was stratified by three microhabitat types (emergent vegetation, non-emergent vegetation and open water/mud bottom), such that sampling effort within a site was proportionate to the relative area of each microhabitat. Imagery from Google Earth and an unmanned aerial vehicle (drone) was used to identify site boundaries and designate fifteen random sampling points within each microhabitat. At each point, exhaustive sampling for snails occurred within a quadrat (76.2 cm x 48.26 cm x 48.26 cm; area = 0.3677 square meters). The snails collected from each quadrat were place into labeled vials and returned to the laboratory for identification and infection screening via shedding and dissection assays. Molecular identification of schistosome cercariae shed from captured snails in the laboratory distinguished human-infecting schistosome species from species that affect non-human animals.

Data analysis

Infection metrics. Schistosome infections were determined to be present when any of the samples collected from an individual student contained at least one schistosome egg. Infection intensity quantified egg counts (per 10 mL urine) by taking the median across the samples

collected at both visits. Taking the median of the value(s) kept our estimates conservative, because they were not skewed by missing or zero values.

Exposure indices. Exposure indices were calculated using data from three sources: (1) individual- and household-level survey data, (2) body surface area interviews and (3) a previous study of water contact behavior in a similar setting [27]. These indices aggregated reported water contact across the six water contact activities described above, including in the body surface area interview form and the water contact module in the household survey. Whether or not a person performed a given activity in the preceding two weeks was recorded as binary individual-level variables in the household survey. The average frequency of each activity was reported at the household level in the survey, while duration and extent of contact were drawn from literature-based estimates and body surface area interviews, respectively (Table S2). Exposure indices based on these activity-specific data incorporated varying degrees of complexity, including weekly frequency-only estimates, weekly time exposed and weekly time exposed adjusted for mean body surface area exposure.

Two indices measured frequency of contact with water. One index [*F (raw)*] was a categorical variable derived directly from a single survey question, where each child was asked to recall the number of visits made to a water access point in the preceding seven days. The second [*F (sum)*] was a numeric variable derived from activity-specific measures collected through the survey at the individual and household levels. Individual-level water contact data indicated whether or not each member of the household had performed each activity in surface water (e.g., lake, river, irrigation canals) in the preceding two weeks. Dichotomous responses were registered for all members of the household. Response categories for frequency of each water contact activity were collected at the household level (e.g., one value per household) and

converted to numeric values representing times per week (e.g., a reported frequency of once per day was converted to a numeric value of 7 times per week). The numeric values of household-level activity frequency were multiplied by reports of individual-level activity to generate estimated weekly contacts by activity for all children in the data set:

$$F(sum) = \sum_{activity}(activity\ performed) * (frequency) \qquad (1)$$

Total time exposed per week (*FD*) was calculated by multiplying the weekly frequency of an activity by the estimated duration of that activity, as calculated from direct observations in another study in a similar setting [27]. The product of frequency and duration was then summed across all activities for each child to generate an estimated total time (in minutes) of contact with water over the course of a week.

$$FD = \sum_{activity}(activity\ performed) * (frequency) * (duration) \qquad (2)$$

Total time exposed per week was then adjusted by estimated body surface area in contact with water for a given activity (*FDB*). We used data from body surface area interviews (Table S2) to estimate how much of the body came into contact with water for each activity. For each observation, the percent of body surface area (%BSA) was summed for each body segment reported as exposed for that activity using published values for burn size estimation [26]. Mean %BSA for each activity was calculated across all interview respondents. Activity-specific estimates of body surface area exposed (in square meters) were calculated by multiplying mean %BSA by an estimated total body surface area for children under 14 years of age [28]. Estimated body surface area exposure required for each activity was multiplied by the total time exposed for that activity, giving a BSA-adjusted time per week.

$$FDB = \sum_{activity}(activity\ performed) * (frequency) * (duration) * (body\ surface) \ (3)$$

Hazard indices. Hazard indices were calculated from ecological field data collected at the 32 water access points in the same 16 villages where parasitology and household survey data were collected. A previous study in this setting found that area of suitable snail habitat (e.g., area covered by non-emergent aquatic vegetation) was the best environmental predictor of presence and intensity of *S. haematobium* infection [18]. We developed six hazard indices based on area of snail habitat, which varied based on (1) season, (2) spatial relationship and (3) level of aggregation. Hazard variables either captured the area of suitable snail habitat (1) for the peak transmission season preceding parasitological data collection for each water access point (July – August 2017; peak) or (2) summed across the three ecological data collection periods in the year preceding parasitological data collection (May–June 2017, July–August 2017 and January 2018; year). Summing habitat area across the year represents an integrated hazard metric across the three dominant climatic seasons in the region. We primarily used data from the water access site nearest to each household, based on straight-line distance, but also captured the spatial relationship between households and the nearest water access sites by scaling *peak* and *year* hazard indices by the distance between a child's household and the water access site. Lastly, area of suitable snail habitat during the transmission peak and the entire year were summed across all water points in a village to generate village-level indices.

Vulnerability indices. Vulnerability indices were calculated from the household survey data and focused on access (or lack thereof) to water and sanitation infrastructure at the household level (S1 Table). Two survey questions concerned water sources: (1) what is the principal source water used by members of the household for drinking? and (2) what is the principal source of water used for doing laundry? Responses to these two questions were used to calculate the extent to which each household depended on surface water for household needs (e.g., surface water is

used for neither drinking water nor laundry; surface water is used for either drinking water or laundry; or, surface water is used for both drinking water and laundry). This variable was also dichotomized (e.g., a household used surface water for any activity versus no activities). Two additional survey questions concerned sanitation infrastructure: (1) what type of toilet is primarily used by members of this household? and (2) is this toilet shared with other households? Responses to these two questions were used to calculate a three-level variable describing sanitation access (e.g., private toilet, shared toilet, no toilet). A dichotomous version of this variable was also calculated (e.g., private toilet versus shared or no toilet). This categorization was chosen because few households reported having no toilet.

A final index of vulnerability was an index of socio-economic status (SES) derived from data on durable assets and household conditions reported in the household survey [29,30]. Principal components analysis was performed using the *prcomp()* function in the *stats* package (version 3.5.1) in R [31]. Loadings for individual variables in the first principal component were used to calculate a numeric socio-economic score for each household. Numeric scores were then divided into quintiles.

Statistical analysis. We used mixed-effects logistic and negative binomial regression models to assess exposure, hazard and vulnerability indices as explanatory variables of schistosome infection presence (i.e., infected versus uninfected) and intensity (i.e., number of eggs detected per 10mL urine), respectively. In all models, we controlled for key social and demographic covariates: age and sex of the child, village location (i.e., river versus lake) and population size. We also included random intercepts (households nested within villages) to account for the hierarchy in the data. Mixed effects logistic regression was performed using the *lme4* package

(version 1.1-21) [32], while mixed effects negative binomial regression was performed using the *glmmTMB* package (version 1.0.1) [33] in R (version 3.5.1) [31].

We used information-criterion (IC)-based model selection and multi-model inference to determine the relative importance of each component of risk [34]. For each outcome, we first fit a set of exposure-only, hazard-only and vulnerability-only models. The Akaike Information Criterion (AIC) was used to discern the best-fitting index (or indices) of each component of risk. We retained all variables whose ΔAIC was less than 2 across the models in a given group [34]. This subset of variables was then used to fit models that included various combinations of the three components of risk: (1) exposure and hazard (E-H), (2) exposure and vulnerability (E-V), (3) hazard and vulnerability (H-V) and (4) all three: exposure, hazard and vulnerability (E-H-V). Because fewer indices of exposure, hazard and vulnerability were used in these combination models of infection presence (based on AIC for exposure-, hazard-, and vulnerability- only models), we fit a set of 28 candidate models for *S. haematobium* presence and 47 candidate models for *S. haematobium* re-infection intensity. Regression diagnostics indicated no problem with multicollinearity.

For both sets of models, we calculated AIC, ΔAIC and Akaike weights (w_i) [34]. The sum of Akaike weights across the exposure, hazard and vulnerability components of risk was used to quantitatively assess their relative importance across the full set of candidate models for both presence and intensity outcomes [34]. Additionally, for each index of exposure, hazard and vulnerability, we calculated model-averaged estimates of the magnitude and precision of the association with both infection outcomes using the *AICcmodavg* package in R [35]. Odds ratios (OR) and rate ratios (RR) with 95% confidence intervals (95% CIs) were estimated for presence and intensity outcomes, respectively.

Results

Characteristics of the study population

Of the 821 school-aged children with complete information across the parasitological, survey and ecological data sets, the mean age was 9.3 years and approximately half (414/821; 50.4%) were male (Table 1). On average, these children lived within 521 meters of a water access point on the Senegal River, a tributary, or the Lac de Guiers, which contained between zero and 149,775 square meters of suitable snail habitat in the peak transmission season. Approximately half of the households where these children lived relied on surface water collected from water access points for at least one household need, and the majority of households (82.6%) reported primarily using private sanitation infrastructure (Table 1).

Table 1. Demographic characteristics of the overall study population, stratified by sex

Variable	Overall	Males	Females
Observations [n]	821	414	407
Age (years) [mean (SD)]	9.3 (3.1)	9.2 (2.2)	9.3 (3.8)
Distance to water point (m) [mean (SD)]	521.6 (505.3)	522.2 (530.6)	521.0 (475.5)
Water point habitat area (m^2) [mean (range)]	38,857 (0 - 149775)	38,898 (0 – 100,361)	38,815 (0 – 149,775)
Surface water dependence [n (%)]			
Neither drinking nor laundry	400 (48.7)	211 (51.0)	189 (46.4)
Either drinking or laundry	265 (32.3)	129 (31.2)	136 (33.4)
Both drinking and laundry	156 (19.0)	74 (17.9)	82 (20.1)
Sanitation facility [n (%)]			
Private toilet	679 (82.7)	342 (82.6)	337 (82.8)
Shared toilet	134 (16.3)	68 (16.4)	66 (16.2)
No toilet	8 (0.9)	4 (0.9)	4 (1.0)
Asset-based wealth index [n (%)]			
1st quintile (Lowest)	133 (16.2)	76 (18.4)	57 (14.5)
2nd quintile	116 (14.1)	57 (13.7)	59 (14.5)
3rd quintile	150 (18.3)	82 (19.8)	68 (16.7)
4th quintile	201 (24.5)	100 (24.2)	101 (24.8)
5th quintile (highest)	221 (26.9)	99 (23.9)	122 (30.0)

The burden of *S. haematobium* among this study population was high. The overall prevalence was 66.7% and was slightly higher among boys (72.9%) than among girls (60.4%)

(Table 2). The geometric mean of *S. haematobium* infection intensity was 4.9 eggs per 10 mL urine (SD = 117.4) overall, 6.4 eggs per 10 mL urine (SD = 66.8) for boys and 3.7 eggs per 10 mL urine (SD = 152.0) for girls (Table 2).

Table 2. Presence and intensity of *S. haematobium* infection in study population

Variable	Overall	Males	Females
Observations [n]	821	414	407
Infection presence [infected n (%)]	548 (66.7)	302 (72.9)	246 (60.4)
Infection intensity [GM (SD)]	4.9 (117.4)	6.4 (66.8)	3.7 (152.0)

GM = geometric mean eggs/10mL urine

When asked about contact with surface water generally, over one-third (39.1%) of school-aged children reported no visits to village water access sites in the preceding seven days while approximately one-quarter (26.7%) visited once or twice a week and the remainder (34.3%) reported more frequent visits (Table 3). When prompted to recall activity-specific contact with surface water (e.g., doing laundry or washing livestock), the average sum of total weekly contacts across all activities was much higher (mean = 8.5 times per week, SD = 10.9). The weekly frequency of some of these activities (e.g., bathing and dish washing) exceeded that reported from the single survey question prompting seven-day recall of all visits to the water access point (Table 3).

Table 3. Exposure indices calculated from individual-level responses to water contact questions in the household survey, overall and stratified by sex

Variable	Overall	Males	Females
Categorical frequency [n (%)]			
No visits per week	321 (39.1)	145 (33.0)	176 (43.2)
1-2 visits per week	219 (26.7)	121 (29.2)	98 (24.1)
3-6 visits per week	68 (8.3)	33 (8.0)	35 (8.6)
7 visits per week	173 (21.1)	95 (22.9)	78 (19.2)
7+ visits per week	40 (4.9)	20 (4.8)	20 (4.9)
Weekly activity-specific frequency [mean (SD)]			
Bathing	4.7 (5.2)	4.6 (5.1)	4.8 (5.3)
Water collection	1.4 (3.4)	1.0 (2.9)	1.8 (3.9)
Dishes	1.2 (3.3)	0.1 (1.0)	2.2 (4.3)
Laundry	0.7 (1.9)	0.1 (0.7)	1.3 (2.5)

Livestock	0.3 (1.5)	0.5 (1.9)	0.1 (0.9)
Irrigation	0.2 (1.2)	0.2 (1.2)	0.1 (1.3)
Fishing	0.1 (0.8)	0.1 (0.8)	0.03 (0.7)
Numeric frequency [mean (SD)]	8.5 (10.9)	6.6 (7.5)	10.4 (13.2)
Total weekly time exposed [mean (SD)]	87.3 (111.4)	66.0 (74.6)	109.0 (135.9)
BSA-adjusted weekly time exposed [mean (SD)]	74.5 (83.4)	65.6 (71.7)	83.5 (93.0)

Bathing was assumed to involve contact with the entire body surface, while shore fishing (47.6%) and water collection (45.3%) involved the next greatest body surface area in contact with water (S2 Table). Irrigation (22.5%) was the activity with the least body surface area exposed (S2 Table). Using the reported duration of activity-specific water contact, we estimated that the school-aged children in this setting spend, on average, 87.3 minutes (SD = 111.4) in contact with surface water per week, an estimate that is higher for girls (mean = 109.0 minutes, SD = 135.9) than boys (mean = 66.0 minutes, SD = 74.6). Adjusting weekly total duration of contact by total body surface exposed during a specific activity reduced this overall estimate slightly to 74.5 m^2-minutes, with similar variation between boys (mean = 65.6 m^2-minutes, SD = 71.7) and girls (mean = 83.5 m^2-minutes, SD = 93.0).

While survey data indicate that dependence on surface water is not evenly distributed across the quintiles of the asset-based SES index (Kruskal-Wallis test, $X^2 = 14.0$, df = 4, P-value = 0.007), we find that those who do depend on surface water for at least one household task are relatively evenly distributed across SES quintiles (S3 Table). For example, almost a third of the households (29.8%) that use surface water for either drinking or laundry are in the highest SES quintile. We find a similar pattern for dependence on surface water for both drinking and laundry: almost a quarter of households (21.8%) in the highest SES quintile rely on surface water for both chores, which is identical to the proportion of households in the lowest SES quintile with the same reliance on surface water (S3 Table).

Best-fitting indices of exposure, hazard and vulnerability

Four exposure indices, six hazard indices and five vulnerability indices were considered in a set of mixed-effects logistic regression models of *S. haematobium* infection presence (n = 28) and a set of mixed-effects negative binomial regression models of *S. haematobium* infection intensity (n = 47; Table 4). The full comparison of model fit, including the Akaike weight (w_i) for each individual model, is summarized in S4 Table for all presence models and in S5 Table for all intensity models. Table 4 summarizes each variable used to approximate exposure, hazard or vulnerability as well as the number of models that variable appeared in for each outcome. For infection presence, all four exposure-only models were within a ΔAIC of 2. For infection intensity, the categorical frequency of water point visits (*F (raw)*) outperformed all three numeric indices of exposure (numeric frequency [*F (sum)*], total time exposed [*FD*] and BSA-adjusted time exposed [FDB]*)*, as it had the lowest AIC of the four exposure-only models, the ΔAIC for the next best-fitting model of infection intensity far exceeded 2 (ΔAIC = 16.52; S5 Table). As a result, we retained all four indices of exposure for models of infection presence, each of which appeared in a total of four of the 28 (14.2%) presence models. The categorical frequency of water point visits was the only index of exposure retained in subsequent models of infection intensity, appearing in a total of 20 of 47 (43.5%) models in that set (Table 4).

Table 4. Summary of the indices of exposure, hazard and vulnerability that were compared in a set of 28 mixed effects logistic regression models of *S. haematobium* infection presence and a set of 47 mixed effects negative binomial regression models of *S. haematobium* infection intensity and the number (%) of models each variable appeared in

	Variable	Description	Models (n (%)) Presence	Intensity
Exposure	F (raw)	Number of visits to water access point in previous week, from single survey question	4 (14.2)	20 (43.5)
	F (sum)	Sum of activity-specific weekly contacts with water, based on individual-level indicators of activity and average household-level frequencies from household survey	4 (14.2)	1 (2.2)
	FD	Weekly time exposed derived from activity-specific survey data, as in *F (sum)*, combined with literature-derived data on duration of exposure	4 (14.2)	1 (2.2)
	FDB	Weekly time exposed adjusted for activity-specific body surface area exposed, data estimated from body surface area interviews	4 (14.2)	1 (2.2)
Hazard	areaPeak	Area of snail habitat at the water access site nearest to household during peak season	1 (3.6)	8 (17.4)
	areaYear	Area of snail habitat at the water access site nearest to household summed over year	1 (3.6)	8 (17.4)
	areaPeak_d	Area of snail habitat at the water access site nearest to household during the peak season, adjusted by the distance between the site and the household	1 (3.6)	8 (17.4)
	areaYear_d	Area of snail habitat at the water access site nearest to child's household summed over year, adjusted by the distance between the site and the household	10 (35.7)	8 (17.4)
	areaPeakV	Area of snail habitat summed across all water points in a village during the peak season	1 (3.6)	1 (2.2)
	areaYearV	Area of snail habitat summed across all water points in a village and across year	1 (3.6)	1 (2.2)
Vulnerability	surface	Dependence on surface water for household needs derived from survey data: (0) neither laundry or drinking, (1) either laundry or drinking or (2) both laundry and drinking	10 (35.7)	1 (2.2)
	surfaceYN	Dichotomous version of surface water dependence for any versus no activities	1 (3.6)	10 (21.7)
	sanitation	Primary sanitation infrastructure used by members of a household: (0) none, (2) shared toilet, (3) private toilet	1 (3.6)	10 (21.7)
	privateSan	Dichotomous version of sanitation, where members of a household use either a private toilet or shared/no toilet	1 (14.2)	10 (21.7)
	assetIndex	Quintiles of an asset-based SES index created using principal components analysis	1 (3.6)	1 (2.2)

A single index of hazard – the area of snail habitat at the water access site nearest to a child's household summed over the three climatic seasons of ecological data collection, adjusted by the distance between the site and the household (*areaYear_d)* – had a ΔAIC ≤ 2 compared to other hazard-only models of *S. haematobium* presence (S4 Table). As a result, *areaYear_d* was the only index of hazard to appear in more than one model of *S. haematobium* presence (Table 4). In contrast, five indices of hazard had a ΔAIC ≤ 2 compared to other hazard-only models of *S. haematobium* intensity (S5 Table). These variables, which included the area of snail habitat at the water access site nearest to a child's household during the peak transmission season (*areaPeak*) as well as area summed over the entire year (*areaYear*). The distance-adjusted analogs of these variables (*areaPeak_d* and *areaYear_d*) were also retained as was the area of snail habitat summed across all water points in a village and across the full year (*areaYearV*). These five indices of hazard were retained in subsequent models that combined hazard with other components of risk, such that all five appear in multiple models of *S. haematobium* intensity (Table 4).

One index of vulnerability – categorical dependence on surface water (*surface*) – best fit the *S. haematobium* presence data (e.g., ΔAIC ≤ 2 among vulnerability-only models; S4 Table). This variable was retained for subsequent models that combined components of risk and

appeared in ten (35.7%) of the 28 presence models (Table 4). In contrast, binary dependence on surface water (*surfaceYN*) as well as categorical and binary variables for sanitation infrastructure (*sanitation* and *privateSan*, respectively) all had ΔAIC ≤ 2 among vulnerability-only models of *S.haematobium* intensity (S4 Table). Each appeared in ten (21.7%) of the 47 intensity models (Table 4).

Relative importance of exposure, hazard and vulnerability

Across these sets of candidate models, no single model emerged as the best-fitting model of *S. haematobium* re-infection presence or intensity (S4 and S5 Tables). The largest w_i for mixed effects logistic regression models of *S. haematobium* presence was 0.28. A total of five models were classified as having substantial weight (ΔAIC < 2) [34]. Four of these five models included all three components of risk while the fifth contained indices of hazard and vulnerability (Table 5)

Table 5. Inclusion of the three components of risk in models of *S. haematobium* presence and intensity models with substantial support (ΔAIC <2)

No.	E	H	V	AIC	ΔAIC	w_i	Σ w_i
Presence models (from S4 Table)							
24		X	X	837.37	0	0.28	0.28
26	X	X	X	838.32	0.96	0.18	0.46
27	X	X	X	838.57	1.21	0.16	0.62

28	X	X	X	838.89	1.52	0.13	0.75
25	X	X	X	839.21	1.84	0.11	0.86
Intensity models (from S5 Table)							
1	X			5494.29	0	0.08	0.08
16	X	X		5494.41	0.12	0.08	0.15
19	X	X		5494.46	0.17	0.08	0.22
42	X	X	X	5494.46	0.17	0.07	0.30
22	X		X	5494.49	0.20	0.07	0.37
18	X	X		5494.86	0.57	0.06	0.42
43	X	X	X	5494.86	0.57	0.06	0.48
40	X	X	X	5494.95	0.66	0.06	0.54
36	X	X	X	5494.98	0.68	0.06	0.59
21	X		X	5495.21	0.92	0.05	0.64
17	X	X		5495.30	1.01	0.05	0.69
20	X	X		5495.50	1.21	0.04	0.73
41	X	X	X	5495.70	1.41	0.04	0.77
38	X	X	X	5495.81	1.52	0.04	0.81
37	X	X	X	5495.95	1.66	0.03	0.84
39	X	X	X	5496.16	1.87	0.03	0.87
46	X	X	X	5496.19	1.90	0.03	0.90
23	X		X	5496.23	1.94	0.03	0.93

Components of risk indicated by E (exposure), H (hazard) and V (vulnerability). X indicates whether a variable from a given component of risk was included in the model.

The largest w_i for mixed-effects negative binomial regression models of *S. haematobium* intensity was 0.08, with 17 models classified as having substantial weight [34]. Just over half (9 /17) of these models included indices of all three components of risk. An index of exposure was

included in all of the best models of re-infection intensity, including an exposure-only model which had the lowest AIC of all models of re-infection intensity (Table 5).

We used the sum of Akaike weights (Σw_i) across the entire set of candidate models for each outcome to quantify the relative importance of each component of risk (Table 6). We found that hazard was the most important component of risk for presence of disease in school children ($\Sigma w_i = 0.95$). The relative importance of hazard for *S. haematobium* presence was followed by vulnerability ($\Sigma w_i = 0.91$) and then exposure ($\Sigma w_i = 0.66$; Table 6). The relative importance of each component of risk differed for models of *S. haematobium* re-infection intensity, where exposure (measured as categorical frequency of contact with surface water) was the most important metric ($\Sigma w_i = 1.00$), followed by hazard ($\Sigma w_i = 0.76$) and then vulnerability ($\Sigma w_i = 0.66$).

Table 6. Sum of Akaike weights (Σw_i) as a measure of relative importance of the three components of risk for *S. haematobium* infection presence and intensity

	Σw_i		
	Exposure	Hazard	Vulnerability
Infection presence	0.66	0.95	0.91
Infection intensity	1.00	0.76	0.66

Model-averaged estimates

We used model averaging to calculate the magnitude and precision of the association between both infection outcomes and all indices of exposure, hazard and vulnerability that appeared in more than one model across the full set of candidate models and the demographic control variables used in each model (Fig. 2). For the infection presence outcome, we found that the odds of infection increased with distance-adjusted area of snail habitat at the nearest water point to a child's household summed over the year preceding infection data collection. The association corresponds with a 45% increase in the odds of infection with each square meter of snail habitat over the course of an entire year, with a confidence interval contained entirely above the null value of 1 (*areaYear_d*; OR = 1.49, 95% CI 1.12, 1.97).

Figure 2. Model-averaged point estimates and 95% confidence intervals for association between *S. haematobium* infection presence (odds ratios; top) and intensity (rate ratios; bottom) with each index of exposure (black triangles), hazard (turquoise circles), vulnerability (blue squares) as well as demographic characteristics (orange diamonds).

Associations between infection presence and indices of vulnerability were less precise, with confidence intervals that included the null value of 1 (Fig. 2). When comparing children whose households relied on surface water for multiple household tasks to those who relied on surface water for no household tasks, we found an almost nine-fold increase in the odds of infection with a confidence interval entirely above the null value of 1 (*Categorical surface water use* [2 activities vs 0]; OR = 8.67, 95% CI 1.67, 45.60). A similar association comparing

households’ use of surface water for a single activity was also positive, but its confidence interval crossed the null (*Categorical surface water use* [1 activity vs 0]; OR = 1.34, 95% CI 0.84, 2.14).

All levels of the exposure index suggest a positive association with infection presence (categorical frequency [*F (raw)*]), but only one level (*Categorical frequency* [3-6 times/week vs none]) had a confidence interval that did not include the null value of 1 (OR = 2.18, 95% CI 1.03, 4.66). Remaining levels of categorical exposure (1-2 times/week vs none, 7 times/week vs none and >7 times/week versus none) cannot be considered to be statistically different from the null (Fig. 2). All three numeric indices of exposure had point estimates slightly below 1 (e.g., 0.9) but all of their confidence intervals included in the null value (Fig. 2).

Two of the four demographic control variables had significant associations with infection presence (Fig. 2). Children living in lake villages were five times more likely to be infected compared to children living in river villages (*Village location*; OR = 5.37, 95% CI 1.14, 25.28). The odds of infection were 54% lower among girls compared to boys (*Child sex*; OR = 0.46, 95% CI 0.33, 0.63) and younger children were less likely to be infected than older children

(*Child age*; OR = 0.78, 95% CI 0.63, 0.96). Village population size was not associated with infection presence (*Village population*; OR = 1.16, 95% CI 0.64, 2.10).

For the infection intensity outcome, we found that the number of eggs in a 10 mL urine sample increased for all levels of exposure (*Categorical frequency* [*F (raw)*]; Fig. 2). These point estimates ranged from a 2.59- to 3.86-fold increase in egg burden among children reporting any exposure compared to those reporting no exposure. Confidence intervals for all estimates were entirely above the null value of 1 (95% CIs: [*1-2x/week vs none*] 1.52, 4.44, [*3-6x/week vs none*] 1.88, 8.00, [*7x/week vs none*] 1.55, 5.70, [*>7x/week vs none* 1.22, 7.77; Fig. 2). The directionality of the associations between infection intensity and indices of vulnerability are similar to those for infection presence (e.g., above 1 for surface water use, below 1 for sanitation infrastructure), but with point estimates closer to the null and wider confidence intervals that include the null value of 1 (Fig. 2).

The measures of association between infection intensity and hazard are mixed: distance-scaled and village-level indices have point estimates greater than 1 while raw values of habitat area (i.e., not scaled by distance) for the nearest water point to a child's home are less than 1. The confidence intervals for all associations between *S. haematobium* infection intensity and

hazard indices include the null value of 1. Associations between infection intensity and demographic control variables were similar to the associations described for these variables with presence outcomes, with the exception that the children living in lake villages had significantly larger worm burdens compared to those living in river villages (Fig. 2).

Discussion

We found that components of risk describing exposure, hazard and vulnerability all had some importance in explaining the occurrence of *S. haematobium* in school-aged children in this endemic setting. Based on the sums of Akaike weights calculated across both sets of models, however, hazard and vulnerability were the most important components of *S. haematobium* infection presence, while exposure and hazard were the most important components of *S. haematobium* infection intensity. These differences highlight the distinct factors that influence whether someone becomes infected versus whether they accumulate a large worm burden, with implications for the design of schistosomiasis control and elimination programs and guidelines.

The importance of hazard and vulnerability to the infection presence outcome reflects the conditions that influence whether or not a person acquires an infection or not. Any exposure may lead to infection, making additional exposure irrelevant to this binary outcome once a person

becomes infected. But hazard in the environment, or the number of infected snails emitting cercariae from a certain amount of available habitat, is more likely to increase the risk of infection so long as some exposure is happening. Vulnerability through the household activities that involve regular water contact, like dependence on surface water, for example, ensures some baseline level of exposure.

Similarly, the importance of exposure and hazard to infection intensity reflects the conditions that influence the accumulation of worms. The intensity of infection is theoretically proportional to exposure, with each new adult worm the result of a separate infection event [36]. While this has not always borne out empirically [27,37], our data agree with the theoretical expectation that more exposure should lead to higher intensity infection. Such accumulation of worms can lead to severe morbidity. A study in Uganda found the prevalence of liver fibrosis to be five times greater in people who had lived in the *S. mansoni*-endemic area since birth compared to those with just a 10-year residency, leading the authors to conclude that the duration of exposure over time is an important risk factor for developing the severe morbidity resulting from prolonged high-intensity infections [38]. Another study in China found that water contact itself was not associated with infection intensity but, similar to our findings, accounting for both water contact and a measure of cercarial risk did [39].

While the recognition that schistosomiasis risk is determined by the convergence of social (exposure and vulnerability) and environmental factors (hazard) is not new, few studies comprehensively address all three components of risk for schistosomiasis specifically or for infectious diseases generally. Our results allow us to think more concretely about how to choose interventions and thereby target schistosomiasis control. Reducing exposure through behavior change interventions that minimize contact with water is likely to contribute more to morbidity control (e.g., reductions in infection intensity) than transmission control (e.g., reductions in infection presence). Reducing vulnerability through the provision and use of piped water infrastructure will give people an alternative to surface water, allowing them to avoid risky exposures and thus, lower infection intensity. Reducing hazard in the environment through snail control interventions will be essential to reducing transmission and reducing the likelihood of both acquiring and accumulating infections from the environment. Understanding the interplay between different components of risk in a specific setting can facilitate the design of effective and efficient context-specific control strategies.

In this study, we use data sets collected by three distinct teams under the same project in the same study villages: (1) collection, processing and diagnosis of *S. haematobium* infection from urine samples collected from school-aged children on a yearly basis with associated

treatment with praziquantel, (2) exhaustive sampling of snail populations and their habitats in the water access points in all villages, and (3) the socio-economic surveys in all the households where school-aged children in the parasitology study lived. All three data streams, combined with data on the spatial relationship between water access points and surveyed households, were important for explaining patterns of infection in children. Our results underscore the importance of interdisciplinary and mixed-methods research when addressing complex socio-environmental problems like the transmission of schistosomiasis and other infectious diseases. Even so, this study examines the convergence of these processes for just one of the two schistosome species that circulate and cause disease in this setting. The particularities of *S. mansoni* transmission (i.e., snail intermediate hosts with distinct habitat associations and different human behaviors associated with contamination of the environment) may not be governed by the same relationships with exposure, hazard and vulnerability.

Measures of exposure, hazard, and vulnerability

We also learned how different measures of exposure, hazard and vulnerability fit the infection data. The simplest exposure index, which used seven-day recall of visits to a surface freshwater access point, fit infection intensity data better than did more complex exposure metrics that included information on the reported frequency, duration and extent of contact

experienced for specific activities. There are several potential explanations for this finding. This simple index was derived from a single survey question that was posed specifically for the children enrolled in the parasitology study. In contrast, the activity-specific estimates were drawn from a module that prompted individual-level information from all members of that household. Because of the volume of information that respondents were asked to provide (e.g., whether a person performed each of the six water contact activities for all members of the household), the accuracy and precision of the responses may have suffered.

Additionally, the activity-specific responses involved mean estimates of duration and extent of exposure that were applied across all children in the study without accounting for the variation in either measure. These measures assume consistent patterns of activity-specific duration and extent of contact across all households in all villages. They also involved attributing household-level frequency of each activity to the individual children living in that household, which may not be a safe assumption. Aggregating data to the household, village and study levels likely obscures important individual-level heterogeneities [36,40]. Because of the logistical challenge of comprehensively and precisely capturing water contact behavior for so many people, these methods represent our best approximation of the many facets of water contact behavior of our entire study population. Furthermore, incorporating these dimensions may have

introduced noise into the calculation of the activity-specific exposure metrics, making the simplest metric based on a single survey question the best measure of exposure.

Other studies have found weak or no association with exposure measures that account for multiple aspects of exposure. One study in a similar setting in northern Senegal found all of their exposure indices to be correlated with each other but not with the presence or intensity of *S. mansoni* infection [37]. In the study from which we drew data on activity-specific duration of water contact, a relationship between water contact and intensity of *S. mansoni* infection was only detected after accounting for body surface area and the time of day of exposure [27]. In a study of *S. haematobium* infection in Zanzibar, a set of exposure indices that incorporated varying combinations of frequency, duration and body surface area were found to be positively associated with infection, but the strength of the association decreased with the complexity of the exposure index used [41]. Moreover, the parasite species studied, the socio-ecological context and the methods used to measure water contact may all confound the true relationship between water contact and infection.

The variability in the correlation between infection and measures of exposure may be an artifact of differences in the methods used to measure water contact or differences in the

environmental hazard in the sites where exposure was measured. The issues implicit in the choice of methods for measuring water contact (e.g., recall and/or social desirability bias for questionnaire-based studies and failure to capture contact at other sites for studies based on direct observations at a single site) may be resolved by combining questionnaire-based or activity diary methods with the use of wearable GPS data loggers to collect more accurate and precise data on the spatial and temporal dynamics of water contact behavior. Such methods have been used in China [42], Uganda [43] and Cameroon [44] and have been found to be widely acceptable [45]. We found wearable GPS data loggers to widely acceptable in focus group discussions in this study site in Senegal as well (unpublished data).

Accounting for the spatial relationship between people and the environmental hazard also improved the fit of *S. haematobium* presence models. Hazard indices scaled by the distance to the nearest water point fit the infection presence data better than the other hazard indices, though this was not the case for infection intensity data. Previous studies have found proximity to water access points to be an important explanatory variable of schistosome infection in endemic settings [41,46–49]. Another found that water contact was only related to re-infection after accounting for the spatial distance between people and the hazard in their environment [39].

Such proximity may be an important aspect of vulnerability that makes it difficult to avoid exposure to hazardous water.

Similarly, hazard indices that dealt with the site nearest to the child's household fit the infection presence data better than village-level aggregations of snail habitat. This finding contradicts studies that found schistosomiasis to be associated with human behavior and snail ecology primarily at the community level [50–52]. However, because water contact activity for most people tends to occur at just a one or two sites [51,52] and since distance to water is commonly associated with infection [47,48], it is possible that the distance-adjusted hazard indices capture the hazard where exposure is most likely to occur for people living in a given household.

Measures of association between components of risk and infection

We found that no single model best approximated either the presence or intensity of *S. haematobium* infection. Rather, a set of models comprised the evidence for our conclusions. Without a single best-approximating model, we used model averaging to generate point estimates and confidence intervals of the association between our two infection outcomes and all explanatory variables appearing in both model sets.

Associations between demographic control variables and infection were consistent across the two outcomes. These variables – particularly child sex and village location – had the strongest relationship with infection of all variables included in all models. This finding speaks to the importance of demographics in the dynamics of schistosome infection [53,54] and the need for analyses that examine heterogeneity across sex and geographic strata.

We found that exposure was associated with an increase in both infection presence and intensity, although the confidence interval for the estimate of infection presence included the null value. Consistent with our relative importance findings, these estimates support the notion that the amount of exposure influences the accumulation of worm burden more than the acquisition of infection. The substantial support we found for vulnerability as an explanatory variable of infection presence may approximate the presence (rather than amount) of exposure. Given the lifespan of adult schistosomes in the human host (3–10 years) [53,55,56], a single infection resulting from a single exposure may be present in the body for years until a person is treated.

Even though model-averaging found indices of vulnerability to have wide confidence intervals, there was suggestive evidence of a dose-response between the odds of infection and the number of activities for which a household uses surface water. Children living in households that

relied on surface water for multiple activities were more likely to be infected than those whose households relied on surface water for a single activity. While this may be the result of a lack of alternative sources of water, it is also important to note that water contact activities often have social value [43], making the use of surface water for social activities like laundry a matter of preference. It is also possible that available alternative sources of water may not suitable for particular activities, making surface water preferable in some cases [57,58].

The asset-based SES index was the poorest index of vulnerability (ΔAIC = 9.48 and 6.40 among vulnerability-only presence intensity models, respectively), outperformed by indices that represented access to and use of water and sanitation infrastructure. It is possible that SES is a relatively poor predictor of infection because there is not much variability in ownership of durable assets across our study population. Similarly, while the household dependence on surface water is not evenly distributed across SES quintiles, we found similar proportions of households in highest and lowest SES quintiles that depend on surface water to some degree for household needs. It is easy to assume that dependence on surface water will be highly correlated with SES, but that may not be the case when decisions to continue using surface water are made for other reasons. Similarly, because our study focuses on children who go to school and play with each other, the influence of SES on infection outcomes may not be as pronounced as they would be

among adults, where occupation often determines exposure. Consequently, SES, as we measured it, may not be a strong determinant of water contact or schistosome infection outcomes.

Overall, we found that a simple metric of exposure explained infection patterns in school-aged children better than more complex metrics. The poor fit of the complex exposure metrics may reflect our failure to capture individual-level heterogeneity in important dimensions of water contact, namely frequency, duration and extent of exposure, for which we used aggregated measurements. The logistical challenges of studying water contact behavior are well-known and may be resolved by using wearable GPS technology to more accurately and precisely measure the spatial and temporal dynamics of exposure. Finally, our measures of hazard were improved by accounting for snail habitat in the water access site nearest to the household, and in the case of models of *S. haematobium* presence, the best metric accounted for distance between households and the nearest water point. These methodological insights highlight the important role of spatial relationships in schistosomiasis risk and the value of incorporating spatial data and analysis into future research.

Conclusion

In summary, we find that hazard, as measured by the area of snail habitat in the water access point nearest a household, and vulnerability, in terms of surface water use and access to sanitation at the household level, most contribute to the acquisition of *S. haematobium* infections. In contrast, exposure, approximated by a self-reported categorical frequency of contact with surface water per week, and hazard most contributes to the accumulation of parasites in school-age children, and thus to infection intensity. Together, our findings underscore the importance of all three components of risk, which act together across time and space to facilitate the acquisition and accumulation of schistosome infections in an endemic setting with high incidence of post-treatment re-infection. They highlight how interventions to complement MDA may be strategically deployed to reduce the risk of post-treatment re-infection.

While our data are specific to the context in which we collected them, the insights about the interaction among different components of risk and their impact on different metrics of infection may apply in other endemic settings and generate new knowledge about the processes that lead to the occurrence of persistent hot spots. Such an approach is critical to both controlling the severe morbidity of schistosomiasis and reducing the transmission of parasites in the environment to achieving elimination in endemic regions. Moreover, building a more holistic

understanding of infectious disease risk will improve our ability to intervene at the point where a source of infection and a susceptible person meet.

Acknowledgements

The authors thank Dr. Seynabou Ndiaye and Dr. Tidiane Ly for their work caring for the health of the children enrolled in the parasitology study. The authors also thank the staff of CRB-EPLS who played critical roles in collecting and processing the parasitology data, the ecological data and the household survey data as well as providing logistical and administrative support of the field work. The authors also thank Ashley Hazel for her helpful comments on an early draft of this manuscript.

References

1. Cardona O-D, van Aalst MK, Birkmann J, Fordham M, McGregor G, Perez R, et al. Determinants of Risk: Exposure and Vulnerability. In: Field CB, Barros V, Stocker TF, Dahe Q, editors. Managing the Risks of Extreme Events and Disasters to Advance Climate Change Adaptation. Cambridge: Cambridge University Press; 2012. pp. 65–108. doi:10.1017/CBO9781139177245.005

2. McClure M, Diuk-Wasser M. Reconciling the Entomological Hazard and Disease Risk in the Lyme Disease System. International Journal of Environmental Research and Public Health. 2018;15: 1048. doi:10.3390/ijerph15051048

3. Porta M. A dictionary of epidemiology. 6th ed. Oxford, UK: Oxford University Press; 2015.

4. Jones KE, Patel NG, Levy MA, Storeygard A, Balk D, Gittleman JL, et al. Global trends in emerging infectious diseases. Nature. 2008;451: 990–993. doi:10.1038/nature06536

5. Hosseini PR, Mills JN, Prieur-Richard A-H, Ezenwa VO, Bailly X, Rizzoli A, et al. Does the impact of biodiversity differ between emerging and endemic pathogens? The need to separate the concepts of hazard and risk. Phil Trans R Soc B. 2017;372: 20160129. doi:10.1098/rstb.2016.0129

6. US EPA. Guidelines for Human Exposure Assessment Risk Assessment Forum. Washington, DC: US EPA; 2019 Oct p. 223. Report No.: EPA/100/B-19/001.

7. Hagenlocher M, Castro MC. Mapping malaria risk and vulnerability in the United Republic of Tanzania: a spatial explicit model. Popul Health Metrics. 2015;13: 1–14. doi:10.1186/s12963-015-0036-2

8. Kienberger S, Hagenlocher M. Spatial-explicit modeling of social vulnerability to malaria in East Africa. Int J Health Geogr. 2014;13: 29. doi:10.1186/1476-072X-13-29

9. World Health Organization. PCT databank. In: WHO [Internet]. World Health Organization; 2020 [cited 22 Apr 2020]. Available: http://www.who.int/neglected_diseases/preventive_chemotherapy/sch/en/

10. World Health Organization. WHO | Schistosomiasis. World Health Organization; 2016 [cited 25 Jan 2016]. Available: http://www.who.int/schistosomiasis/en/

11. Fenwick A, Rollinson D, Southgate V. Implementation of Human Schistosomiasis Control: Challenges and Prospects. Adv Parasitol. 2006;61. doi:10.1016/S0065-308X(05)61013-5

12. King CH, Bertsch D. Historical perspective: snail control to prevent schistosomiasis. PLoS Negl Trop Dis. 2015;9: e0003657. doi:10.1371/journal.pntd.0003657

13. Webster JP, Molyneux DH, Hotez PJ, Fenwick A. The contribution of mass drug administration to global health: past, present and future. Philosophical Transactions of the Royal Society of London B: Biological Sciences. 2014;369: 20130434. doi:10.1098/rstb.2013.0434

14. Kittur N, King CH, Jr CHC, Kinung'hi S, Mwinzi PNM, Karanja DMS, et al. Persistent Hotspots in Schistosomiasis Consortium for Operational Research and Evaluation Studies for Gaining and Sustaining Control of Schistosomiasis after Four Years of Mass Drug Administration of Praziquantel. The American Journal of Tropical Medicine and Hygiene. 2019;101: 617–627. doi:10.4269/ajtmh.19-0193

15. Ross AGP, Olveda RM, Chy D, Olveda DU, Li Y, Harn DA, et al. Can mass drug administration lead to the sustainable control of schistosomiasis? Journal of Infectious Diseases. 2015;211: 283–289. doi:10.1093/infdis/jiu416

16. Sow S, de Vlas SJ, Engels D, Gryseels B. Water-related disease patterns before and after the construction of the Diama dam in northern Senegal. Annals of Tropical Medicine & Parasitology. 2002;96: 575–586. doi:10.1179/000349802125001636

17. Sokolow SH, Huttinger E, Jouanard N, Hsieh MH, Lafferty KD, Kuris AM, et al. Reduced transmission of human schistosomiasis after restoration of a native river prawn that preys

on the snail intermediate host. Proc Natl Acad Sci U S A. 2015;112: 9650–9655. doi:10.1073/pnas.1502651112

18. Wood CL, Sokolow SH, Jones IJ, Chamberline AJ, Lafferty KD, Kuris AM, et al. Precision mapping of snail habitats provides a powerful indicator of human schistosomiasis transmission. Proceedings of the National Academic of Sciences. 2019;116: 23182–23191. doi:https://doi.org/10.1073/pnas.1903698116

19. Stelma FF, Talla I, Polman K, Niang M, Sturrock RF, Deelder AM, et al. Epidemiology of Schistosoma mansoni infection in a recently exposed community in northern Senegal. Am J Trop Med Hyg. 1993;49: 701–6. Available: http://europepmc.org/abstract/med/8279638

20. Meurs L, Mbow M, Vereecken K, Menten J, Mboup S, Polman K. Epidemiology of mixed Schistosoma mansoni and Schistosoma haematobium infections in northern Senegal. Int J Parasitol. 2012;42: 305–11. doi:10.1016/j.ijpara.2012.02.002

21. Connor D, Comas J, Macpherson H-G, Mateos L. Impact of small-holder irrigation on the agricultural production, food supply and economic prosperity of a representative village beside the Senegal River, Mauritania. Agricultural Systems. 2008;96: 1–15. doi:10.1016/j.agsy.2007.04.001

22. Sturrock RF. Schistosomiasis epidemiology and control: how did we get here and where should we go? Memórias do Instituto Oswaldo Cruz. 2001;96: 17–27. doi:10.1590/S0074-02762001000900003

23. Gryseels B, Polman K, Clerinx J, Kestens L. Human schistosomiasis. Lancet. 2006;368: 1106–18. doi:10.1016/S0140-6736(06)69440-3

24. Liu R, Dong H-F, Guo Y, Zhao Q-P, Jiang M-S. Efficacy of praziquantel and artemisinin derivatives for the treatment and prevention of human schistosomiasis: a systematic review and meta-analysis. Parasit Vectors. 2011;4: 201. doi:10.1186/1756-3305-4-201

25. Zwang J, Olliaro PL. Clinical Efficacy and Tolerability of Praziquantel for Intestinal and Urinary Schistosomiasis — A Meta-analysis of Comparative and Non-comparative Clinical

Trials. PLoS Neglected Tropical Diseases. 2014;8: e3286. doi:10.1371/journal.pntd.0003286

26. Lund C, Browder N. The estimation of areas of burns. Surgery Gynecology & Obstetrics. 1944;79: 352–358.

27. Sow S, de Vlas SJ, Stelma F, Vereecken K, Gryseels B, Polman K. The contribution of water contact behavior to the high Schistosoma mansoni Infection rates observed in the Senegal River Basin. BMC Infect Dis. 2011;11: 198. doi:10.1186/1471-2334-11-198

28. Mosteller RD. Simplified Calculation of Body-Surface Area. N Engl J Med. 1987;317: 1098–1098. doi:10.1056/NEJM198710223171717

29. Filmer D, Pritchett LH. Estimating Wealth Effects Without Expenditure Data—Or Tears: An Application To Educational Enrollments In States Of India. Demography. 2001;38: 115–132. doi:10.1353/dem.2001.0003

30. Vyas S, Kumaranayake L. Constructing socio-economic status indices: how to use principal components analysis. Health Policy Plan. 2006;21: 459–468. doi:10.1093/heapol/czl029

31. R Core Team. R: A language and environmental for statistical computing. Vienna, Austria: R Foundation for Statistical Computing; 2018. Available: https://www.R-project.org/

32. Bates D, Mächler M, Bolker B, Walker S. Fitting Linear Mixed-Effects Models Using **lme4**. J Stat Soft. 2015;67. doi:10.18637/jss.v067.i01

33. Brooks ME, Kristensen K, Benthem KJ van, Magnusson A, Berg CW, Nielsen A, et al. glmmTMB Balances Speed and Flexibility Among Packages for Zero-inflated Generalized Linear Mixed Modeling. The R Journal. 2017;9: 378–400. Available: https://journal.r-project.org/archive/2017/RJ-2017-066/index.html

34. Burnham KP, Anderson DR. Model Selection and Multimodel Inference: A Practical Information-Theoretic Approach. 2nd ed. New York: Springer; 2002.

35. Mazerolle, Marc J. AICcmodavg: Model selection and multimodel inference based on (Q)AIC(c). 2019. Available: https://cran.r-project.org/web/packages/AICcmodavg/AICcmodavg.pdf

36. Bundy DAP, Blumenthal UJ. Human behavior and the epidemiology of helminth infections: the role of behavior in exposure to infection. In: Barnard CJ, Behnke JM, editors. Parasitism and Host Behavior1. London: Taylor & Francis Ltd; 990. pp. 264–289.

37. Scott JT, Diakhate M, Vereecken K, Fall A, Diop M, Ly A, et al. Human water contacts patterns in Schistosoma mansoni epidemic foci in northern Senegal change according to age, sex and place of residence, but are not related to intensity of infection. Tropical Medicine and International Health. 2003;8: 100–108. doi:10.1046/j.1365-3156.2003.00993.x

38. Booth M, Vennervald BJ, Kabatereine NB, Kazibwe F, Ouma JH, Kariuki CH, et al. Hepatosplenic morbidity in two neighbouring communities in Uganda with high levels of Schistosoma mansoni infection but very different durations of residence. Transactions of the Royal Society of Tropical Medicine and Hygiene. 2004;98: 125–136. doi:10.1016/S0035-9203(03)00018-X

39. Seto E, Lee YJ, Liang S, Zhong B. Individual and village-level study of water contact patterns and Schistosoma japonicum infection in mountainous rural China. Tropical Medicine & International Health. 2007;12: 1199–1209. doi:10.1111/j.1365-3156.2007.01903.x

40. Susser M. Does risk factor epidemiology put epidemiology at risk? Peering into the future. Journal of epidemiology and community health. 1998;52: 608–11. doi:10.1136/JECH.52.10.608

41. Rudge JW, Stothard JR, Basáñez M-G, Mgeni AF, Khamis IS, Khamis AN, et al. Micro-epidemiology of urinary schistosomiasis in Zanzibar: Local risk factors associated with distribution of infections among schoolchildren and relevance for control. Acta Trop. 2008;105: 45–54. doi:10.1016/j.actatropica.2007.09.006

42. Seto E, Knapp F, Zhong B, Yang C. The use of a vest equipped with a global positioning system to assess water-contact patterns associated with schistosomiasis. Geospat Health. 2007;1: 233–41. doi:10.4081/gh.2007.271

43. Seto E, Sousa-Figueiredo JC, Betson M, Byalero C, Kabatereine NB, Stothard JR. Patterns of intestinal schistosomiasis among mothers and young children from Lake Albert, Uganda: water contact and social networks inferred from wearable global positioning system dataloggers. Geospatial health. 2012;7: 1–13. doi:10.4081/gh.2012.99

44. Macklin G, Stanton MC, Tchuem-Tchuenté LA, Stothard JR. A pilot study using wearable global positioning system data loggers to compare water contact levels: Schistosoma haematobium infection in pre-school-age children (PSAC) and their mothers at Barombi Kotto, Cameroon. Transactions of The Royal Society of Tropical Medicine and Hygiene. 2018 [cited 15 Jul 2018]. doi:10.1093/trstmh/try059

45. Paz-Soldan VA, Stoddard ST, Vazquez-Prokopec G, Morrison AC, Elder JP, Kitron U, et al. Assessing and maximizing the acceptability of global positioning system device use for studying the role of human movement in dengue virus transmission in Iquitos, Peru. Am J Trop Med Hyg. 2010;82: 723–30. doi:10.4269/ajtmh.2010.09-0496

46. Kloos H, Fulford AJC, Butterworth AE, Sturrock RF, Ouma JH, Kariuki HC, et al. Spatial patterns of human water contact and Schistosoma mansoni transmission and infection in four rural areas in Machakos District, Kenya. Social Science & Medicine. 1997;44: 949–968. doi:10.1016/S0277-9536(96)00218-3

47. Pennance T, Person B, Muhsin MA, Khamis AN, Muhsin J, Khamis IS, et al. Urogenital schistosomiasis transmission on Unguja Island, Zanzibar: characterisation of persistent hot-spots. Parasites & Vectors. 2016;9. doi:10.1186/s13071-016-1847-0

48. Clennon JA, King CH, Muchiri EM, Kariuki HC, Ouma JH, Mungai P, et al. Spatial patterns of urinary schistosomiasis infection in a highly endemic area of coastal Kenya. Am J Trop Med Hyg. 2004;70: 443–448. Available: http://www.ajtmh.org/content/70/4/443.full

49. Walker JW, Kittur N, Binder S, Castleman JD, Drake JM, Campbell CH, et al. Environmental Predictors of Schistosomiasis Persistent Hotspots following Mass Treatment with Praziquantel. The American Journal of Tropical Medicine and Hygiene. 2020;102: 328–338. doi:10.4269/ajtmh.19-0658

50. Raso G, Matthys B, N ’goran EK, Tanner M, Vounatsou P, Utzinger J. Spatial risk prediction and mapping of Schistosoma mansoni infections among schoolchildren living in western Côte d’Ivoire. Parasitology. 2005;131: 97–108. doi:10.1017/S0031182005007432

51. Woolhouse MEJ, Watts CH, Chandiwana SK. Heterogeneities in transmission rates and the epidemiology of schistosome infection. Proceedings of the Royal Society of London Series B: Biological Sciences. 1991;245: 109–114. doi:10.1098/rspa.1983.0054

52. Woolhouse ME, Etard JF, Dietz K, Ndhlovu PD, Chandiwana SK. Heterogeneities in schistosome transmission dynamics and control. Parasitology. 1998;117: 475–482. doi:10.1017/S003118209800331X

53. Bustinduy AL, King CH. Schistosomiasis. Manson’s Tropical Infectious Diseases. Elsevier; 2014. pp. 698-725.e6. doi:10.1016/B978-0-7020-5101-2.00053-4

54. Huang Y, Manderson L. Schistosomiasis and the social patterning of infection. Acta Trop. 1992;51: 175–194. doi:10.1016/0001-706X(92)90037-X

55. Goddard MJ, Jordan P. On the longevity of Schistosoma mansoni in man on St. Lucia, West Indies. Transactions of the Royal Society of Tropical Medicine and Hygiene. 1980;74: 185–191. doi:10.1016/0035-9203(80)90240-0

56. Fulford AJC, Butterworth AE, Ouma JH, Sturrock RF. A statistical approach to schistosome population dynamics and estimation of the life-span of *Schistosoma mansoni* in man. Parasitology. 1995;110: 307–316. doi:10.1017/S0031182000080896

57. Krauth SJ, Musard C, Traoré SI, Zinsstag J, Achi LY, N’Goran EK, et al. Access to, and use of, water by populations living in a schistosomiasis and fascioliasis co-endemic area of

northern Côte d'Ivoire. Acta Trop. 2015;149: 179–85. doi:10.1016/j.actatropica.2015.05.019

58. Kulinkina AV, Kosinski KC, Adjei MN, Osabutey D, Gyamfi BO, Biritwum N-K, et al. Contextualizing Schistosoma haematobium transmission in Ghana: assessment of diagnostic techniques and individual and community water-related risk factors. Acta Tropica. 2019 [cited 18 Mar 2019]. doi:10.1016/j.actatropica.2019.03.016

Supporting Information

S1 Figure. Data flow diagram. Sample sizes resulting from the merging and cleaning of human data from parasitological and household surveys and removing data from manipulated sites.

S1 Table. Household survey items. Household survey items used in in this analysis, including the module to which an item belonged, the level at which it was measured as well as the wording of and response categories provided for each question

S2 Table. Duration and extent of water contact. Activity-specific values used to estimate duration and extent of water contact. Duration estimates were drawn from published research that took place in a similar setting and extent was estimated from body surface area interviews.

S3 Table. Household surface water use by socio-economic status. Frequencies (n (row %)) of household surface water use by quintiles of asset-based index of socio-economic status (SES).

S4 Table. Presence models. Full set of candidate models used for multi-model inference of the relative contributions of exposure, hazard and vulnerability to *S. haematobium* infection presence.

S5 Table. Intensity models. Full set of candidate models used for multi-model inference of the relative contributions of exposure, hazard and vulnerability to *S. haematobium* infection intensity.

S1 Checklist. STROBE checklist for cross-sectional studies

Figure 1

Click here to access/download;Figure;Fig1_150.tiff

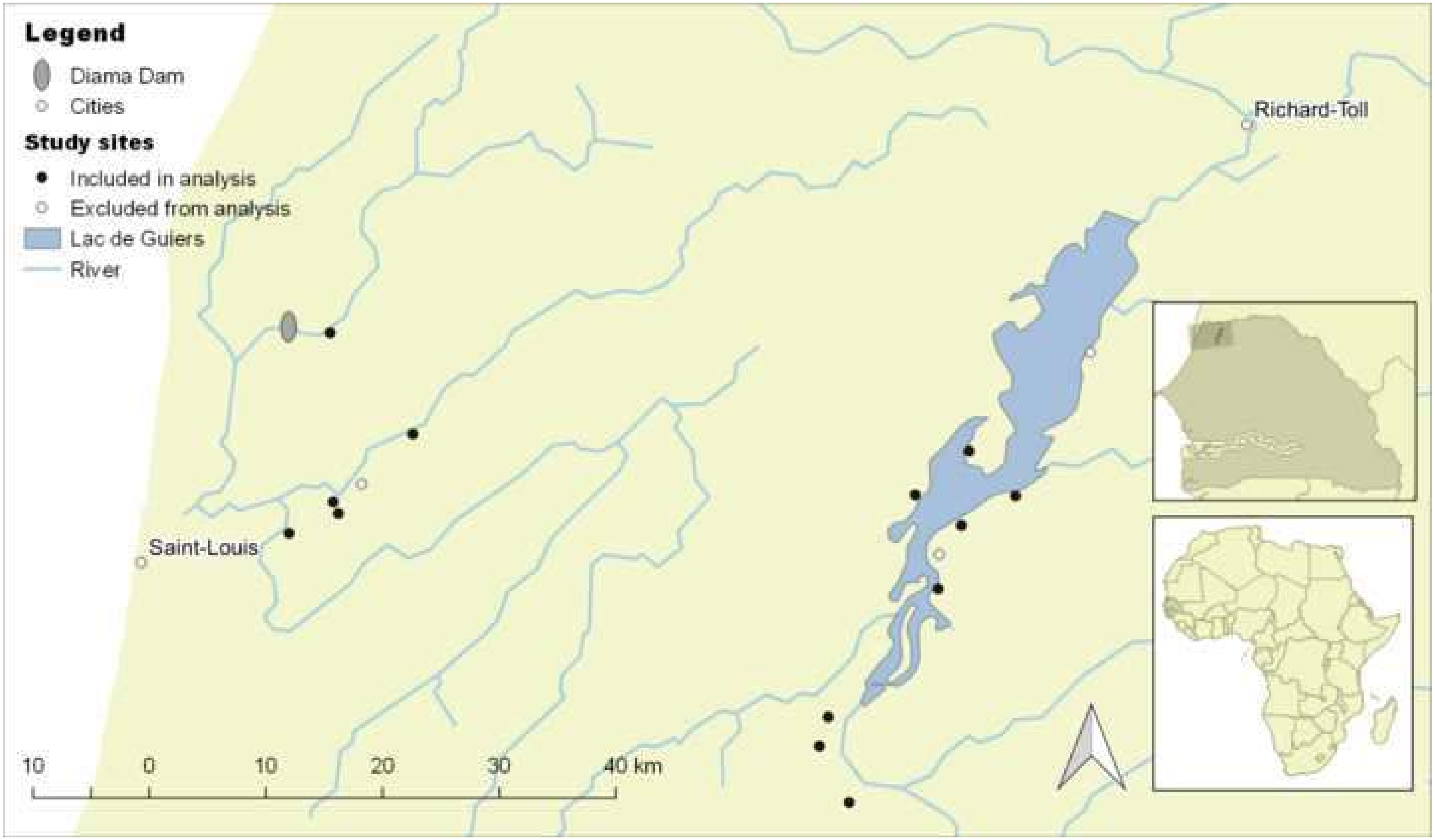

Figure 2

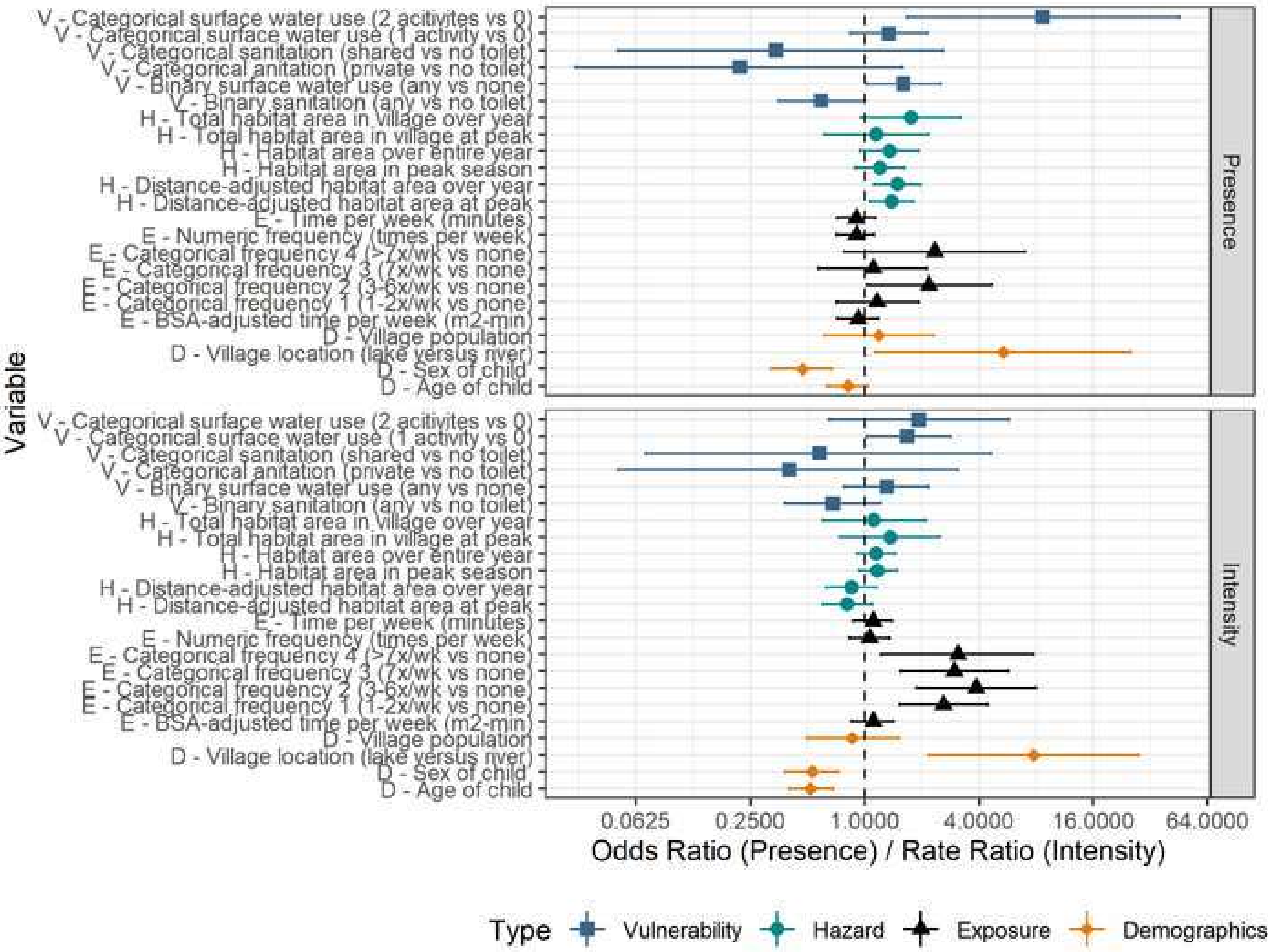
V - Categorical surface water use (2 acitivites vs 0)
V - Categorical surface water use (1 activity vs 0)
V - Categorical sanitation (shared vs no toilet)
V - Categorical anitation (private vs no toilet)
V - Binary surface water use (any vs none)
V - Binary sanitation (any vs no toilet)
H - Total habitat area in village over year
H - Total habitat area in village at peak
H - Habitat area over entire year
H - Habitat area in peak season
H - Distance-adjusted habitat area over year
H - Distance-adjusted habitat area at peak
E - Time per week (minutes)
E - Numeric frequency (times per week)
E - Categorical frequency 4 (>7x/wk vs none)
E - Categorical frequency 3 (7x/wk vs none)
E - Categorical frequency 2 (3-6x/wk vs none)
E - Categorical frequency 1 (1-2x/wk vs none)
E - BSA-adjusted time per week (m2-min)
D - Village population
D - Village location (lake versus river)
D - Sex of child
D - Age of child
Variable
Presence
Intensity
0.0625
0.2500
1.0000
4.0000
16.0000
64.0000
Odds Ratio (Presence) / Rate Ratio (Intensity)
Type
Vulnerability
Hazard
Exposure
Demographics

Click here to access/download
Supporting Information
S1 Figure.tif

Click here to access/download
Supporting Information
S1 Table.pdf

Click here to access/download
Supporting Information
S2 Table.pdf

Click here to access/download
Supporting Information
S3 Table.pdf

Click here to access/download
Supporting Information
S4 Table.xlsx

Click here to access/download
Supporting Information
S5 Table.xlsx
