# Supplementary material for: Exposure, hazard, and vulnerability all contribute to Schistosoma haematobium re-infection in northern Senegal
Source: PLoS Negl Trop Dis. 2021 Oct 5;15(10):e0009806. doi: 10.1371/journal.pntd.0009806 (PMC8525765; doi:10.1371/journal.pntd.0009806)
Supplement: Attachment — Submitted filename: Lund_EHV_responseLetter.pdf [file pntd.0009806.s010.pdf]

Dr. Poppy H. Lamberton
Deputy Editor
Dr. Anna Last
Associate Editor
PLOS Neglected Tropical Diseases

Dear Dr. Lamberton and Dr. Last:

Thank you for considering our manuscript (PNTD-D-20_02100) “Exposure, hazard, and vulnerability all contribute to *Schistosoma haematobium* re-infection in northern Senegal” for publication in PLOS Neglected Tropical Diseases. We thank you and all four reviewers for your help in greatly improving the manuscript. We have revised the manuscript according to the reviewers’ comments and believe that the revised manuscript clarifies both the strengths and limitations of the paper as well as its novelty in bringing together primary data collection across the social and environmental dimensions of schistosome infection risk.

Specifically, we have consolidated the limitations of our study under a new “Limitations” subheading in the Discussion. We have also further supported statements in both the Introduction and Discussion sections with additional references and clarified several methodological details. You will find a point-by-point response of how we addressed each of the reviewers’ comments below.

We reiterate that this manuscript is original and is not being considered for publication elsewhere. We have no conflicts of interest to disclose.

Thank you for consideration.

Sincerely,

Andrea Lund, PhD MPH

Response to reviews

Thanks for this interesting paper which we would be happy to see resubmitted after undertaking the recommended changes. To me this reads as a mixed methods paper and could be sold more so on this aspect and the benefits that this provides, also helping to address the issues with smaller sample sizes, that would not be satisfactory for a purely quantitative study.

Response: Thank you for these comments. We have revised the manuscript according to these and additional suggestions below.

Reviewer's Responses to Questions

Key Review Criteria Required for Acceptance
As you describe the new analyses required for acceptance, please consider the following:

Methods
-Are the objectives of the study clearly articulated with a clear testable hypothesis stated?
-Is the study design appropriate to address the stated objectives?
-Is the population clearly described and appropriate for the hypothesis being tested?
-Is the sample size sufficient to ensure adequate power to address the hypothesis being tested?
-Were correct statistical analysis used to support conclusions?
-Are there concerns about ethical or regulatory requirements being met?

Reviewer #1: yes

Reviewer #2: (No Response)

Reviewer #3: Overall, the methods are appropriately chosen in response to the study questions/objectives. The only minor issues is related to interview described in the study. Only two interviews/village with a total of 30 interview were conducted – what are potential implications with the analysis with such a few samples? Also unclear how 210 activity-specific observations were conducted?

Reviewer #4: Methods are explained in detail. Some limitations with the use of existing data collected from the communities is discussed.

Response: Thank you for noting these important methodological details and limitations that were missing from the text. We have added a brief detail to clarify our description of the interview data collection in the Methods section (see line 258-259 in the tracked changes version), where we have clarified that the 210 activity-specific observations arise from interviewing 30 respondents about 7 water contact activities. We discuss the small sample size for interviews as a limitation of these data in the Discussion (see lines 876-928 in the tracked changes version).

Results
-Does the analysis presented match the analysis plan?
-Are the results clearly and completely presented?
-Are the figures (Tables, Images) of sufficient quality for clarity?

Reviewer #1: The authors ignore many relevant papers that have been published, which are given in a sticky note on the returned ms. They completely ignore the fact that very few snails have to be infected to result in a high (>87%) of school age children. They record the number of times that the children were in the water but they do not record the time of day they had water contact, which is extremely important.

Reviewer #2: (No Response)

Reviewer #3: Well done!

Reviewer #4: The results are clearly outlined and address the aims (integrated consideration of hazards, exposure and vulnerability).
Some of the tables and figures could/should have acronyms described more clearly in headers and/or legends.

Response: We thank all four reviewers for their constructive comments.

We have added relevant citations to both the Introduction (lines 83-84, 119-130 in the tracked changes version) and Discussion (lines 671-675, 736- 773, 890-895 in the tracked changes version). We found the references suggested by Reviewer 1 to be very interesting pieces of research and incorporated a few where appropriate, including Madsen et al.'s 2011 paper in EcoHealth, which reports high levels of human infection despite snails occurring in very low numbers.

The reviewer makes a great point, and we strongly agree that the time of day is an important element of exposure, especially considering the chronobiology of cercarial release. Our questionnaire-based methods captured typical, regular behavior involving contact with water, rather than individual episodes of water contact. This limited our ability to account for the time of day of exposure in a meaningful or reliable way, despite its importance. When designing this study, we grappled with the trade-offs between collecting full information (frequency, duration, time of day) on the exposure of a small number of people versus collecting less complete information on a larger proportion of the population when designing this study. Obviously, we opted for the latter, measuring exposure among all school-aged children in our study population through a household survey, and incorporating additional data from the literature, interviews, and household-level survey questions to approximate some additional dimensions of water contact and exposure. This choice ultimately limited our ability to capture the time of day, out of concern that survey respondents would not be able to reliably recall the time of day for many individual episodes of water contact that may have occurred over the preceding week(s). This is a limitation of the methods we chose to use, which we have further noted in the Discussion (see lines 876-928 in the tracked changes version)

Additionally, we have added detail to captions of both figure and tables to improve clarity.

Conclusions
-Are the conclusions supported by the data presented?
-Are the limitations of analysis clearly described?
-Do the authors discuss how these data can be helpful to advance our understanding of the topic under study?
-Is public health relevance addressed?

Reviewer #1: There conclusions ignore important literature and thus they make sweeping statements that are not supported by other studies. Over all they have an excellent paper, they just need to add additional references.

Reviewer #2: (No Response)

Reviewer #3: Overall well done.

Reviewer #4: Conclusions are sounds. The discussion of weaknesses in the data at every step is commendable but detracts from the significance of their findings.
The authors clearly discuss what could be taken away from this study for efforts to disrupt the parasite lifecycle

Response: We appreciate the Reviewer 1 noting gaps in the literature we cited, both here and in the Introduction. As stated above, we have incorporated new citations in both sections of the revised manuscript. We also have consolidated methodological limitations of our study in a single section (with its own subheading) in the Discussion (see lines 876-928 in the tracked changes version)

Editorial and Data Presentation Modifications?
Use this section for editorial suggestions as well as relatively minor modifications of existing data that would enhance clarity. If the only modifications needed are minor and/or editorial, you may wish to recommend "Minor Revision" or "Accept".

Reviewer #1: (No Response)

Reviewer #2: (No Response)

Reviewer #3: Accept

Reviewer #4: See comments below

Summary and General Comments
Use this section to provide overall comments, discuss strengths/weaknesses of the study, novelty, significance, general execution and scholarship. You may also include additional comments for the author, including concerns about dual publication, research ethics, or publication ethics. If requesting major revision, please articulate the new experiments that are needed.

Reviewer #1: The title indicates it is a study of re-infection, which is misleading. In order to measure re--infection they would have to identify infected children, treat them, re-test them, and then have them exposed to water and determine re-infection

Response:
Thank you for pointing this out. The reviewer is right that our study does not meet the strict definition of re-infection, as they define it. We did not have the ability to track all exposure in all children over the course of a year. However, the data we report are a cross-section of a longitudinal study in which all children were tested in Spring 2016 to confirm infection, received double dose praziquantel treatment. They then were retested (and treated again) in Spring 2017 and Spring 2018 to estimate reinfection rates a year after treatment. Therefore, the infection data we report in our analysis reflect post-treatment infections acquired over the preceding year. Though we do not confirm that all observed infections were cleared of infection (parasitological cure) by treatment in that year, the assumed efficacy of praziquantel followed by the intensity of exposure is enough in this setting that we think that the use of the term "re-infection" is warranted. Most of the children in our study were infected at baseline and most of them remained infected in subsequent years. We have added language to clarify this point in the Methods (see lines 226-230 in the tracked changes version)

Reviewer #2: In this paper, Lund and colleagues address the interesting question of the interaction between hazard, exposure and vulnerability associated with infection and infection intensity of S. haematobium in Senegal. They found that infection presence was mostly driven hazard and vulnerability, while infection intensity is determined by exposure and hazard. I think this is an interesting paper that deserves to be published in PLoS NTDs when my comments would have been addressed.

I see two main concerns with the statistical analysis. First, they select one variable for each component (exposure, vulnerability and hazard), and then perform model selection to identify which one is the most parcimonious. Doing things like that can introduce biases. Indeed, if two variables included in their "hazard" category could explain the whole pathogen presence (or intensity), this cannot be identified because only one variable by component is selected and confronted to the others. I understand the rationale behind that, but I think it would be more robust to perform the analysis without such variable grouping.

Second, in the case where they select only one variable by group, I think including the interactions between variables could be interesting, since exposure and hazard could definitely interact between them.

On a minor note, from table 5, I don't understand the difference between model 16 and 19.

Response:
Thank you for these thoughtful comments and suggestions.

Indices within a group (i.e., exposure, hazard, or vulnerability) were highly correlated with each other. Three of the four exposure indices, for example, had correlation coefficients approaching 1. Dichotomous and categorical variables of surface water use were also positively correlated, as were snail habitat areas calculated for the peak transmission season and over the entire year. To clarify this point, we have added a correlation plot to our supplementary files that illustrates bivariate correlations between all variables included in our analysis. We believe that variables within a group represent slight variations in the measurement of the same construct within the exposure, hazard, and vulnerability framework. including multiple indices from the same group would likely introduce issues of collinearity and artificially inflate variance estimates. Additionally, specifying a "full" model that includes all possible explanatory variables is not possible with our relatively small sample size, as there are not enough degrees of freedom to support model convergence. For these reasons, we believe the approach that we've taken – including one variable from each group – is the best way to address the question we set out to answer.

Regarding interaction terms, we agree that the concepts of exposure, hazard, and vulnerability interact in determining risk for schistosome infection! In the initial stages of conducting this analysis, we fit models with interaction terms between exposure, hazard and/or vulnerability variables. When we did this, we found that the AIC nearly always increased for models with interactions terms compared to identical models without interaction terms. That is, interaction terms did not improve the fit of models in either set. Moreover, adding interaction terms complicated the visualization and interpretation of our model averaging results. With an interaction term, for example, an odds ratio representing the association between infection presence and exposure, for example, would have to be estimated across all levels of the interacting hazard or vulnerability variable. Visualizing these stratified estimates in Figure 2, which is already quite busy, would complicate the interpretation and sacrifice the clarity of our findings without generating much additional insight. Given this, we do not feel that including interaction terms adds value to our analysis.

Finally, thank you for pointing out the confusion in Table 5. These reflect two models that included indices for both exposure and hazard, but they differed in which hazard indices were included in each model. In Model 16, the hazard index was the area of snail habitat during the peak season (areaPeak), while in Model 19, the hazard index was the same measure of snail habitat area scaled by distance between the water access site and the child's household (areaPeak_d). The full details on both sets of models can be found in S4 Table (presence) and S5 table (intensity). These supplementary tables are large, and we felt it was more appropriate to report them in supplementary material than in the main text. Table 5 was meant to efficiently summarize the inclusion of exposure, hazard, and vulnerability as variables in the best-fitting models in each set. We reference the supplementary tables in Table 5 to direct the interested reader to the full details of each set of models.

Reviewer #3: (No Response)

Reviewer #4: This research article highlights the importance of environmental hazards, exposure and vulnerability on the risks of urogenital schistosomiasis affecting school-aged children in a region of northern Senegal. Their findings extend work that has primarily focused on the demographic features of a population that affect the risk of infection. Consideration for each component of risk seems highly logical but rarely reported in an integrated way as they have reported here. This is a key, novel feature of this article (in my opinion). Discussion highlights several weaknesses in the approach used herein. Although this has limited the power of the analyses (as mentioned in the discussion), it also highlights that there is a lot more work to do in planning future epidemiological work. Overall, the methods look quite comprehensive. I was unable to review the DRYAD information using the url provided for dryad.s7h44j15p. Is it publicly available? You mention three studies
that were undertaken to obtain the data used herein. Some abbreviation of previously published methods may be warranted here. Please make sure they are cited, similar to the PNAS manuscript (reference 18). This will make it clear what methods are unique to this manuscript.
I have some other suggestions that I think are worth considering.

The introduction suggests a broad range of hazards should be considered, including floods and extreme weather events. Despite this being a focus in the introduction, it wasn't a focus on the analysis and no data reported on extreme/catastrophic events in the region studied during the time of sampling. For this reason, I suggest simplifying the introduction to focus on the risks identified in a more usual seasonal cycle in the region (as presented in the results and discussed).

Narrative in the introduction can be confusing. I would consider starting with schistosomiasis or significantly reduce the background at the start of the introduction. I don't think an extended justification for looking at risk (exposure, hazard, and vulnerability) is required (start of the introduction). Most readers would be aware of this topic. Brief explanation would be better with an introduction that is focussed on the subject (schistosomiasis).

Lines 144 to 147. Please make it clear in the introduction why demographic features were included as control here. It is in the methods, results and discussion, but the approach could be better presented in the last paragraph of the discussion.

Line 222 to 223. Something missing in this sentence
Lines 529 to 532.Please include references to support these statements.
Line 618 and Line 648. Manuscript is well written but please check for grammatical errors throughout

Discussion. Would it be better to incorporate the limitations of this study into one section and discuss how this can be resolved in the future. It feels repetitive at times and detracts from the significant results obtained in this study.

Response:

Thank you for the praise and constructive comments.

We apologize for the confusion about the Dryad dataset. The data are privately available for peer review. We meant to share the temporary "Private for Peer Review" link but mistakenly provided the public doi link (which is not yet active) in our initial submission. We have included the correct link below. Reviewers should be able to find the data set (aggregated to village level to conform with human subjects protections for individual-level infection data) at this temporary link for peer review purposes: https://datadryad.org/stash/share/7i3cLq46DaaD2eGw7FTsIGP8lbBL1VStyWlxMi9WL0Q

We cited all studies from which we drew data or methods. Body surface area exposed was measured through interviews employing a diagram for measuring burn size (reference 44) and calculations for body surface area (reference 45). The duration of exposure for six common water contact activities was drawn from data reported in reference 39. The index of socio-economic status was calculated based on methods described in references 46 and 47. Aside from these and the hazard metrics developed from data also reported in reference 33, the data and the methods used to collect them are unique to this manuscript and have not been published elsewhere.

In the Introduction, we don't mean to imply that flooding and extreme weather are immediately relevant to schistosomiasis risk, only to highlight a related field that readily employs the "exposure-hazard-and vulnerability" framework. We think this framing lends itself well to the risk of environmentally-mediated infectious diseases, including schistosomiasis, but found very few papers on the subject that use it. We feel it's important to remain in conversation with the distinct but related fields that use the constructs of exposure, hazard, and vulnerability to understand risk of adverse events. For this reason, we have retained the justification at the beginning of the Introduction.

We have added language and citations to the end of the Introduction regarding the inclusion of demographic characteristics as control variables (see lines 172-173 in the tracked changes version)

We have corrected the errors identified the reviewer identified in lines 222-223, 618 and 648 of the original version of the manuscript. Thank you for spotting these! We have also supported the statements in lines 529-532 (now line 670-675 with tracked changes) with references and additional text.

Finally, as stated previously, we have consolidated methodological limitations of our study in a single section (with its own subheading) in the Discussion (see lines 876-928 in the tracked changes version).
