# Supplementary material for: Exposure, hazard, and vulnerability all contribute to Schistosoma haematobium re-infection in northern Senegal
Source: PLoS Negl Trop Dis. 2021 Oct 5;15(10):e0009806. doi: 10.1371/journal.pntd.0009806 (PMC8525765; doi:10.1371/journal.pntd.0009806)
Supplement: Attachment — Submitted filename: revision senegal.pdf [file pntd.0009806.s011.pdf]

PLOS Neglected Tropical Diseases

Exposure, hazard, and vulnerability all contribute to Schistosoma haematobium re-infection in northern Senegal

--Manuscript Draft--

**Manuscript Number:**	PNTD-D-20-02100R1
**Full Title:**	Exposure, hazard, and vulnerability all contribute to Schistosoma haematobium re-infection in northern Senegal
**Short Title:**	Exposure, hazard, and vulnerability in S. haematobium re-infection in Senegal
**Article Type:**	Research Article
**Keywords:**	Schistosoma haematobium; Senegal; exposure; hazard; vulnerability; infectious disease risk; socio-ecological systems; multi-model inference
**Abstract:**	Background: Infectious disease risk is driven by three interrelated components: exposure, hazard, and vulnerability. For schistosomiasis, exposure occurs through contact with water, which is often tied to daily activities. But water contact does not imply risk unless the environmental hazard of snails and parasites is also present in the water. By increasing reliance on hazardous activities and environments, socio-economic vulnerability can hinder reductions in exposure to a hazard. We aimed to quantify the contributions of exposure, hazard, and vulnerability to the presence and intensity of Schistosoma haematobium re-infection.<br>Methodology/Principal Findings: In 13 villages along the Senegal River, we collected parasitological data from 821 school-aged children, survey data from 411 households where those children resided, and ecological data from all 24 village water access sites. We fit mixed-effects logistic and negative binomial regressions with indices of exposure, hazard, and vulnerability as explanatory variables of Schistosoma haematobium presence and intensity, respectively, controlling for demographic variables. Using multi-model inference to calculate the relative importance of each component of risk, we found that hazard (Σw_i = 0.95) was the most important component of S. haematobium presence, followed by vulnerability (Σw_i = 0.91). Exposure (Σw_i = 1.00) was the most important component of S. haematobium intensity, followed by hazard (Σw_i = 0.76). Model averaging quantified associations between each infection outcome and indices of exposure, hazard, and vulnerability, revealing a positive association between hazard and infection presence (OR = 1.49, 95% CI 1.12, 1.97), and a positive association between exposure and infection intensity (RR 2.59 – 3.86, depending on the category; all 95% CIs above 1)<br>Conclusions/Significance: Our findings underscore the linkages between social (exposure and vulnerability) and environmental (hazard) processes in the acquisition and accumulation of S. haematobium infection. This approach highlights the importance of implementing both social and environmental interventions to complement mass drug administration.
**Additional Information:**	
**Question**	**Response**
**Financial Disclosure**<br><br>Enter a financial disclosure statement that describes the sources of funding for the work included in this submission. Review the submission guidelines for detailed requirements. View published research articles from *PLOS NTDs* for specific examples.<br><br>This statement is required for submission and **will appear in the published article** if	The study received funding from the National Science Foundation Couple Natural Human Systems program (BCS-1414102 to SHS and DLC) and the Bill and Melinda Gates Foundation (OPP1114050 to SHS and GADL). AJL was supported by a James and Nance Kelso Fellowship through the Stanford Interdisciplinary Graduate Fellowship (SIGF) program at Stanford University. GADL, SHS and AJL were also partially supported by the National Science Foundation (DEB-2011179 to GADL and Belmont CEH/NSF ICER-2024383 to GADL). IJJ was funded by National Science Foundation Graduate Research Fellowship (#1656518). CLW was supported by the Michigan Society of Fellows at the University of Michigan, a Sloan Research Fellowship from the Alfred P. Sloan Foundation, a UW Innovation Award and a grant from the National Science Foundation (OCE-1829509 to CLW). SA was funded by the King Center on Global Development Field Research Assistant Program. The funders had no role in study design, data collection, interpretation or the decision to submit the

the submission is accepted. Please make sure it is accurate.<br><br>**Unfunded studies**<br><br>Enter: *The author(s) received no specific funding for this work.*<br><br>**Funded studies**<br><br>Enter a statement with the following details:<br>• Initials of the authors who received each award<br>• Grant numbers awarded to each author<br>• The full name of each funder<br>• URL of each funder website<br>• Did the sponsors or funders play any role in the study design, data collection and analysis, decision to publish, or preparation of the manuscript?<br>• **NO** - Include this sentence at the end of your statement: *The funders had no role in study design, data collection and analysis, decision to publish, or preparation of the manuscript.*<br>• **YES** - Specify the role(s) played.<br><br>* typeset	work for publication.
**Competing Interests**<br><br>Use the instructions below to enter a competing interest statement for this submission. On behalf of all authors, disclose any competing interests that could be perceived to bias this work—acknowledging all financial support and any other relevant financial or non-financial competing interests.<br><br>This statement **will appear in the published article** if the submission is accepted. Please make sure it is accurate. View published research articles	The authors have declared that no competing interests exist.

Powered by Editorial Manager® and ProduXion Manager® from Aries Systems Corporation

from *PLOS NTDs* for specific examples.<br><br>**NO authors have competing interests**<br><br>Enter: *The authors have declared that no competing interests exist.*<br><br>**Authors with competing interests**<br><br>Enter competing interest details beginning with this statement:<br><br>*I have read the journal's policy and the authors of this manuscript have the following competing interests: [insert competing interests here]*<br><br>* typeset<br><br>This statement is required for submission and **will appear in the published article** if the submission is accepted. Please make sure it is accurate and that any funding sources listed in your Funding Information later in the submission form are also declared in your Financial Disclosure statement.	
**Data Availability**<br><br>Authors are required to make all data underlying the findings described fully available, without restriction, and from the time of publication. PLOS allows rare exceptions to address legal and ethical	No - some restrictions will apply

concerns. See the PLOS Data Policy and FAQ for detailed information.<br><br>A Data Availability Statement describing where the data can be found is required at submission. Your answers to this question constitute the Data Availability Statement and **will be published in the article**, if accepted.<br><br>**Important:** Stating 'data available on request from the author' is not sufficient. If your data are only available upon request, select 'No' for the first question and explain your exceptional situation in the text box.<br><br>Do the authors confirm that all data underlying the findings described in their manuscript are fully available without restriction?	
**Describe where the data may be found in full sentences. If you are copying our sample text, replace any instances of** XXX **with the appropriate details.**<br><br>• If the data are **held or will be held in a public repository**, include URLs, accession numbers or DOIs. If this information will only be available after acceptance, indicate this by ticking the box below. For example: *All XXX files are available from the XXX database (accession number(s) XXX, XXX.*).<br>• If the data are all contained **within the manuscript and/or Supporting Information files**, enter the following: *All relevant data are within the manuscript and its Supporting Information files.*<br>• If neither of these applies but you are able to provide **details of access**	Individual- and household-level data needed to replicate the analysis cannot be shared publicly because of IRB privacy safeguards, but can be made available by contacting the IRB at the University of California Santa Barbara (hsc@research.ucsb.edu) and Stanford University (irbnonmed@stanford.edu) with a reasonable request. Data aggregated to the village level can be found in the following Dryad repository: https://doi.org/10.5061/dryad.s7h44j15p

**elsewhere**, with or without limitations, please do so. For example:<br><br>*Data cannot be shared publicly because of [XXX]. Data are available from the XXX Institutional Data Access / Ethics Committee (contact via XXX) for researchers who meet the criteria for access to confidential data.*<br><br>*The data underlying the results presented in the study are available from (include the name of the third party and contact information or URL).*<br>• This text is appropriate if the data are owned by a third party and authors do not have permission to share the data.<br><br>* typeset	
Additional data availability information:	Tick here if the URLs/accession numbers/DOIs will be available only after acceptance of the manuscript for publication so that we can ensure their inclusion before publication.

Manuscript

Click here to access/download;Manuscript;Lund_etal_EHV_PNTDs_revised_cl

Exposure, hazard, and vulnerability all contribute to *Schistosoma haematobium* re-infection in northern Senegal

Andrea J. Lund[1*], Susanne H. Sokolow[2,3], Isabel J. Jones[2], Chelsea L. Wood[4], Sofia Ali[5], Andrew Chamberlin[2], Alioune Badara Sy[6], M. Moustapha Sam[6], Nicolas Jouanard[6,7], Anne-Marie Schacht[6,8], Simon Senghor[6], Assane Fall[6], Raphael Ndione[6], Gilles Riveau[6,8], Giulio A. De Leo[2,3], David López-Carr[9]

[1] Emmett Interdisciplinary Program in Environment and Resources, Stanford University, Stanford, California, United States of America

[2] Hopkins Marine Station, Stanford University, Pacific Grove, California, United States of America

[3] Woods Institute for the Environment, Stanford University, Stanford, California, United States of America

[4] School of Aquatic and Fishery Sciences, University of Washington, Seattle, Washington, United States of America

[5] Stanford University, Stanford, California, United States of America

[6] Centre de Recherche Biomédicale– Espoir Pour La Sante, Saint Louis, Sénégal

[7] Station d'Innovation Aquacole, Saint Louis, Sénégal

[8] University of Lille, CNRS, INSERM, CHU Lille, Institut Pasteur de Lille, Center for Infection and Immunity of Lille, Lille, France

[9] Department of Geography, University of California, Santa Barbara, CA, USA

* Corresponding author

Email: andrea.janelle.lund@gmail.com (AJL)

Abstract

Background: Infectious disease risk is driven by three interrelated components: exposure, hazard, and vulnerability. For schistosomiasis, exposure occurs through contact with water, which is often tied to daily activities. But water contact does not imply risk unless the environmental hazard of snails and parasites is also present in the water. By increasing reliance on hazardous activities and environments, socio-economic vulnerability can hinder reductions in exposure to a hazard. We aimed to quantify the contributions of exposure, hazard, and vulnerability to the presence and intensity of *Schistosoma haematobium* re-infection.

Methodology/Principal Findings: In 13 villages along the Senegal River, we collected parasitological data from 821 school-aged children, survey data from 411 households where those children resided, and ecological data from all 24 village water access sites. We fit mixed-effects logistic and negative binomial regressions with indices of exposure, hazard, and vulnerability as explanatory variables of *Schistosoma haematobium* presence and intensity, respectively, controlling for demographic variables. Using multi-model inference to calculate the relative importance of each component of risk, we found that hazard ($\Sigma w_i = 0.95$) was the most important component of *S. haematobium* presence, followed by vulnerability ($\Sigma w_i = 0.91$). Exposure ($\Sigma w_i = 1.00$) was the most important component of *S. haematobium* intensity, followed by hazard ($\Sigma w_i = 0.76$). Model averaging quantified associations between each infection outcome and indices of exposure, hazard, and vulnerability, revealing a positive association between hazard and infection presence (OR = 1.49, 95% CI 1.12, 1.97), and a positive association between exposure and infection intensity (RR 2.59 – 3.86, depending on the category; all 95% CIs above 1)

Conclusions/Significance: Our findings underscore the linkages between social (exposure and vulnerability) and environmental (hazard) processes in the acquisition and accumulation of *S. haematobium* infection. This approach highlights the importance of implementing both social and environmental interventions to complement mass drug administration.

Author Summary

While the impacts of natural hazards tend to be described in terms of social determinants such as exposure and vulnerability, the risk for infectious disease is often expressed in terms of environmental determinants without fully considering the socio-ecological processes that put people in contact with infective agents of disease. In the case of schistosomiasis, risk is determined by human interactions with freshwater environments where schistosome parasites circulate between people and aquatic snails. In this study, we quantified the relative contributions of exposure, hazard, and vulnerability to schistosome re-infection among schoolchildren in an endemic region of northern Senegal. We find that hazard and vulnerability influence whether a child becomes infected, while exposure and hazard influence the burden of worms once infection is acquired, where increasing numbers of worms lead to increasing severity of disease. Our findings underscore the importance of evaluating social and environmental determinants of disease simultaneously; omitting measures of exposure, hazard or vulnerability may limit our understanding of risk.

Introduction

When a natural hazard like a flood or hurricane is imminent, risk managers and first responders must account for the exposure and vulnerability of affected populations, conditions, and processes that are dynamic in both space and time [1]. Precious resources will be misplaced if authorities help families whose homes have not flooded, because those resources will have been diverted away from families who have been exposed to the impacts of the hazard. Similarly, vulnerable families with a reduced capacity to anticipate, respond to or recover from the impacts of a natural hazard will require more assistance than less vulnerable families [1]. An effective response to natural disasters, as a result, requires evaluation of where a natural hazard overlaps with exposure, and how vulnerability mediates exposure and its impacts in time and space.

In contrast to the risk of natural disasters, the risk of infectious disease is often defined in terms of its environmental determinants [2–4]. Risk of insect-borne diseases like Lyme disease and malaria are often described in terms of 'entomological risk,' such as the density of infected nymphal ticks (for Lyme disease) [5] or the probability of an infective mosquito bite (for malaria) [6]. Similarly, wildlife biodiversity – representing the size of the natural reservoir of potentially zoonotic pathogens – is used as a proxy for spillover risk [7]. While these measures reflect important components of pathogen transmission, their focus on the source of the disease-causing agent overlooks the socio-ecological processes that connect those infectious agents with susceptible people, thereby facilitating transmission and determining risk. Environmental metrics represent a single component of infectious disease risk: hazard [6,8]. Meanwhile, exposure and vulnerability reflect the roles of human behavior and social structures in transforming hazard into risk: exposure describes contact between a person and a pathogen (e.g., exposure to a hazard),

while vulnerability determines the extent to which people can anticipate, adapt to and potentially mitigate the impacts of infection [6,9].

These three components of risk – exposure, hazard, and vulnerability – operate in concert rather than isolation. They comprise the breadth of socio-ecological conditions that put and keep people in contact with infectious agents of disease. Few studies address all three components simultaneously, but those that do show how human interaction with the environment transforms the presence of a hazard into risk for infection and disease. For example, accounting for both entomological risk (i.e., hazard) and exposure explained Lyme disease incidence in New York when entomological risk alone did not [5]. In East Africa, incorporating social vulnerability and entomological risk into a spatially-explicit risk assessment of malaria showed how different interventions could be targeted to different places with different needs [10,11]. Across disease systems, insights about the relative contributions of exposure, hazard and vulnerability can inform which interventions to employ to mitigate risk and where, when, and how to implement them.

The risk for schistosomiasis – a disease caused by parasitic worms from the genus *Schistosoma* – can also be divided into these three distinct components. Schistosomes complete a two-host transmission cycle between humans and specific species of freshwater snail[illegible] People become infected through contact with freshwater where schistosomes' free-living infective larval stage (called cercariae) emerge from infected snails and burrow into human skin. Schistosome hazard is often described as 'malacological risk,' and quantified using ecological indicators of snail populations within a water source [12–14]. Similarly, exposure to schistosomes is often approximated by studying human water contact behavior because the process of cercariae burrowing into skin upon contact cannot be directly observed [15–17]. Even so, exposure to

surface water on its own does not imply risk for infection [18,19]; the hazard of infected snails in a water source, and the circulation of parasites through the snail population is what renders water contact harmful [6]. Lastly, vulnerability determines whether a person can avoid or mitigate the schistosome hazard in their surroundings, either by reducing exposure or contamination of water bodies [20,21], or seeking treatment for infection [22]. The relative disadvantage of certain groups may make risk mitigation difficult, obliging people to remain in contact with parasite-laden water even if they are aware of the risk [6].

Approximately 200 million people are infected with schistosomes worldwide, making schistosomiasis second only to malaria in the global burden of parasitic disease [23,24]. Schistosome infections are treatable with the antiparasitic drug praziquantel. Since the 1980s, mass drug administration (MDA) campaigns employing praziquantel have been the central strategy of schistosomiasis control, displacing the snail control interventions of the preceding decades [25,26]. These MDA programs have played an important role in reducing morbidity due to schistosomiasis, but because praziquantel does not prevent new infections, the benefits of MDA have not been realized universally across all contexts [27]. The identification of persistent hot spots – or settings whose prevalence and intensity of infection remains high despite repeated rounds of treatment – has underscored the need for interventions that control transmission and complement the morbidity control achieved by MDA [28–30]. Controlling transmission will require both ecological and social points of intervention, whose identification and implementation can be informed by understanding the relative contributions of hazard, exposure, and vulnerability in the risk for acquiring and accumulating schistosome infections.

In this mixed methods study, we combined parasitological, ecological, and socio-economic data from 16 villages in the lower basin of the Senegal River to better understand the

relative contribution of exposure, hazard, and vulnerability to the burden of urogenital schistosomiasis in an endemic region of northern Senegal. In this setting, the construction of a dam and irrigation infrastructure has reduced the region's susceptibility to drought and famine, while simultaneously facilitating schistosome transmission. Prior to dam construction, the transmission of *S. haematobium* occurred at low levels on a seasonal basis, but the disease has since become hyperendemic with perennially high prevalence and intensity of infection [31–33]. Dam construction also coincided with the introduction and rapid spread of *S. mansoni,* an intestinal schistosome, which remains co-endemic with *S. haematobium* [34,35]. Simultaneously, agricultural livelihoods have shifted from traditional flood recession and rainfed cultivation to more intensively irrigated crops [36]. The social and environmental changes in this setting have collectively affected exposure, hazard, and vulnerability to schistosome infection. We compared the relative contributions of exposure, hazard and vulnerability as explanatory variables of both presence and intensity of *S. haematobium* infection. We then quantified the magnitude and precision of the association between infection outcomes and indices of each component of risk as well as demographic characteristics, whose importance has been demonstrated in this and other settings [37–39]. Using a mix of survey, interview, and ecological data to assess the relative contributions of exposure, hazard, and vulnerability to schistosomiasis risk, we identify the circumstances under which ecological, infrastructural, and behavioral interventions can complement mass drug administration in campaigns to control and eliminate schistosomiasis.

Methods

Study area and population

This study used cross-sectional, mixed methods data collected in 2017 and 2018 as part of a longitudinal study of schistosome infection in school-aged (5-15 years) children, including

parasitological examinations of urine samples screening for *S. haematobium* infections, survey data from the residents and heads of households where those children reside, and interview and ecological data from water access sites in their corresponding villages. Sixteen villages along the lower Senegal River, its tributaries, and the Lac de Guiers in northwest Senegal were selected for participation in the longitudinal study; these villages were representative of the rural, high-transmission sites common in the region (see appendix of [33] for details on village selection; Fig. 1). Briefly, the criteria used to select villages included proximity to freshwater, presence of water access sites, presence of a school with sufficient enrollment in target primary school grades, a non-zero prevalence of self-reported infection, and accessibility in the rainy season [33]. Of these 16 study villages, six were located along the Senegal River and its tributaries while ten were located on the shores of Lac de Guiers. Three of these 16 villages (one on the river and two on the lake) were excluded from this analysis because a vegetation removal intervention took place in these sites in 2017, potentially influencing hazard indices (Figure 1)

Figure 1. The lower basin of the Senegal River, where parasitological, socio-economic, and ecological data were collected from 821 school-aged children in 411 households and 24 water access points in 13 villages. Three villages (shown in white circles) were excluded from this analysis because they were sites of a vegetation removal intervention in 2017 that may have affected hazard indices.

In each village, school-aged children were recruited from grades 1–3 in village schools, and each provided urine samples for determining *S. haematobium* infection presence and intensity in January–March 2018. In July 2018, we attempted to reach all the households where students enrolled in the school-based parasitology study lived and administer a questionnaire about household demographics and water contact behavior. This study received approval from the National Committee of Ethics for Health Research from the Republic of Senegal (Protocol #SEN14/33) as well as the Institutional Review Boards of the University of California, Santa

Barbara (Protocol #19-17-0676) and Stanford University (Protocol #43130). Children were enrolled in the parasitology study after providing assent alongside written informed consent from the child's parent or guardian. All interview and survey respondents provided verbal informed consent.

Data collection

Parasitology data collection. Through schools in all 16 villages, a total of 1,480 school-aged children was enrolled at baseline in February–April 2016. Of those, 1,301 remained enrolled during the third year of follow-up in January–March 2018 and successfully produced urine samples on each of two testing days. After excluding children living in the three villages where the vegetation removal intervention occurred in 2017, parasitological data were available for 821 children in 13 villages (Fig. S1). At baseline and the subsequent two years, urine samples were analyzed by urine filtration for *S. haematobium* infection [40]. Following sample collection, all children were treated at school (with parental consent) with two rounds of praziquantel administration at 40 mg/kg. Because the cross-sectional data used in this study were embedded in a large longitudinal study in the same villages, infection data collected in January–March 2018, which were used as outcome variables in this study, reflect re-infection following treatment over the course of the preceding year [41–43].

Household survey data collection. Household-level survey data were collected at the beginning of the rainy season in July 2018, following parasitological data collection for that year. In all 16 villages, we aimed to reach all households with children enrolled in the school-based parasitology study. Of the 1,301 students enrolled in the parasitology study, we reached the households (n = 524) of 1,216 students (93.5%; Fig. S1). The household survey instrument included six main modules (S1 Table). The modules used in this analysis included: (1)

individual-level demographic information, (2) individual-level, two-week recall of contact with surface water for six primary domestic and occupational chores requiring contact with water, (3) average frequency of each water contact activity reported at the household level, (4) socio-economic indicators, such as reported access to improved water and sanitation infrastructure and (5) geographic coordinates of household locations. The collection of water contact data in the survey was designed to elicit information on water contact behavior over the course of a typical week.

Body surface area interview data collection. In all but one village, brief interviews were conducted with village residents, usually at village water access sites in August 2016. In each interview, respondents were asked how much of the body typically comes into contact with water for each of six common water contact activities: (1) washing laundry, (2) washing dishes, (3) collecting water, (4) irrigating fields, (5) washing or watering livestock and (6) fishing from shore [39]. Responses for each activity were registered on a diagram used to measure burn size [44]. Two interviews were conducted in each village – one with an adult man and one with an adult woman – for a total of 30 interviews and 210 activity-specific observations (30 interviews x 7 water contact activities).

Ecological data collection. Snail surveys were performed at 32 water access sites in all 16 study villages, the details of which are described elsewhere [33]. Briefly, randomized snail sampling was stratified by three microhabitat types (emergent vegetation, non-emergent vegetation, and open water/mud bottom), such that sampling effort within a site was proportionate to the relative area of each microhabitat. Imagery from Google Earth and an unmanned aerial vehicle (drone) was used to identify site boundaries and designate fifteen random sampling points within each microhabitat. At each point, exhaustive sampling for snails occurred within a quadrat (76.2 cm x

48.26 cm x 48.26 cm; area = 0.3677 square meters). The snails collected from each quadrat were place into labeled vials and returned to the laboratory for identification and infection screening via shedding and dissection assays. Molecular identification of schistosome cercariae shed from captured snails in the laboratory distinguished human-infecting schistosome species from species that affect non-human animals. Data from the 24 water access sites that were not part of the vegetation removal intervention were included in the analysis.

Data analysis

Infection metrics. Schistosome infections were determined to be present when any of the samples collected from an individual student contained at least one schistosome egg. Infection intensity was quantified by taking the median egg count (per 10 mL urine) across the samples collected at both parasitological sampling visits. Taking the median of the value(s) kept our estimates conservative, because they were not skewed by missing or zero values.

Exposure indices. Exposure indices were calculated using data from three sources: (1) individual- and household-level survey data, (2) body surface area interviews and (3) a previous study of water contact behavior in a similar setting [39]. These indices aggregated reported water contact across the six water contact activities described above, including in the body surface area interview form and the water contact module in the household survey. Whether or not a person performed a given activity in the preceding two weeks was recorded as binary individual-level variables in the household survey. The average frequency of each activity was reported at the household level in the survey, while duration and extent of contact were drawn from literature-based estimates and body surface area interviews, respectively (Table S2). Exposure indices based on these activity-specific data incorporated varying degrees of complexity, including

weekly frequency-only estimates, weekly time exposed and weekly time exposed adjusted for mean body surface area exposure.

Two indices measured frequency of contact with water. One index [*F (raw)*] was a categorical variable derived directly from a single survey question, where each child was asked to recall the number of visits made to a water access point in the preceding seven days. The second [*F (sum)*] was a numeric variable derived from activity-specific measures collected through the survey at the individual and household levels. Individual-level water contact data indicated whether each member of the household had performed each activity in surface water (e.g., lake, river, irrigation canals) in the preceding two weeks. Dichotomous responses were registered for all members of the household. Response categories for frequency of each water contact activity were collected at the household level (e.g., one value per household) and converted to numeric values representing times per week (e.g., a reported frequency of once per day was converted to a numeric value of 7 times per week). The numeric values of household-level activity frequency were multiplied by indicators of individual-level activity to generate estimated weekly contacts by activity for all children in the data set:

$$F(sum) = \sum_{activity}(activity\ performed) * (frequency) \qquad (1)$$

Total time exposed per week (*FD*) was calculated by multiplying the weekly frequency of an activity by the estimated duration of that activity, as calculated from direct observations in another study in a similar setting [39]. The product of frequency and duration was then summed across all activities for each child to generate an estimated total time (in minutes) of contact with water over the course of a week.

$$FD = \sum_{activity}(activity\ performed) * (frequency) * (duration) \qquad (2)$$

Total exposure time per week was then adjusted by estimated body surface area in contact with water for a given activity (FDB). We used data from body surface area interviews (Table S2) to estimate how much of the body came into contact with water for each activity. For each observation, the percent of body surface area (%BSA) was summed for each body segment reported as exposed for that activity using published values for burn size estimation [44]. Mean %BSA for each activity was calculated across all interview respondents. Activity-specific estimates of body surface area exposed (in square meters) were calculated by multiplying mean %BSA by an estimated total body surface area for children under 14 years of age [45]. Estimated body surface area exposure required for each activity was multiplied by the total time exposed for that activity, giving a BSA-adjusted time per week.

$$FDB = \sum_{activity}(activity\ performed) * (frequency) * (duration) * (body\ surface) \quad (3)$$

Hazard indices. Hazard indices were calculated from ecological field data collected at the 24 water access points in the same 13 villages where parasitology and household survey data were collected. A previous study in this setting found that area of suitable snail habitat (e.g., area covered by non-emergent aquatic vegetation) was the best environmental predictor of presence and intensity of *S. haematobium* infection [33]. We developed six hazard indices based on area of snail habitat, which varied by (1) season, (2) spatial relationship and (3) level of data aggregation. Two hazard variables captured the area of suitable snail habitat (1) for the peak transmission season preceding parasitological data collection for each water access point (July – August 2017; peak) and (2) summed across the three ecological data collection periods in the year preceding parasitological data collection (May–June 2017, July–August 2017 and January 2018; year). Summing habitat area across the year represents an integrated hazard metric across the three dominant climatic seasons in the region. We primarily used data from the water access

site nearest to each household, based on straight-line distance, but two additional hazard variables captured the spatial relationship between households and the nearest water access sites by scaling *peak* and *year* hazard indices by the distance between a child's household and the water access site. Two final hazard variables aggregated *peak* and *year* indices to the village level, where the area of suitable snail habitat during the transmission peak and the entire year were summed across all water points in a village.

Vulnerability indices. Vulnerability indices were calculated from the household survey data and focused on access (or lack thereof) to water and sanitation infrastructure at the household level (S1 Table). Two survey questions concerned water sources: (1) what is the principal source of water used by members of the household for drinking? And (2) what is the principal source of water used for doing laundry? Responses to these two questions were used to calculate the extent to which each household depended on surface water for household needs (e.g., surface water is used for neither drinking water nor laundry; surface water is used for either drinking water or laundry; or, surface water is used for both drinking water and laundry). This variable was also dichotomized (e.g., a household used surface water for any activity versus no activities).

Two additional survey questions concerned sanitation infrastructure: (1) what type of toilet is primarily used by members of this household? and (2) is this toilet shared with other households? Responses to these two questions were used to calculate a three-level variable describing sanitation access (e.g., private toilet, shared toilet, no toilet). A dichotomous version of this variable was also calculated (e.g., private toilet versus shared or no toilet). This dichotomous categorization was chosen because few households reported having no toilet.

A final index of vulnerability was an index of socio-economic status (SES) derived from data on durable assets and household conditions reported in the household survey [46,47].

Principal components analysis was performed using the *prcomp()* function in the *stats* package (version 3.5.1) in R [48]. Loadings for individual variables in the first principal component were used to calculate a numeric socio-economic score for each household. Numeric scores were then divided into quintiles.

Statistical analysis. We used mixed-effects logistic and negative binomial regression models to assess exposure, hazard, and vulnerability indices as explanatory variables of schistosome infection presence (i.e., infected versus uninfected) and intensity (i.e., number of eggs detected per 10mL urine), respectively. In all models, we controlled for key social and demographic covariates: age and sex of the child, village location (i.e., river versus lake) and population size. We also included random intercepts (households nested within villages) to account for the hierarchy in the data. Mixed effects logistic regression was performed using the *lme4* package (version 1.1-21) [49], while mixed effects negative binomial regression was performed using the *glmmTMB* package (version 1.0.1) [50] in R (version 3.5.1) [48].

We used information-criterion (IC)-based model selection and multi-model inference to determine the relative importance of each component of risk [51]. For each outcome, we first fit a set of exposure-only, hazard-only, and vulnerability-only models. The Akaike Information Criterion (AIC) was used to discern the best-fitting index (or indices) of each component of risk. We retained all variables in exposure-only, hazard-only and vulnerability-only models whose ΔAIC was less than 2 across the models in a given group [51]. This subset of variables was then used to fit models that included various combinations of the three components of risk: (1) exposure and hazard (E-H), (2) exposure and vulnerability (E-V), (3) hazard and vulnerability (H-V) and (4) all three: exposure, hazard, and vulnerability (E-H-V). Because fewer indices of exposure, hazard and vulnerability were used in these combination models of infection presence

(based on AIC for exposure-, hazard-, and vulnerability- only models), we fit a set of 28 candidate models for *S. haematobium* presence and 47 candidate models for *S. haematobium* re-infection intensity. Regression diagnostics indicated no problem with multicollinearity in models combining indices of exposure, hazard, and vulnerability.

For both sets of models, we calculated AIC, ΔAIC and Akaike weights (w_i) [51]. The sum of Akaike weights across the exposure, hazard and vulnerability components of risk was used to quantitatively assess their relative importance across the full set of candidate models for both presence and intensity outcomes [51]. Additionally, for each index of exposure, hazard and vulnerability, we calculated model-averaged estimates of the magnitude and precision of the association with both infection outcomes using the *AICcmodavg* package in R [52]. Odds ratios (OR) and rate ratios (RR) with 95% confidence intervals (95% CIs) were estimated for presence and intensity outcomes, respectively.

Results

Characteristics of the study population

Of the 821 school-aged children with complete information across the parasitological, survey and ecological data sets, the mean age was 9.3 years and approximately half (414/821; 50.4%) were male (Table 1). On average, these children lived within 521 meters of a water access point on the Senegal River, a tributary, or the Lac de Guiers, which contained between zero and 149,775 square meters of suitable snail habitat in the peak transmission season. Approximately half of the households where these children lived relied on surface water collected from water access points for at least one household need, and most households (82.6%) reported primarily using private sanitation infrastructure (Table 1).

Table 1. Demographic characteristics of the overall study population, and stratified by sex

Variable	Overall	Males	Females
Observations [n]	821	414	407
Age (years) [mean (SD)]	9.3 (3.1)	9.2 (2.2)	9.3 (3.8)
Distance to water point (m) [mean (SD)]	521.6 (505.3)	522.2 (530.6)	521.0 (475.5)
Water point habitat area (m^2) [mean (range)]	38,857 (0 - 149775)	38,898 (0 – 100,361)	38,815 (0 – 149,775)
Surface water dependence [n (%)]			
Neither drinking nor laundry	400 (48.7)	211 (51.0)	189 (46.4)
Either drinking or laundry	265 (32.3)	129 (31.2)	136 (33.4)
Both drinking and laundry	156 (19.0)	74 (17.9)	82 (20.1)
Sanitation facility [n (%)]			
Private toilet	679 (82.7)	342 (82.6)	337 (82.8)
Shared toilet	134 (16.3)	68 (16.4)	66 (16.2)
No toilet	8 (0.9)	4 (0.9)	4 (1.0)
Asset-based wealth index [n (%)]			
1st quintile (Lowest)	133 (16.2)	76 (18.4)	57 (14.5)
2nd quintile	116 (14.1)	57 (13.7)	59 (14.5)
3rd quintile	150 (18.3)	82 (19.8)	68 (16.7)
4th quintile	201 (24.5)	100 (24.2)	101 (24.8)
5th quintile (highest)	221 (26.9)	99 (23.9)	122 (30.0)

The burden of *S. haematobium* among this study population was high. The overall prevalence was 66.7% and was slightly higher among boys (72.9%) than girls (60.4%) (Table 2). The geometric mean of *S. haematobium* infection intensity was 4.9 eggs per 10 mL urine (SD = 117.4) overall, 6.4 eggs per 10 mL urine (SD = 66.8) for boys and 3.7 eggs per 10 mL urine (SD = 152.0) for girls (Table 2).

Table 2. Descriptive analysis of outcome variables (presence and intensity of *S. haematobium* infection) in the overall study population and stratified by sex

Variable	Overall	Males	Females
Observations [n]	821	414	407
Infection presence [infected n (%)]	548 (66.7)	302 (72.9)	246 (60.4)
Infection intensity [GM (SD)]	4.9 (117.4)	6.4 (66.8)	3.7 (152.0)

GM = geometric mean eggs/10mL urine

When asked about contact with surface water generally, over one-third (39.1%) of school-aged children reported no visits to village water access sites in the preceding seven days. Approximately one-quarter (26.7%) visited once or twice a week and the remainder (34.3%)

reported more frequent visits (Table 3). When prompted to recall activity-specific contact with surface water (e.g., doing laundry or washing livestock), the average sum of total weekly contacts across all activities was much higher (mean = 8.5 times per week, SD = 10.9). The weekly frequency of some of these activities (e.g., bathing and dish washing) exceeded that reported from the single survey question prompting seven-day recall of all visits to the water access point (Table 3).

Table 3. Descriptive analysis of exposure indices calculated from individual-level responses to water contact questions in the household survey, overall and stratified by sex

Variable	Overall	Males	Females
Categorical frequency [n (%)]			
No visits per week	321 (39.1)	145 (33.0)	176 (43.2)
1-2 visits per week	219 (26.7)	121 (29.2)	98 (24.1)
3-6 visits per week	68 (8.3)	33 (8.0)	35 (8.6)
7 visits per week	173 (21.1)	95 (22.9)	78 (19.2)
7+ visits per week	40 (4.9)	20 (4.8)	20 (4.9)
Weekly activity-specific frequency [mean (SD)]			
Bathing	4.7 (5.2)	4.6 (5.1)	4.8 (5.3)
Water collection	1.4 (3.4)	1.0 (2.9)	1.8 (3.9)
Dishes	1.2 (3.3)	0.1 (1.0)	2.2 (4.3)
Laundry	0.7 (1.9)	0.1 (0.7)	1.3 (2.5)
Livestock	0.3 (1.5)	0.5 (1.9)	0.1 (0.9)
Irrigation	0.2 (1.2)	0.2 (1.2)	0.1 (1.3)
Fishing	0.1 (0.8)	0.1 (0.8)	0.03 (0.7)
Numeric frequency [mean (SD)]	8.5 (10.9)	6.6 (7.5)	10.4 (13.2)
Total weekly time exposed [mean (SD)]	87.3 (111.4)	66.0 (74.6)	109.0 (135.9)
BSA-adjusted weekly time exposed [mean (SD)]	74.5 (83.4)	65.6 (71.7)	83.5 (93.0)

Bathing was assumed to involve contact with the entire body surface, while shore fishing (47.6%) and water collection (45.3%) involved the next greatest body surface area in contact with water (S2 Table). Irrigation (22.5%) was the activity with the least body surface area exposed (S2 Table). Using the reported duration of activity-specific water contact, we estimated that the school-aged children in this setting spend, on average, 87.3 minutes (SD = 111.4) in contact with surface water per week, an estimate that is higher for girls (mean = 109.0 minutes, SD = 135.9) than boys (mean = 66.0 minutes, SD = 74.6). Adjusting weekly total duration of

contact by total body surface exposed during a specific activity reduced this overall estimate slightly to 74.5 m^2-minutes, with similar variation between boys (mean = 65.6 m^2-minutes, SD = 71.7) and girls (mean = 83.5 m^2-minutes, SD = 93.0).

While survey data indicate that dependence on surface water is not evenly distributed across the quintiles of the asset-based SES index (Kruskal-Wallis test, X^2 = 14.0, df = 4, P-value = 0.007), we find that those who do depend on surface water for at least one household task are relatively evenly distributed across SES quintiles (S3 Table). For example, almost a third of the households (29.8%) that use surface water for either drinking or laundry are in the highest SES quintile. We find a similar pattern for dependence on surface water for both drinking and laundry: almost a quarter of households (21.8%) in the highest SES quintile rely on surface water for both chores, which is identical to the proportion of households in the lowest SES quintile with the same reliance on surface water (S3 Table).

Best-fitting indices of exposure, hazard, and vulnerability

Four exposure indices, six hazard indices, and five vulnerability indices were considered in a set of mixed-effects logistic regression models of *S. haematobium* infection presence (n = 28) and a set of mixed-effects negative binomial regression models of *S. haematobium* infection intensity (n = 47; Table 4). The full comparison of model fit, including the Akaike weight (w_i) for each individual model, is summarized in S4 Table for presence models and in S5 Table for intensity models. Data in Table 4 summarize each variable used to approximate exposure, hazard, or vulnerability as well as the number of models that variable appeared in for each outcome. For infection presence, all four exposure-only models were within a ΔAIC of 2. For infection intensity, the categorical frequency of water point visits (*F (raw)*) outperformed all three numeric indices of exposure (numeric frequency [*F (sum)*], total time exposed [*FD*] and

BSA-adjusted time exposed [FDB]*)*. The exposure-only model of infection intensity containing the *F (raw)* index had the lowest AIC of the four exposure-only models, with the ΔAIC for the next best-fitting model of infection intensity far exceeding the threshold of 2 (ΔAIC = 16.52; S5 Table). As a result, we retained all four indices of exposure for models of infection presence, each of which appeared in a total of four of the 28 (14.2%) presence models. In contrast, the categorical frequency of water access site visits was the only index of exposure retained in subsequent models of infection intensity, appearing in a total of 20 of 47 (43.5%) models in that set (Table 4).

Table 4. Summary of the indices of exposure, hazard and vulnerability that were compared in a set of 28 mixed effects logistic regression models of *S. haematobium* infection presence and a set of 47 mixed effects negative binomial regression models of *S. haematobium* infection intensity and the number (%) of models in each set in which each variable appeared

	Variable	Description	Models (n (%)) Presence	Intensity
Exposure	F (raw)	Categorical frequency of visits to water access point in previous seven days, derived from single survey question	4 (14.2)	20 (43.5)
	F (sum)	Numeric frequency of weekly contacts with water, based on individual-level binary indicators for seven common water contact activities and average household-level frequencies, both of which were collected through the household survey	4 (14.2)	1 (2.2)
	FD	Weekly time exposed (in minutes) derived from activity-specific survey data, as in *F (sum)*, combined with literature-derived data on duration of exposure [39]	4 (14.2)	1 (2.2)
	FDB	Weekly time exposed adjusted for mean body surface area exposed for each of seven common water contact activities, data estimated from body surface area interviews	4 (14.2)	1 (2.2)
Hazard	areaPeak	Area of snail habitat at the water access site nearest to household during peak season	1 (3.6)	8 (17.4)
	areaYear	Area of snail habitat at the water access site nearest to household summed over year	1 (3.6)	8 (17.4)
	areaPeak_d	Area of snail habitat at the water access site nearest to household during the peak season, adjusted by the distance between the site and the household	1 (3.6)	8 (17.4)
	areaYear_d	Area of snail habitat at the water access site nearest to child's household summed over year, adjusted by the distance between the site and the household	10 (35.7)	8 (17.4)
	areaPeakV	Area of snail habitat summed across all water points in a village during the peak season	1 (3.6)	1 (2.2)
	areaYearV	Area of snail habitat summed across all water points in a village and across year	1 (3.6)	1 (2.2)
Vulnerability	surface	Dependence on surface water for household needs derived from survey data: (0) neither laundry or drinking, (1) either laundry or drinking or (2) both laundry and drinking	10 (35.7)	1 (2.2)
	surfaceYN	Dichotomous version of surface water dependence for any versus no activities	1 (3.6)	10 (21.7)
	sanitation	Primary sanitation infrastructure used by members of a household: (0) none, (1) shared toilet, (2) private toilet	1 (3.6)	10 (21.7)
	privateSan	Dichotomous version of sanitation, where members of a household use either a private toilet or shared/no toilet	1 (14.2)	10 (21.7)
	assetIndex	Quintiles of an asset-based SES index created using principal components analysis	1 (3.6)	1 (2.2)

A single index of hazard – the area of snail habitat at the water access site nearest to a child's household summed over the three climatic seasons of ecological data collection, adjusted by the distance between the site and the household (*areaYear_d)* – had a ΔAIC ≤ 2 compared to other hazard-only models of *S. haematobium* presence (S4 Table). As a result, *areaYear_d* was the only index of hazard to appear in more than one model of *S. haematobium* presence (Table 4). In contrast, five indices of hazard had a ΔAIC ≤ 2 compared to other hazard-only models of *S. haematobium* intensity (S5 Table). These variables, which included the area of snail habitat at the water access site nearest to a child's household during the peak transmission season (*areaPeak*) as well as area summed over the entire year (*areaYear*). The distance-adjusted analogs of these variables (*areaPeak_d* and *areaYear_d*) were also retained as was the area of snail habitat summed across all water points in a village and across the full year (*areaYearV*). These five indices of hazard were retained in subsequent models that combined hazard with other components of risk, such that all five appear in multiple models of *S. haematobium* intensity (Table 4).

One index of vulnerability – categorical dependence on surface water (*surface*) – best fit the *S. haematobium* presence data (e.g., ΔAIC ≤ 2 among vulnerability-only models; S4 Table). This variable was retained for subsequent models that combined components of risk and appeared in ten (35.7%) of the 28 presence models (Table 4). In contrast, binary dependence on surface water (*surfaceYN*) as well as categorical and binary variables for sanitation infrastructure (*sanitation* and *privateSan*, respectively) all had ΔAIC ≤ 2 among vulnerability-only models of *S.haematobium* intensity (S4 Table). Each appeared in ten (21.7%) of the 47 intensity models (Table 4).

Relative importance of exposure, hazard, and vulnerability

Across these sets of candidate models, no single model emerged as the best-fitting model of *S. ha*[illegible]*tobium* re-infection presence or intensity (S4 and S5 Tables). The largest w_i for mixed effects logistic regression models of *S. haematobium* presence was 0.28. A total of five models were classified as having substantial support (ΔAIC < 2) [51]. Four of these five models included all three components of risk while the fifth (and best-fitting model) contained indices of hazard and vulnerability (Table 5)

Table 5. Inclusion of indices of exposure (E), hazard (H) and vulnerability (V) in models of *S. haematobium* presence and intensity models with substantial support (ΔAIC <2)

**No.**	**E**	**H**	**V**	**AIC**	**ΔAIC**	**w_i**	**Σw_i**
Presence models with substantial support (from S4 Table)							
24		X	X	837.37	0	0.28	0.28
26	X	X	X	838.32	0.96	0.18	0.46
27	X	X	X	838.57	1.21	0.16	0.62
28	X	X	X	838.89	1.52	0.13	0.75
25	X	X	X	839.21	1.84	0.11	0.86
Intensity models with substantial support (from S5 Table)							
1	X			5494.29	0	0.08	0.08
16	X	X		5494.41	0.12	0.08	0.15
19	X	X		5494.46	0.17	0.08	0.22
42	X	X	X	5494.46	0.17	0.07	0.30
22	X		X	5494.49	0.20	0.07	0.37
18	X	X		5494.86	0.57	0.06	0.42
43	X	X	X	5494.86	0.57	0.06	0.48
40	X	X	X	5494.95	0.66	0.06	0.54
36	X	X	X	5494.98	0.68	0.06	0.59
21	X		X	5495.21	0.92	0.05	0.64
17	X	X		5495.30	1.01	0.05	0.69
20	X	X		5495.50	1.21	0.04	0.73
41	X	X	X	5495.70	1.41	0.04	0.77
38	X	X	X	5495.81	1.52	0.04	0.81
37	X	X	X	5495.95	1.66	0.03	0.84
39	X	X	X	5496.16	1.87	0.03	0.87
46	X	X	X	5496.19	1.90	0.03	0.90
23	X		X	5496.23	1.94	0.03	0.93

X indicates whether a variable from a given component of risk was included in the model; AIC = Akaike information criterion; w_i = Akaike weight; Σw_i = sum of Akaike weights across models in a set

The largest w_i for mixed-effects negative binomial regression models of *S. haematobium* intensity was 0.08, with 18 models classified as having substantial weight [51]. Half (9 /18) of these models included indices of all three components of risk. An index of exposure was included in all the best models of re-infection intensity, including an exposure-only model which had the lowest AIC of all models of re-infection intensity (Table 5).

We used the sum of Akaike weights (Σw_i) across each set of candidate models for each outcome to quantify the relative importance of each component of risk (Table 6). We found that hazard was the most important component of risk for re-infection presence in school-aged children ($\Sigma w_i = 0.95$). The relative importance of hazard for *S. haematobium* presence was followed by vulnerability ($\Sigma w_i = 0.91$) and then exposure ($\Sigma w_i = 0.66$; Table 6). The relative importance of each component of risk differed for models of *S. haematobium* re-infection intensity, where exposure (measured as categorical frequency of contact with surface water) was the most important metric ($\Sigma w_i = 1.00$), followed by hazard ($\Sigma w_i = 0.76$) and then vulnerability ($\Sigma w_i = 0.66$).

Table 6. Sum of Akaike weights (Σw_i) as a measure of relative importance of the three components of risk for *S. haematobium* infection presence and intensity

		Σw_i	
	Exposure	Hazard	Vulnerability
Infection presence	0.66	0.95	0.91
Infection intensity	1.00	0.76	0.66

Model-averaged estimates

We used model averaging to calculate the magnitude and precision of the association between both infection outcomes and all indices of exposure, hazard and vulnerability that appeared in more than one model across the full set of candidate models and the demographic control variables used in each model (Fig. 2). For the infection presence outcome, we found that

the odds of infection increased with distance-adjusted area of snail habitat at the nearest water point to a child's household summed over the year preceding infection data collection. The association corresponds with a 45% increase in the odds of infection with each square meter of snail habitat over the course of an entire year, with a confidence interval contained entirely above the null value of 1 (*areaYear_d*; OR = 1.49, 95% CI 1.12, 1.97).

Figure 2. Model-averaged point estimates and 95% confidence intervals for association between infection outcomes and each component of risk. Top: Associations between *S. haematobium* infection presence and components of risk are estimated by logistic regression and measured with odds ratios. Bottom: Association between *S. haematobium* infection intensity and components of risk are estimated by negative binomial regression and measured with rate ratios. In both panels, indices of exposure (E) are represented by black triangles, indices of hazard (H) by turquoise circles, indices of vulnerability (V) by blue squares and demographic control variables (D) by orange diamonds.

Associations between infection presence and indices of vulnerability were less precise, with many confidence intervals including the null value of 1 (Fig. 2). When comparing children whose households relied on surface water for multiple household tasks to those who relied on surface water for no household tasks, we found an almost nine-fold increase in the odds of infection with a confidence interval entirely above the null value of 1 (*Categorical surface water use* [2 activities vs 0]; OR = 8.67, 95% CI 1.67, 45.60). A similar association comparing households' use of surface water for a single activity was also positive, but its confidence interval crossed the null (*Categorical surface water use* [1 activity vs 0]; OR = 1.34, 95% CI 0.84, 2.14).

All levels of the exposure index suggest a positive association with infection presence (categorical frequency [*F (raw)*]), but only one level (*Categorical frequency* [3-6 times/week vs none]) had a confidence interval that did not include the null value of 1 (OR = 2.18, 95% CI 1.03, 4.66). Remaining levels of categorical exposure (1-2 times/week vs none, 7 times/week vs none and >7 times/week versus none) cannot be considered statistically different from the null

(Fig. 2). All three numeric indices of exposure had point estimates slightly below 1 (e.g., 0.9) with confidence intervals that included in the null value (Fig. 2).

Two of the four demographic control variables had significant associations with infection presence (Fig. 2). Children living in lake villages were five times more likely to be infected compared to children living in river villages (*Village location*; OR = 5.37, 95% CI 1.14, 25.28). The odds of infection were 54% lower among girls compared to boys (*Child sex*; OR = 0.46, 95% CI 0.33, 0.63) and younger children were less likely to be infected than older children (*Child age*; OR = 0.78, 95% CI 0.63, 0.96). Village population size was not associated with infection presence (*Village population*; OR = 1.16, 95% CI 0.64, 2.10).

For the infection intensity outcome, we found that the number of eggs in a 10 mL urine sample increased for all levels of exposure (*Categorical frequency* [*F (raw)*]; Fig. 2). These point estimates ranged from a 2.59- to 3.86-fold increase in egg burden among children reporting any exposure compared to those reporting no exposure. Confidence intervals for all estimates were entirely above the null value of 1 (95% CIs: [*1-2x/week vs none*] 1.52, 4.44, [*3-6x/week vs none*] 1.88, 8.00, [*7x/week vs none*] 1.55, 5.70, [*>7x/week vs none* 1.22, 7.77; Fig. 2). The directionality of the associations between infection intensity and indices of vulnerability are like those for infection presence (e.g., above 1 for surface water use, below 1 for sanitation infrastructure), but with point estimates closer to the null and wider confidence intervals that include the null value of 1 (Fig. 2).

The measures of association between infection intensity and hazard are mixed: distance-scaled and village-level indices have point estimates greater than 1 while raw values of habitat area (i.e., not scaled by distance) for the nearest water point to a child's home are less than 1. The confidence intervals for all associations between *S. haematobium* infection intensity and

hazard indices include the null value of 1. Associations between infection intensity and demographic control variables were similar to the associations described for these variables with presence outcomes (Fig. 2).

Discussion

We found that components of risk describing exposure, hazard, and vulnerability all had some importance in explaining the occurrence of *S. haematobium* in school-aged children in this endemic setting in the lower Senegal River Basin. Based on the sums of Akaike weights calculated across both sets of models, however, hazard and vulnerability were the most important components of *S. haematobium* infection presence, while exposure and hazard were the most important components of *S. haematobium* infection intensity. These differences highlight the distinct factors that influence whether someone becomes infected versus whether they accumulate a large worm burden, with implications for the design of schistosomiasis control and elimination programs and guidelines.

The importance of hazard and vulnerability to the infection presence outcome reflects the conditions that influence whether a person acquires an infection or not. Any exposure may lead to infection, making additional exposure irrelevant to this binary outcome once a person becomes infected. But hazard in the environment, or the number of infected snails emitting cercariae from a certain amount of available habitat, is more likely to increase the risk of infection so long as some exposure is happening. Vulnerability through the household activities that involve regular water contact, like dependence on surface water, for example, ensures some baseline level of exposure.

Similarly, the importance of exposure and hazard to infection intensity reflects the conditions that influence the accumulation of worms. The intensity of infection is theoretically proportional to exposure, with each new adult worm the result of a separate infection event [53]. While this has not always borne out empirically [39,54], our data agree with the theoretical expectation that more exposure leads to higher intensity infection. Such accumulation of worms can lead to severe morbidity. A study in Uganda found the prevalence of liver fibrosis to be five times greater in people who had lived in the *S. mansoni*-endemic area since birth compared to those with just a 10-year residency, leading the authors to conclude that the duration of exposure over time is an important risk factor for developing the severe morbidity resulting from prolonged high-intensity infections [55]. Another study in China found that water contact itself was not associated with infection intensity but, similar to our findings, accounting for both water contact and a measure of cercarial risk did [19].

While the recognition that schistosomiasis risk is determined by the convergence of social (exposure and vulnerability) and environmental factors (hazard) is not new [56–59], few studies comprehensively address all three components of risk for schistosomiasis specifically or for infectious diseases generally. Studies employing mathematical models have jointly addressed social and environmental determinants of schistosomiasis [60–62], while empirical studies often focus on one [12,13,63] or the other [16,64,65]. Our results allow us to think more concretely about how to choose interventions and thereby target schistosomiasis control. Reducing exposure through behavior change interventions that minimize contact with water is likely to contribute more to morbidity control (e.g., reductions in infection intensity) than transmission control (e.g., reductions in infection presence). Reducing vulnerability through the provision and use of piped water infrastructure will give people an alternative to surface water, allowing them to avoid risky

exposures and thus, lower infection intensity. Reducing hazard in the environment through snail control interventions will be essential to reducing transmission and the likelihood of acquiring and accumulating infections from the environment. Understanding the interplay between different components of risk in a specific setting can facilitate the design of effective and efficient context-specific control strategies.

In this study, we u[illegible]rimary data sets collected by three distinct teams under the same project in the same study villages: (1) collection, processing and diagnosis of *S. haematobium* infection from urine samples collected from school-aged children on a yearly basis with associated treatment with praziquantel, (2) exhaustive sampling of snail populations and their habitats in the water access points in all villages, and (3) socio-economic surveys in all the households where school-aged children in the parasitology study lived. All three data streams, combined with data on the spatial relationship between water access points and surveyed households, were important for explaining patterns of infection in children. Our results underscore the importance of interdisciplinary and mixed methods research when addressing complex socio-environmental problems like the transmission of schistosomiasis and other infectious diseases.

Measures of exposure, hazard, and vulnerability

We learned how different measures of exposure, hazard and vulnerability fit the infection data. The simplest exposure index, which used seven-day recall of visits to a surface freshwater access point, fit infection intensity data better than did more complex exposure metrics that included information on the reported frequency, duration and extent of contact experienced for specific activities. There are several potential explanations for this finding. This simple index was derived from a single survey question that was posed specifically for the children enrolled in

the parasitology study. In contrast, the activity-specific estimates were derived from a survey module that prompted individual-level information from all members of that household. Because of the volume of information that respondents were asked to provide (e.g., whether a person performed each of six water contact activities for all members of the household), the accuracy and precision of the responses may have suffered.

Other studies have found weak or no association with exposure measures that account for multiple aspects of exposure. One study in a similar setting in northern Senegal found all of their exposure indices to be correlated with each other but not with the presence or intensity of *S. mansoni* infection [54]. In the study from which we drew data on activity-specific duration of water contact, a relationship between water contact and intensity of *S. mansoni* infection was only detected after accounting for body surface area and the time of day of exposure [39]. In a study of *S. haematobium* infection in Zanzibar, a set of exposure indices that incorporated varying combinations of frequency, duration and body surface area were found to be positively associated with infection, but the strength of the association decreased with the complexity of the exposure index used [66]. Moreover, the distinct ecologies of schistosome species and their intermediate snail hosts and the methods used to measure water contact may confound the true relationship between water contact and infection.

Similarly, malacological hazard data often have weak or no association with infection. In one study in China, a single infected snail was found among 7,000 snails collected in a setting where prevalence of *S. japonicum* infection in people exceeded 25% [67]. Prevalence of human infection of 30-40% has been observed in lake regions of East Africa where snails have been found to occur in very low numbers [68,69]. An ecological study of snail ecology in Senegal – the one from which ecological data in this study is drawn – found area of snail habitat to be a

superior predictor of human infection compared to seemingly more proximate predictors including density of infected snails [70]. The current study builds on these findings by matching individual-level infection to the water access site nearest to the household (in addition to village-level metrics used in the previous study) and accounting for the spatial relationship between the household and the water access site.

Given the shortcomings of previous studies on water contact and snail ecology, we found that accounting for the spatial relationship between people and the environmental hazard improved the fit of *S. haematobium* presence models. Hazard indices scaled by the distance to the nearest water point fit the infection presence data better than the other hazard indices, though this was not the case for infection intensity data. Previous studies have found proximity to water access points to be an important explanatory variable of schistosome infection in endemic settings [66,71–74]. Another found that water contact was only related to re-infection after accounting for the spatial distance between people and the hazard in their environment [19]. Such proximity may be an important aspect of vulnerability that makes it difficult to avoid exposure to hazardous water.

Similarly, hazard indices that dealt with the site nearest to the child's household fit the infection presence data better than village-level aggregations of snail habitat. This finding contradicts studies that found schistosomiasis to be associated with human behavior and snail ecology primarily at the community level [75–77]. However, because water contact activity for most people tends to occur at just one or two sites [76,77] and since distance to water is commonly associated with infection [72,73], it is possible that the distance-adjusted hazard indices capture the hazard where exposure is most likely to occur for people living in a given household.

Measures of association between components of risk and infection

We found that no single model best approximated either the presence or intensity of *S. haematobium* infection. Rather, a set of models comprised the evidence for our conclusions. Without a single best-approximating model, we used model averaging to generate point estimates and confidence intervals of the association between our two infection outcomes and all explanatory variables appearing in both model sets.

Associations between demographic control variables and infection were consistent across the two outcomes. These variables – particularly child sex and village location – had the strongest relationship with infection of all variables included in all models. This finding speaks to the importance of demographics in the dynamics of schistosome infection [78,79] and the need for analyses that examine heterogeneity across sex and geographic strata [77,80].

We found that exposure was associated with an increase in both infection presence and intensity, although the confidence interval for the estimate of infection presence included the null value. Consistent with our relative importance findings, these estimates support the notion that the amount of exposure influences the accumulation of worm burden more than the acquisition of infection. The substantial support we found for vulnerability as an explanatory variable of infection presence may approximate the presence (rather than amount) of exposure. Given the lifespan of adult schistosomes in the human host (3–10 years) [78,81,82], a single infection resulting from a single exposure may be present in the body for years until a person is treated.

Even though model-averaging found indices of vulnerability to have wide confidence intervals, there was suggestive evidence of a dose-response between the odds of infection and the number of activities for which a household uses surface water. Children living in households that relied on surface water for multiple activities were more likely to be infected than those whose

households relied on surface water for a single activity. While this may be the result of a lack of alternative sources of water (such as wells or piped water), it is also important to note that water contact activities often have social value [83], making the use of surface water for social activities like laundry a matter of preference. It is also possible that available alternative sources of water may not be suitable for particular activities, making surface water preferable in some cases [65,84].

The asset-based SES index was the poorest index of vulnerability (ΔAIC = 9.48 and 6.40 among vulnerability-only presence intensity models, respectively), outperformed by indices that represented access to and use of water and sanitation infrastructure. It is possible that SES is a relatively poor predictor of infection because there is not much variability in ownership of durable assets across our study population. Similarly, while the household dependence on surface water is not evenly distributed across SES quintiles, we found similar proportions of households in highest and lowest SES quintiles that use surface water for at least some household needs. It is easy to assume that dependence on surface water will be highly correlated with SES, but that may not be the case when decisions to continue using surface water are made for other reasons. Similarly, because our study focuses on children who go to school and play with each other, the influence of SES on infection outcomes may not be as pronounced as they would be among adults, for whom occupation and household factors are stronger determinants of exposure. Consequently, SES, as we measured it, may not be a strong determinant of water contact or schistosome infection outcomes.

Finally, our measures of hazard were improved by accounting for snail habitat in the water access site nearest to the household, and in the case of models of *S. haematobium* presence, the best metric accounted for distance between households and the nearest water point.

These methodological insights highlight the important role of spatial relationships in schistosomiasis risk and the value of incorporating spatial data and analysis into future research.

Limitations

The limitations of this study are grounded in the challenge of measuring behavioral and ecological phenomena that are dynamic in both space and time. In using multiple methods to measure water contact behavior, specifically, we struck a balance between collecting complete information across the many facets of water contact behavior and collecting data from enough participants to fit statistical models. To do this, we supplemented individual-level information on regular water contact activities for all participants with household-level survey data on frequency of contact, literature-based estimates on duration of contact and interview data on body surface area exposure.

While incorporating frequency, duration and body surface area into exposure indices was meant to triangulate around the different dimensions of water contact, we note that the complex metrics were often outperformed by a simple metric of exposure derived from a single survey question. The poor fit of the complex exposure metrics may reflect our use of aggregated data on frequency, duration and extent of exposure to the household and village levels, as they may obscure important individual-level heterogeneities [53,85]. Ultimately, logistics limited our ability to capture water contact behavior comprehensively and precisely for hundreds of children across more than a dozen villages, each with its own complex landscape of water contact. Because we did not record individual episodes of water contact for each participant, we were not able to determine the time of day when water contact occurred, a key criterion for exposure based on the chronobiology of cercarial release from snails [86,87].

The issues implicit in the choice of methods for measuring water contact (e.g., recall and/or social desirability bias for questionnaire-based studies and failure to capture contact at other sites for studies based on direct observations at a single site) may be resolved in future studies by combining questionnaire-based or activity diary methods with the use of wearable GPS data loggers to collect more accurate and precise data on the spatial and temporal dynamics of water contact behavior. Such methods have been used in China [88], Uganda [83] and Cameroon [89] and have been found to be widely acceptable [90].

Finally, this study examines the convergence of the processes of exposure, hazard, and vulnerability for just one of the two schistosome species that circulate and cause disease in this setting. *S. mansoni* is co-endemic, circulating within different species of snail intermediate hosts with distinct habitat associations. The human behaviors associated with contamination of the environment also differ for *S. mansoni* compared to *S. haematobium*. For these reasons, the particularities of *S. mansoni* transmission may not be governed by the same relationships between exposure, hazard, and vulnerability.

Conclusion

In summary, we find that hazard, as measured by the area of snail habitat in the water access point nearest a household, and vulnerability, in terms of surface water use and access to sanitation at the household level, most contribute to the acquisition of *S. haematobium* infections. In contrast, exposure, approximated by a self-reported categorical frequency of contact with surface water per week, and hazard most contributes to the accumulation of parasites in school-age children, and thus to infection intensity. Together, our findings underscore the importance of all three components of risk, which act together across time and space to facilitate the acquisition and accumulation of schistosome infections in an endemic setting with high incidence of post-

treatment re-infection. They highlight how interventions to complement MDA may be strategically deployed to reduce the risk of post-treatment re-infection.

While our data are specific to the context in which we collected them, the insights about the interaction among different components of risk and their impact on different metrics of infection may apply in other endemic settings and generate new knowledge about the processes that lead to the occurrence of persistent hot spots. Such an approach is critical to both controlling the severe morbidity of schistosomiasis and reducing the transmission of parasites in the environment to achieving elimination in endemic regions. Moreover, building a more holistic understanding of infectious disease risk will improve our ability to intervene at the point where a source of infection and a susceptible person meet.

Acknowledgements

The authors thank Dr. Seynabou Ndiaye and Dr. Tidiane Ly for their work caring for the health of the children enrolled in the parasitology study. The authors also thank the staff of CRB-EPLS who played critical roles in collecting and processing the parasitology data, the ecological data and the household survey data as well as providing logistical and administrative support of the field work. The authors also thank Ashley Hazel for her helpful comments on an early draft of this manuscript. Finally, we thank four reviewers for their constructive comments on the manuscript.

References

1. Cardona O-D, van Aalst MK, Birkmann J, Fordham M, McGregor G, Perez R, et al. Determinants of Risk: Exposure and Vulnerability. In: Field CB, Barros V, Stocker TF, Dahe Q, editors. Managing the Risks of Extreme Events and Disasters to Advance Climate Change Adaptation. Cambridge: Cambridge University Press; 2012. pp. 65–108. doi:10.1017/CBO9781139177245.005

2. Garchitorena A, Sokolow SH, Roche B, Ngonghala CN, Jocque M, Lund A, et al. Disease Ecology, Health and the Environment: a framework to account for ecological and socio-economic drivers in the control of neglected tropical diseases. Philos Trans R Soc Lond B Biol Sci. 2017;372: 20160128. doi:10.1098/rstb.2016.0128

3. Johnson PTJ, de Roode JC, Fenton A. Why infectious disease research needs community ecology. Science. 2015;349: 1259504–1259504. doi:10.1126/science.1259504

4. Eisenberg JNS, Desai MA, Levy K, Bates SJ, Liang S, Naumoff K, et al. Environmental determinants of infectious disease: A framework for tracking causal links and guiding public health research. Environmental Health Perspectives. 2007;115: 1216–1223. doi:10.1289/ehp.9806

5. McClure M, Diuk-Wasser M. Reconciling the Entomological Hazard and Disease Risk in the Lyme Disease System. International Journal of Environmental Research and Public Health. 2018;15: 1048. doi:10.3390/ijerph15051048

6. Porta M. A dictionary of epidemiology. 6th ed. Oxford, UK: Oxford University Press; 2015.

7. Jones KE, Patel NG, Levy MA, Storeygard A, Balk D, Gittleman JL, et al. Global trends in emerging infectious diseases. Nature. 2008;451: 990–993. doi:10.1038/nature06536

8. Hosseini PR, Mills JN, Prieur-Richard A-H, Ezenwa VO, Bailly X, Rizzoli A, et al. Does the impact of biodiversity differ between emerging and endemic pathogens? The need to separate the concepts of hazard and risk. Phil Trans R Soc B. 2017;372: 20160129. doi:10.1098/rstb.2016.0129

9. US EPA. Guidelines for Human Exposure Assessment Risk Assessment Forum. Washington, DC: US EPA; 2019 Oct p. 223. Report No.: EPA/100/B-19/001.

10. Hagenlocher M, Castro MC. Mapping malaria risk and vulnerability in the United Republic of Tanzania: a spatial explicit model. Popul Health Metrics. 2015;13: 1–14. doi:10.1186/s12963-015-0036-2

11. Kienberger S, Hagenlocher M. Spatial-explicit modeling of social vulnerability to malaria in East Africa. Int J Health Geogr. 2014;13: 29. doi:10.1186/1476-072X-13-29

12. Rabone M, Wiethase JH, Allan F, Gouvras AN, Pennance T, Hamidou AA, et al. Freshwater snails of biomedical importance in the Niger River Valley: evidence of temporal and spatial patterns in abundance, distribution and infection with Schistosoma spp. Parasit Vectors. 2019;12: 1–20. doi:10.1186/s13071-019-3745-8

13. Mutuku MW, Laidemitt MR, Beechler BR, Mwangi IN, Otiato FO, Agola EL, et al. A Search for Snail-Related Answers to Explain Differences in Response of Schistosoma mansoni to Praziquantel

Treatment among Responding and Persistent Hotspot Villages along the Kenyan Shore of Lake Victoria. The American Journal of Tropical Medicine and Hygiene. 2019;101: 65–77. doi:10.4269/ajtmh.19-0089

14. Madsen H, Stauffer J. The burrowing behaviour of *Bulinus nyassanus* , intermediate host of *Schistosoma haematobium* , in Lake Malaŵi. African Journal of Aquatic Science. 2012;37: 113–116. doi:10.2989/16085914.2012.666383

15. Kloos H, Rodrigues JCAP, Pereira WR, Vel??squez-Mel??ndez G, LoVerde P, Oliveira RC, et al. Combined methods for the study of water contact behavior in a rural schistosomiasis-endemic area in Brazil. Acta Tropica. 2006;97: 31–41. doi:10.1016/j.actatropica.2005.08.006

16. De Moira AP, Fulford AJC, Kabatereine NB, Kazibwe F, Ouma JH, Dunne DW, et al. Microgeographical and tribal variations in water contact and Schistosoma mansoni exposure within a Ugandan fishing community. Tropical Medicine and International Health. 2007;12: 724–735. doi:10.1111/j.1365-3156.2007.01842.x

17. Gazzinelli A, Oliveira-Prado R, Matoso LF, Veloso BM, Andrade G, Kloos H, et al. Schistosoma mansoni reinfection: Analysis of risk factors by classification and regression tree (CART) modeling. PLoS ONE. 2017;12: e0182197.

18. Scott JT, Diakhate M, Vereecken K, Fall A, Diop M, Ly A, et al. Human water contacts patterns in Schistosoma mansoni epidemic foci in northern Senegal change according to age, sex and place of residence, but are not related to intensity of infection. Tropical Medicine and International Health. 2003;8: 100–108. doi:10.1046/j.1365-3156.2003.00993.x

19. Seto E, Lee YJ, Liang S, Zhong B. Individual and village-level study of water contact patterns and Schistosoma japonicum infection in mountainous rural China. Tropical Medicine & International Health. 2007;12: 1199–1209. doi:10.1111/j.1365-3156.2007.01903.x

20. Grimes JET, Croll D, Harrison WE, Utzinger J, Freeman MC, Templeton MR, et al. The roles of water, sanitation and hygiene in reducing schistosomiasis: A review. Parasit Vectors. 2015;8: 156. doi:10.1186/s13071-015-0766-9

21. Secor WE. Water-based interventions for schistosomiasis control. Pathog Glob Health. 2014;108: 246–54. doi:10.1179/2047773214Y.0000000149

22. Cronin T, Sheppard J, Wildt G de. Health-seeking behaviour for schistosomiasis: a systematic review of qualitative and quantitative literature. Pan Afr Med J. 2013;16. doi:10.11604/pamj.2013.16.130.3078

23. World Health Organization. PCT databank. In: WHO [Internet]. World Health Organization; 2020 [cited 22 Apr 2020]. Available: http://www.who.int/neglected_diseases/preventive_chemotherapy/sch/en/

24. World Health Organization. Schistosomiasis. World Health Organization; 2016 [cited 25 Jan 2016]. Available: http://www.who.int/schistosomiasis/en/

25. Fenwick A, Rollinson D, Southgate V. Implementation of Human Schistosomiasis Control: Challenges and Prospects. Adv Parasitol. 2006;61. doi:10.1016/S0065-308X(05)61013-5

26. King CH, Bertsch D. Historical perspective: snail control to prevent schistosomiasis. PLoS Negl Trop Dis. 2015;9: e0003657. doi:10.1371/journal.pntd.0003657

27. Webster JP, Molyneux DH, Hotez PJ, Fenwick A. The contribution of mass drug administration to global health: past, present and future. Philosophical Transactions of the Royal Society of London B: Biological Sciences. 2014;369: 20130434. doi:10.1098/rstb.2013.0434

28. Kittur N, King CH, Jr CHC, Kinung'hi S, Mwinzi PNM, Karanja DMS, et al. Persistent Hotspots in Schistosomiasis Consortium for Operational Research and Evaluation Studies for Gaining and Sustaining Control of Schistosomiasis after Four Years of Mass Drug Administration of Praziquantel. The American Journal of Tropical Medicine and Hygiene. 2019;101: 617–627. doi:10.4269/ajtmh.19-0193

29. Ross AGP, Olveda RM, Chy D, Olveda DU, Li Y, Harn DA, et al. Can mass drug administration lead to the sustainable control of schistosomiasis? Journal of Infectious Diseases. 2015;211: 283–289. doi:10.1093/infdis/jiu416

30. Wiegand RE, Mwinzi PNM, Montgomery SP, Chan YL, Andiego K, Omedo M, et al. A Persistent Hotspot of Schistosoma mansoni Infection in a Five-Year Randomized Trial of Praziquantel Preventative Chemotherapy Strategies. J Infect Dis. 2017;216: 1425–1433. doi:10.1093/infdis/jix496

31. Sow S, de Vlas SJ, Engels D, Gryseels B. Water-related disease patterns before and after the construction of the Diama dam in northern Senegal. Ann Trop Med Parasitol. 2002;96: 575–586. doi:10.1179/000349802125001636

32. Sokolow SH, Huttinger E, Jouanard N, Hsieh MH, Lafferty KD, Kuris AM, et al. Reduced transmission of human schistosomiasis after restoration of a native river prawn that preys on the snail intermediate host. Proc Natl Acad Sci U S A. 2015;112: 9650–9655. doi:10.1073/pnas.1502651112

33. Wood CL, Sokolow SH, Jones IJ, Chamberlin AJ, Lafferty KD, Kuris AM, et al. Precision mapping of snail habitats provides a powerful indicator of human schistosomiasis transmission. Proc Natl Acad Sci USA. 2019;116: 23182–23191. doi:https://doi.org/10.1073/pnas.1903698116

34. Stelma FF, Talla I, Polman K, Niang M, Sturrock RF, Deelder AM, et al. Epidemiology of Schistosoma mansoni infection in a recently exposed community in northern Senegal. Am J Trop Med Hyg. 1993;49: 701–6.

35. Meurs L, Mbow M, Vereecken K, Menten J, Mboup S, Polman K. Epidemiology of mixed Schistosoma mansoni and Schistosoma haematobium infections in northern Senegal. Int J Parasitol. 2012;42: 305–11. doi:10.1016/j.ijpara.2012.02.002

36. Connor D, Comas J, Macpherson H-G, Mateos L. Impact of small-holder irrigation on the agricultural production, food supply and economic prosperity of a representative village beside the Senegal River, Mauritania. Agric Syst. 2008;96: 1–15. doi:10.1016/j.agsy.2007.04.001

37. Trienekens SCM, Faust CL, Meginnis K, Pickering L, Ericsson O, Nankasi A, et al. Impacts of host gender on Schistosoma mansoni risk in rural Uganda—A mixed-methods approach. PLOS Neglected Tropical Diseases. 2020;14: e0008266. doi:10.1371/journal.pntd.0008266

38. Chandiwana SK, Woolhouse ME. Heterogeneities in water contact patterns and the epidemiology of Schistosoma haematobium. Parasitology. 1991;103 Pt 3: 363–370. doi:10.1017/S0031182000059874

39. Sow S, de Vlas SJ, Stelma F, Vereecken K, Gryseels B, Polman K. The contribution of water contact behavior to the high Schistosoma mansoni Infection rates observed in the Senegal River Basin. BMC Infect Dis. 2011;11: 198. doi:10.1186/1471-2334-11-198

40. Sturrock RF. Schistosomiasis epidemiology and control: how did we get here and where should we go? Mem Inst Oswaldo Cruz. 2001;96: 17–27. doi:10.1590/S0074-02762001000900003

41. Gryseels B, Polman K, Clerinx J, Kestens L. Human schistosomiasis. Lancet. 2006;368: 1106–18. doi:10.1016/S0140-6736(06)69440-3

42. Liu R, Dong H-F, Guo Y, Zhao Q-P, Jiang M-S. Efficacy of praziquantel and artemisinin derivatives for the treatment and prevention of human schistosomiasis: a systematic review and meta-analysis. Parasit Vectors. 2011;4: 201. doi:10.1186/1756-3305-4-201

43. Zwang J, Olliaro PL. Clinical Efficacy and Tolerability of Praziquantel for Intestinal and Urinary Schistosomiasis — A Meta-analysis of Comparative and Non-comparative Clinical Trials. PLoS Neglected Tropical Diseases. 2014;8: e3286. doi:10.1371/journal.pntd.0003286

44. Lund C, Browder N. The estimation of areas of burns. Surgery Gynecology & Obstetrics. 1944;79: 352–358.

45. Mosteller RD. Simplified Calculation of Body-Surface Area. N Engl J Med. 1987;317: 1098–1098. doi:10.1056/NEJM198710223171717

46. Filmer D, Pritchett LH. Estimating Wealth Effects Without Expenditure Data—Or Tears: An Application To Educational Enrollments In States Of India. Demography. 2001;38: 115–132. doi:10.1353/dem.2001.0003

47. Vyas S, Kumaranayake L. Constructing socio-economic status indices: how to use principal components analysis. Health Policy Plan. 2006;21: 459–468. doi:10.1093/heapol/czl029

48. R Core Team. R: A language and environmental for statistical computing. Vienna, Austria: R Foundation for Statistical Computing; 2018. Available: https://www.R-project.org/

49. Bates D, Mächler M, Bolker B, Walker S. Fitting Linear Mixed-Effects Models Using **lme4**. J Stat Soft. 2015;67. doi:10.18637/jss.v067.i01

50. Brooks ME, Kristensen K, van Benthem KJ, Magnusson A, Berg CW, Nielsen A, et al. glmmTMB Balances Speed and Flexibility Among Packages for Zero-inflated Generalized Linear Mixed Modeling. R J. 2017;9: 378–400.

51. Burnham KP, Anderson DR. Model Selection and Multimodel Inference: A Practical Information-Theoretic Approach. 2nd ed. New York: Springer; 2002.

52. Mazerolle, Marc J. AICcmodavg: Model selection and multimodel inference based on (Q)AIC(c). 2019. Available: https://cran.r-project.org/web/packages/AICcmodavg/AICcmodavg.pdf

53. Bundy DAP, Blumenthal UJ. Human behavior and the epidemiology of helminth infections: the role of behavior in exposure to infection. In: Barnard CJ, Behnke JM, editors. Parasitism and Host Behavior1. London: Taylor & Francis Ltd; 990. pp. 264–289.

54. Scott JT, Diakhate M, Vereecken K, Fall A, Diop M, Ly A, et al. Human water contacts patterns in Schistosoma mansoni epidemic foci in northern Senegal change according to age, sex and place of residence, but are not related to intensity of infection. Tropical Medicine and International Health. 2003;8: 100–108. doi:10.1046/j.1365-3156.2003.00993.x

55. Booth M, Vennervald BJ, Kabatereine NB, Kazibwe F, Ouma JH, Kariuki CH, et al. Hepatosplenic morbidity in two neighbouring communities in Uganda with high levels of Schistosoma mansoni infection but very different durations of residence. Transactions of the Royal Society of Tropical Medicine and Hygiene. 2004;98: 125–136. doi:10.1016/S0035-9203(03)00018-X

56. Dalton PR. A socioecological approach to the control of Schistosoma mansoni in St Lucia. Bull World Health Organ. 1976;54: 587–95.

57. Utzinger J, N'goran EK, Caffrey CR, Keiser J. From innovation to application: social-ecological context, diagnostics, drugs and integrated control of schistosomiasis. Acta tropica. 2011;120 Suppl: S121-37. doi:10.1016/j.actatropica.2010.08.020

58. Krauth SJ, Balen J, Gobert GN, Lamberton PHL. A Call for Systems Epidemiology to Tackle the Complexity of Schistosomiasis, Its Control, and Its Elimination. Tropical Medicine and Infectious Disease. 2019;4: 21. doi:10.3390/tropicalmed4010021

59. Gazzinelli A, Correa-Oliveira R, Yang G-J, Boatin BA, Kloos H. A Research Agenda for Helminth Diseases of Humans: Social Ecology, Environmental Determinants, and Health Systems. Garba A, editor. PLoS Negl Trop Dis. 2012;6: e1603. doi:10.1371/journal.pntd.0001603

60. Gurarie D, Seto EYW. Connectivity sustains disease transmission in environments with low potential for endemicity: modelling schistosomiasis with hydrologic and social connectivities. J R Soc Interface. 2009;6: 495–508. doi:10.1098/rsif.2008.0265

61. Perez-Saez J, Mari L, Bertuzzo E, Casagrandi R, Sokolow SH, De Leo GA, et al. A Theoretical Analysis of the Geography of Schistosomiasis in Burkina Faso Highlights the Roles of Human Mobility and Water Resources Development in Disease Transmission. PLoS neglected tropical diseases. 2015;9: e0004127. doi:10.1371/journal.pntd.0004127

62. Perez-Saez J, Mande T, Ceperley N, Bertuzzo E, Mari L, Gatto M, et al. Hydrology and density feedback control the ecology of intermediate hosts of schistosomiasis across habitats in seasonal climates. Proceedings of the National Academy of Sciences of Sciences. 2016;113: 6427–6432.

63. Sturrock RF, Talla I, Niang M, Capron A. Seasonality in the transmission of schistosomiasis and in populations of its snail intermediate hosts in and around a sugar irrigation scheme at Richard Toll, Senegal. Parasitology. 2001;123: S77–S89. doi:10.1017/S0031182001008125

64. Lima e Costa MFF, Magalhães MHA, Rocha RS, Antunes CM, Katz N. Water-contact patterns and socioeconomic variables in the epidemiology of schistosomiasis mansoni in an endemic area in Brazil. Bulletin of the World Health Organization. 1987;65: 57–66.

65. Kulinkina AV, Kosinski KC, Adjei MN, Osabutey D, Gyamfi BO, Biritwum N-K, et al. Contextualizing Schistosoma haematobium transmission in Ghana: assessment of diagnostic techniques and individual and community water-related risk factors. Acta Tropica. 2019 [cited 18 Mar 2019]. doi:10.1016/j.actatropica.2019.03.016

66. Rudge JW, Stothard JR, Basáñez M-G, Mgeni AF, Khamis IS, Khamis AN, et al. Micro-epidemiology of urinary schistosomiasis in Zanzibar: Local risk factors associated with distribution of infections among schoolchildren and relevance for control. Acta Trop. 2008;105: 45–54. doi:10.1016/j.actatropica.2007.09.006

67. Carlton EJ, Bates MN, Zhong B, Seto EYW, Spear RC. Evaluation of Mammalian and Intermediate Host Surveillance Methods for Detecting Schistosomiasis Reemergence in Southwest China. PLoS Negl Trop Dis. 2011;5: e987. doi:10.1371/journal.pntd.0000987

68. Madsen H, Bloch P, Makaula P, Phiri H, Furu P, Stauffer JR. Schistosomiasis in Lake Malaŵi Villages. EcoHealth. 2011;8: 163–176. doi:10.1007/s10393-011-0687-9

69. Gryseels B. The epidemiology of schistosomiasis in Burundi and its consequences for control. Transactions of the Royal Society of Tropical Medicine and Hygiene. 1991;85: 626–633. doi:10.1016/0035-9203(91)90371-5

70. Wood CL, Sokolow SH, Jones IJ, Chamberlin AJ, Lafferty KD, Kuris AM, et al. Precision mapping of snail habitats provides a powerful indicator of human schistosomiasis transmission. Proc Natl Acad Sci USA. 2019;116: 23182–23191. doi:https://doi.org/10.1073/pnas.1903698116

71. Kloos H, Fulford AJC, Butterworth AE, Sturrock RF, Ouma JH, Kariuki HC, et al. Spatial patterns of human water contact and Schistosoma mansoni transmission and infection in four rural areas in Machakos District, Kenya. Social Science & Medicine. 1997;44: 949–968. doi:10.1016/S0277-9536(96)00218-3

72. Pennance T, Person B, Muhsin MA, Khamis AN, Muhsin J, Khamis IS, et al. Urogenital schistosomiasis transmission on Unguja Island, Zanzibar: characterisation of persistent hot-spots. Parasit Vectors. 2016;9. doi:10.1186/s13071-016-1847-0

73. Clennon JA, King CH, Muchiri EM, Kariuki HC, Ouma JH, Mungai P, et al. Spatial patterns of urinary schistosomiasis infection in a highly endemic area of coastal Kenya. Am J Trop Med Hyg. 2004;70: 443–448.

74. Walker JW, Kittur N, Binder S, Castleman JD, Drake JM, Campbell CH, et al. Environmental Predictors of Schistosomiasis Persistent Hotspots following Mass Treatment with Praziquantel. The

American Journal of Tropical Medicine and Hygiene. 2020;102: 328–338. doi:10.4269/ajtmh.19-0658

75. Raso G, Matthys B, N 'goran EK, Tanner M, Vounatsou P, Utzinger J. Spatial risk prediction and mapping of Schistosoma mansoni infections among schoolchildren living in western Côte d'Ivoire. Parasitology. 2005;131: 97–108. doi:10.1017/S0031182005007432

76. Woolhouse MEJ, Watts CH, Chandiwana SK. Heterogeneities in transmission rates and the epidemiology of schistosome infection. Proceedings of the Royal Society of London Series B: Biological Sciences. 1991;245: 109–114. doi:10.1098/rspa.1983.0054

77. Woolhouse ME, Etard JF, Dietz K, Ndhlovu PD, Chandiwana SK. Heterogeneities in schistosome transmission dynamics and control. Parasitology. 1998;117: 475–482. doi:10.1017/S003118209800331X

78. Bustinduy AL, King CH. Schistosomiasis. Manson's Tropical Infectious Diseases. Elsevier; 2014. pp. 698-725.e6. doi:10.1016/B978-0-7020-5101-2.00053-4

79. Huang Y, Manderson L. Schistosomiasis and the social patterning of infection. Acta Trop. 1992;51: 175–194. doi:10.1016/0001-706X(92)90037-X

80. Mari L, Ciddio M, Casagrandi R, Perez-Saez J, Bertuzzo E, Rinaldo A, et al. Heterogeneity in schistosomiasis transmission dynamics. Journal of Theoretical Biology. 2017;432: 87–99. doi:10.1016/j.jtbi.2017.08.015

81. Goddard MJ, Jordan P. On the longevity of Schistosoma mansoni in man on St. Lucia, West Indies. Transactions of the Royal Society of Tropical Medicine and Hygiene. 1980;74: 185–191. doi:10.1016/0035-9203(80)90240-0

82. Fulford AJC, Butterworth AE, Ouma JH, Sturrock RF. A statistical approach to schistosome population dynamics and estimation of the life-span of Schistosoma mansoni in man. Parasitology. 1995;110: 307–316. doi:10.1017/S0031182000080896

83. Seto E, Sousa-Figueiredo JC, Betson M, Byalero C, Kabatereine NB, Stothard JR. Patterns of intestinal schistosomiasis among mothers and young children from Lake Albert, Uganda: water contact and social networks inferred from wearable global positioning system dataloggers. Geospatial health. 2012;7: 1–13. doi:10.4081/gh.2012.99

84. Krauth SJ, Musard C, Traoré SI, Zinsstag J, Achi LY, N'Goran EK, et al. Access to, and use of, water by populations living in a schistosomiasis and fascioliasis co-endemic area of northern Côte d'Ivoire. Acta Trop. 2015;149: 179–85. doi:10.1016/j.actatropica.2015.05.019

85. Susser M. Does risk factor epidemiology put epidemiology at risk? Peering into the future. Journal of epidemiology and community health. 1998;52: 608–11. doi:10.1136/JECH.52.10.608

86. Mouchet F, Theron A, Bremond P, Sellin E, Sellin B. Pattern of cercarial emergence of Schistosoma curassoni from Niger and comparison with three sympatric species of schistosomes. J Parasitol. 1992;78: 61–63. doi:10.2307/3283687

87. Combes C, Fournier A, Mone H, Theron A. Behaviours in trematode cercariae that enhance parasite transmission: patterns and processes. Parasitology. 1994;109: S3–S13.

88. Seto E, Knapp F, Zhong B, Yang C. The use of a vest equipped with a global positioning system to assess water-contact patterns associated with schistosomiasis. Geospat Health. 2007;1: 233–41. doi:10.4081/gh.2007.271

89. Macklin G, Stanton MC, Tchuem-Tchuenté LA, Stothard JR. A pilot study using wearable global positioning system data loggers to compare water contact levels: Schistosoma haematobium infection in pre-school-age children (PSAC) and their mothers at Barombi Kotto, Cameroon. Transactions of The Royal Society of Tropical Medicine and Hygiene. 2018 [cited 15 Jul 2018]. doi:10.1093/trstmh/try059

90. Paz-Soldan VA, Stoddard ST, Vazquez-Prokopec G, Morrison AC, Elder JP, Kitron U, et al. Assessing and maximizing the acceptability of global positioning system device use for studying the role of human movement in dengue virus transmission in Iquitos, Peru. Am J Trop Med Hyg. 2010;82: 723–30. doi:10.4269/ajtmh.2010.09-0496

Supporting Information

S1 Figure. Data flow diagram. Sample sizes resulting from the merging and cleaning of human data from parasitological and household surveys and removing data from manipulated sites.

S1 Table. Household survey items. Household survey items used in in this analysis, including the module to which an item belonged, the level at which it was measured as well as the wording of and response categories provided for each question

S2 Table. Duration and extent of water contact. Activity-specific values used to estimate duration and extent of water contact. Duration estimates were drawn from published research that took place in a similar setting and extent was estimated from body surface area interviews.

S3 Table. Household surface water use by socio-economic status. Frequencies (n (row %)) of household surface water use by quintiles of asset-based index of socio-economic status (SES).

S4 Table. Presence models. Full set of candidate models used for multi-model inference of the relative contributions of exposure, hazard, and vulnerability to *S. haematobium* infection presence.

S5 Table. Intensity models. Full set of candidate models used for multi-model inference of the relative contributions of exposure, hazard, and vulnerability to *S. haematobium* infection intensity.

S1 Checklist. STROBE checklist for cross-sectional studies

S2 Figure. Correlation plot. Correlations between all variables used in all models

Figure 1

Click here to access/download;Figure;Fig1_150.tiff

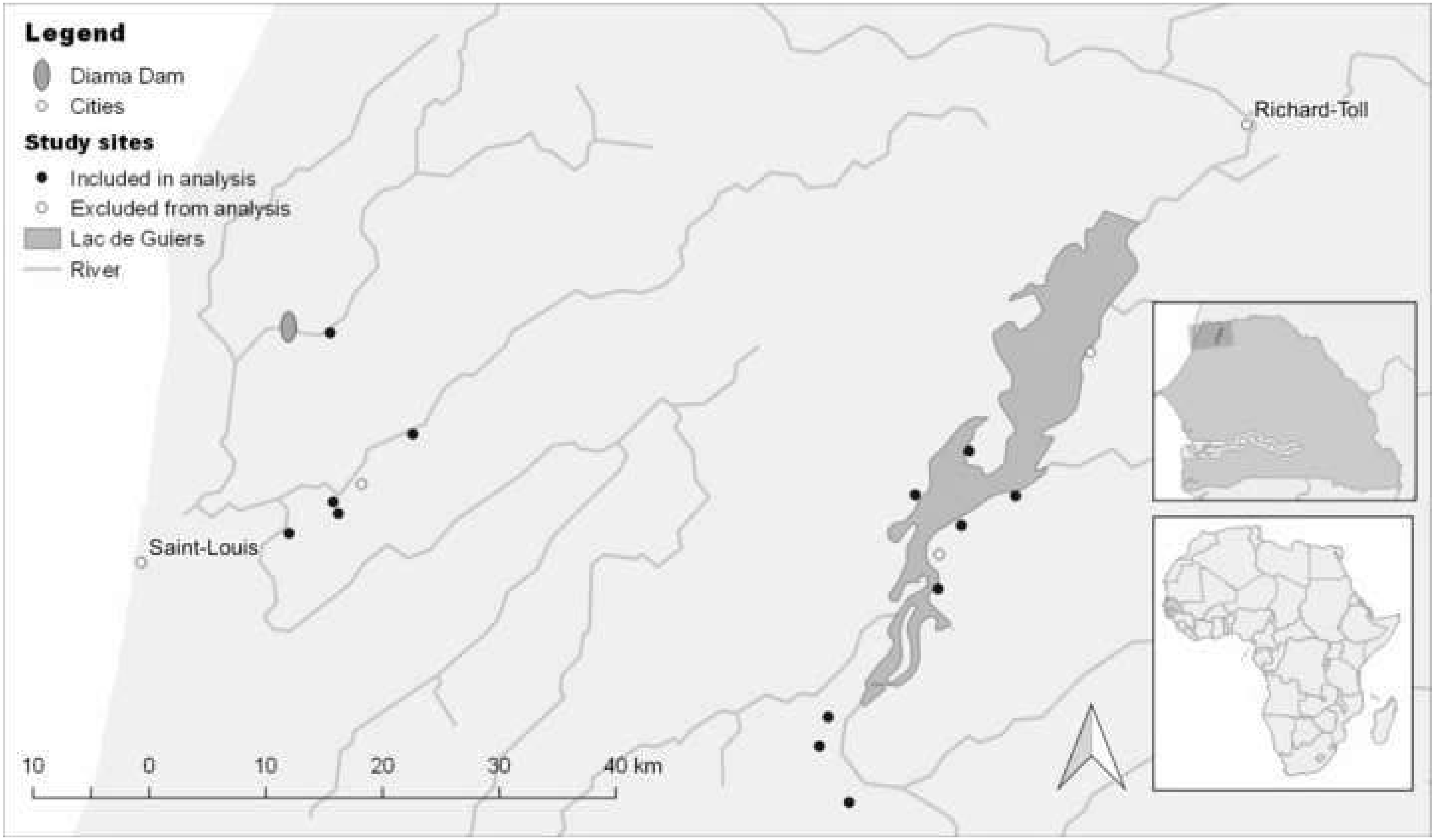

Figure 2

Click here to access/download;Figure;Figure 2.tiff

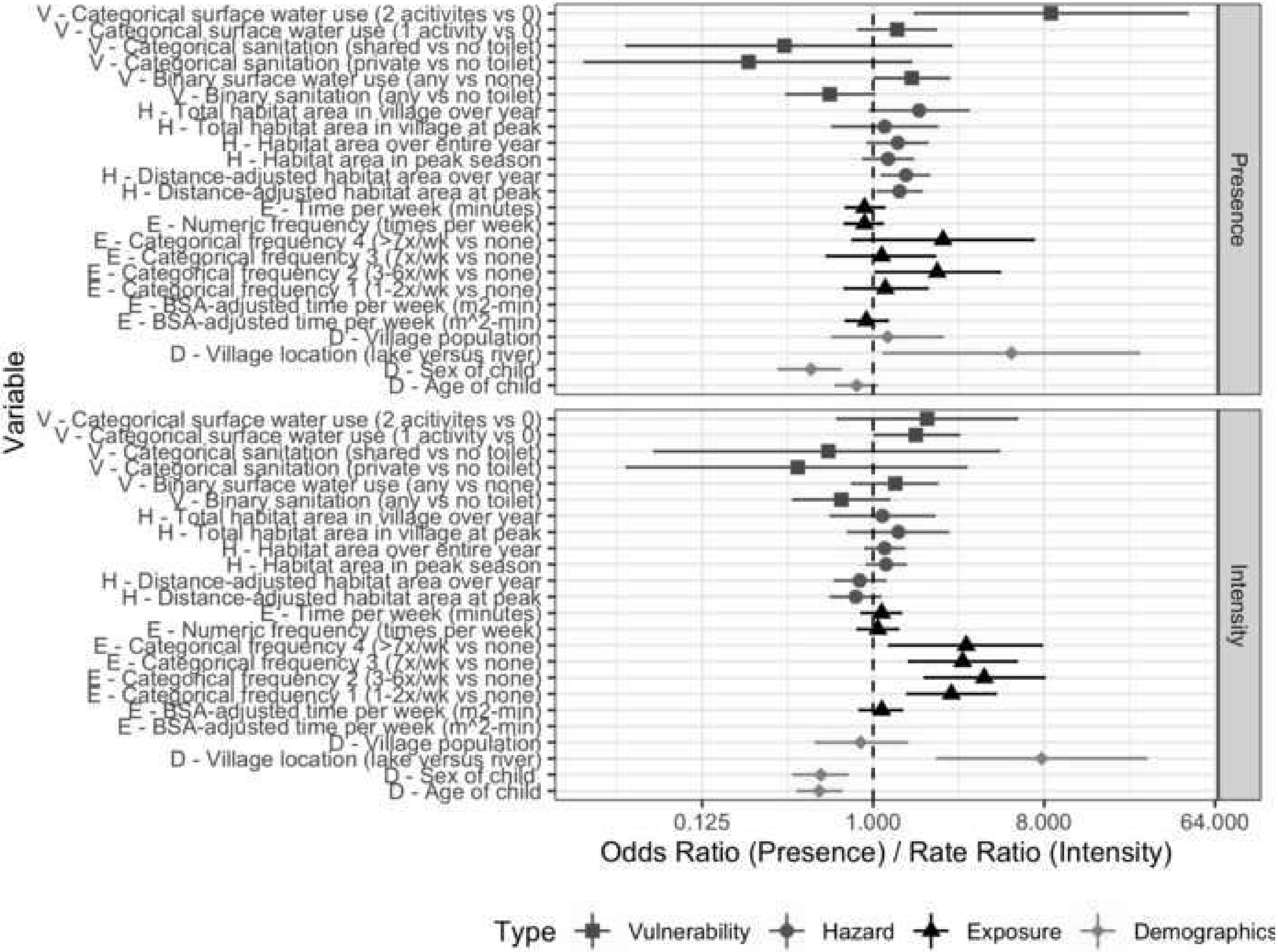

Click here to access/download
Supporting Information
S1 Figure.tif

Click here to access/download
Supporting Information
S1 Table.pdf

Click here to access/download
Supporting Information
S2 Table.pdf

Click here to access/download
Supporting Information
S3 Table.pdf

Click here to access/download
Supporting Information
S4 Table.xlsx

Click here to access/download
Supporting Information
S5 Table.xlsx

S1 Checklist

Click here to access/download
Supporting Information
Checklist S1.doc

S2 Figure

Click here to access/download
Supporting Information
S2 Figure.tiff

Dr. Poppy H. Lamberton
Deputy Editor
Dr. Anna Last
Associate Editor
PLOS Neglected Tropical Diseases

Dear Dr. Lamberton and Dr. Last:

Thank you for considering our manuscript (PNTD-D-20_02100) "Exposure, hazard, and vulnerability all contribute to *Schistosoma haematobium* re-infection in northern Senegal" for publication in PLOS Neglected Tropical Diseases. We thank you and all four reviewers for your help in greatly improving the manuscript. We have revised the manuscript according to the reviewers' comments and believe that the revised manuscript clarifies both the strengths and limitations of the paper as well as its novelty in bringing together primary data collection across the social and environmental dimensions of schistosome infection risk.

Specifically, we have consolidated the limitations of our study under a new "Limitations" subheading in the Discussion. We have also further supported statements in both the Introduction and Discussion sections with additional references and clarified several methodological details. You will find a point-by-point response of how we addressed each of the reviewers' comments below.

We reiterate that this manuscript is original and is not being considered for publication elsewhere. We have no conflicts of interest to disclose.

Thank you for consideration.

Sincerely,

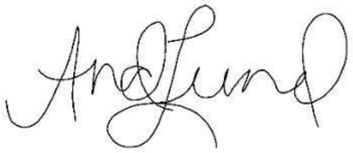

Andrea Lund, PhD MPH

Response to reviews

Thanks for this interesting paper which we would be happy to see resubmitted after undertaking the recommended changes. To me this reads as a mixed methods paper and could be sold more so on this aspect and the benefits that this provides, also helping to address the issues with smaller sample sizes, that would not be satisfactory for a purely quantitative study.

Response: Thank you for these comments. We have revised the manuscript according to these and additional suggestions below.

Reviewer's Responses to Questions

Key Review Criteria Required for Acceptance
As you describe the new analyses required for acceptance, please consider the following:

Methods
-Are the objectives of the study clearly articulated with a clear testable hypothesis stated?
-Is the study design appropriate to address the stated objectives?
-Is the population clearly described and appropriate for the hypothesis being tested?
-Is the sample size sufficient to ensure adequate power to address the hypothesis being tested?
-Were correct statistical analysis used to support conclusions?
-Are there concerns about ethical or regulatory requirements being met?

Reviewer #1: yes

Reviewer #2: (No Response)

Reviewer #3: Overall, the methods are appropriately chosen in response to the study questions/objectives. The only minor issues is related to interview described in the study. Only two interviews/village with a total of 30 interview were conducted – what are potential implications with the analysis with such a few samples? Also unclear how 210 activity-specific observations were conducted?

Reviewer #4: Methods are explained in detail. Some limitations with the use of existing data collected from the communities is discussed.

Response: Thank you for noting these important methodological details and limitations that were missing from the text. We have added a brief detail to clarify our description of the interview data collection in the Methods section (see line 258-259 in the tracked changes version), where we have clarified that the 210 activity-specific observations arise from interviewing 30 respondents about 7 water contact activities. We discuss the small sample size for interviews as a limitation of these data in the Discussion (see lines 876-928 in the tracked changes version).

Results
-Does the analysis presented match the analysis plan?
-Are the results clearly and completely presented?
-Are the figures (Tables, Images) of sufficient quality for clarity?

Reviewer #1: The authors ignore many relevant papers that have been published, which are given in a sticky note on the returned ms. They completely ignore the fact that very few snails have to be infected to result in a high (>87%) of school age children. They record the number of times that the children were in the water but they do not record the time of day they had water contact, which is extremely important.

Reviewer #2: (No Response)

Reviewer #3: Well done!

Reviewer #4: The results are clearly outlined and address the aims (integrated consideration of hazards, exposure and vulnerability).
Some of the tables and figures could/should have acronyms described more clearly in headers and/or legends.

Response: We thank all four reviewers for their constructive comments.

We have added relevant citations to both the Introduction (lines 83-84, 119-130 in the tracked changes version) and Discussion (lines 671-675, 736- 773, 890-895 in the tracked changes version). We found the references suggested by Reviewer 1 to be very interesting pieces of research and incorporated a few where appropriate, including Madsen et al.'s 2011 paper in EcoHealth, which reports high levels of human infection despite snails occurring in very low numbers.

The reviewer makes a great point, and we strongly agree that the time of day is an important element of exposure, especially considering the chronobiology of cercarial release. Our questionnaire-based methods captured typical, regular behavior involving contact with water, rather than individual episodes of water contact. This limited our ability to account for the time of day of exposure in a meaningful or reliable way, despite its importance. When designing this study, we grappled with the trade-offs between collecting full information (frequency, duration, time of day) on the exposure of a small number of people versus collecting less complete information on a larger proportion of the population when designing this study. Obviously, we opted for the latter, measuring exposure among all school-aged children in our study population through a household survey, and incorporating additional data from the literature, interviews, and household-level survey questions to approximate some additional dimensions of water contact and exposure. This choice ultimately limited our ability to capture the time of day, out of concern that survey respondents would not be able to reliably recall the time of day for many individual episodes of water contact that may have occurred over the preceding week(s). This is a limitation of the methods we chose to use, which we have further noted in the Discussion (see lines 876-928 in the tracked changes version)

Additionally, we have added detail to captions of both figure and tables to improve clarity.

Conclusions
-Are the conclusions supported by the data presented?
-Are the limitations of analysis clearly described?
-Do the authors discuss how these data can be helpful to advance our understanding of the topic under study?
-Is public health relevance addressed?

Reviewer #1: There conclusions ignore important literature and thus they make sweeping statements that are not supported by other studies. Over all they have an excellent paper, they just need to add additional references.

Reviewer #2: (No Response)

Reviewer #3: Overall well done.

Reviewer #4: Conclusions are sounds. The discussion of weaknesses in the data at every step is commendable but detracts from the significance of their findings.
The authors clearly discuss what could be taken away from this study for efforts to disrupt the parasite lifecycle

Response: We appreciate the Reviewer 1 noting gaps in the literature we cited, both here and in the Introduction. As stated above, we have incorporated new citations in both sections of the revised manuscript. We also have consolidated methodological limitations of our study in a single section (with its own subheading) in the Discussion (see lines 876-928 in the tracked changes version)

Editorial and Data Presentation Modifications?
Use this section for editorial suggestions as well as relatively minor modifications of existing data that would enhance clarity. If the only modifications needed are minor and/or editorial, you may wish to recommend "Minor Revision" or "Accept".

Reviewer #1: (No Response)

Reviewer #2: (No Response)

Reviewer #3: Accept

Reviewer #4: See comments below

Summary and General Comments
Use this section to provide overall comments, discuss strengths/weaknesses of the study, novelty, significance, general execution and scholarship. You may also include additional comments for the author, including concerns about dual publication, research ethics, or publication ethics. If requesting major revision, please articulate the new experiments that are needed.

Reviewer #1: The title indicates it is a study of re-infection, which is misleading. In order to measure re--infection they would have to identify infected children, treat them, re-test them, and then have them exposed to water and determine re-infection

Response:
Thank you for pointing this out. The reviewer is right that our study does not meet the strict definition of re-infection, as they define it. We did not have the ability to track all exposure in all children over the course of a year. However, the data we report are a cross-section of a longitudinal study in which all children were tested in Spring 2016 to confirm infection, received double dose praziquantel treatment. They then were retested (and treated again) in Spring 2017 and Spring 2018 to estimate reinfection rates a year after treatment. Therefore, the infection data we report in our analysis reflect post-treatment infections acquired over the preceding year. Though we do not confirm that all observed infections were cleared of infection (parasitological cure) by treatment in that year, the assumed efficacy of praziquantel followed by the intensity of exposure is enough in this setting that we think that the use of the term "re-infection" is warranted. Most of the children in our study were infected at baseline and most of them remained infected in subsequent years. We have added language to clarify this point in the Methods (see lines 226-230 in the tracked changes version)

Reviewer #2: In this paper, Lund and colleagues address the interesting question of the interaction between hazard, exposure and vulnerability associated with infection and infection intensity of S. haematobium in Senegal. They found that infection presence was mostly driven hazard and vulnerability, while infection intensity is determined by exposure and hazard. I think this is an interesting paper that deserves to be published in PLoS NTDs when my comments would have been addressed.

I see two main concerns with the statistical analysis. First, they select one variable for each component (exposure, vulnerability and hazard), and then perform model selection to identify which one is the most parcimonious. Doing things like that can introduce biases. Indeed, if two variables included in their "hazard" category could explain the whole pathogen presence (or intensity), this cannot be identified because only one variable by component is selected and confronted to the others. I understand the rationale behind that, but I think it would be more robust to perform the analysis without such variable grouping.

Second, in the case where they select only one variable by group, I think including the interactions between variables could be interesting, since exposure and hazard could definitely interact between them.

On a minor note, from table 5, I don't understand the difference between model 16 and 19.

Response:
Thank you for these thoughtful comments and suggestions.

Indices within a group (i.e., exposure, hazard, or vulnerability) were highly correlated with each other. Three of the four exposure indices, for example, had correlation coefficients approaching 1. Dichotomous and categorical variables of surface water use were also positively correlated, as were snail habitat areas calculated for the peak transmission season and over the entire year. To clarify this point, we have added a correlation plot to our supplementary files that illustrates bivariate correlations between all variables included in our analysis. We believe that variables within a group represent slight variations in the measurement of the same construct within the exposure, hazard, and vulnerability framework. including multiple indices from the same group would likely introduce issues of collinearity and artificially inflate variance estimates. Additionally, specifying a "full" model that includes all possible explanatory variables is not possible with our relatively small sample size, as there are not enough degrees of freedom to support model convergence. For these reasons, we believe the approach that we've taken – including one variable from each group – is the best way to address the question we set out to answer.

Regarding interaction terms, we agree that the concepts of exposure, hazard, and vulnerability interact in determining risk for schistosome infection! In the initial stages of conducting this analysis, we fit models with interaction terms between exposure, hazard and/or vulnerability variables. When we did this, we found that the AIC nearly always increased for models with interactions terms compared to identical models without interaction terms. That is, interaction terms did not improve the fit of models in either set. Moreover, adding interaction terms complicated the visualization and interpretation of our model averaging results. With an interaction term, for example, an odds ratio representing the association between infection presence and exposure, for example, would have to be estimated across all levels of the interacting hazard or vulnerability variable. Visualizing these stratified estimates in Figure 2, which is already quite busy, would complicate the interpretation and sacrifice the clarity of our findings without generating much additional insight. Given this, we do not feel that including interaction terms adds value to our analysis.

Finally, thank you for pointing out the confusion in Table 5. These reflect two models that included indices for both exposure and hazard, but they differed in which hazard indices were included in each model. In Model 16, the hazard index was the area of snail habitat during the peak season (areaPeak), while in Model 19, the hazard index was the same measure of snail habitat area scaled by distance between the water access site and the child's household (areaPeak_d). The full details on both sets of models can be found in S4 Table (presence) and S5 table (intensity). These supplementary tables are large, and we felt it was more appropriate to report them in supplementary material than in the main text. Table 5 was meant to efficiently summarize the inclusion of exposure, hazard, and vulnerability as variables in the best-fitting models in each set. We reference the supplementary tables in Table 5 to direct the interested reader to the full details of each set of models.

Reviewer #3: (No Response)

Reviewer #4: This research article highlights the importance of environmental hazards, exposure and vulnerability on the risks of urogenital schistosomiasis affecting school-aged children in a region of northern Senegal. Their findings extend work that has primarily focused on the demographic features of a population that affect the risk of infection. Consideration for each component of risk seems highly logical but rarely reported in an integrated way as they have reported here. This is a key, novel feature of this article (in my opinion). Discussion highlights several weaknesses in the approach used herein. Although this has limited the power of the analyses (as mentioned in the discussion), it also highlights that there is a lot more work to do in planning future epidemiological work. Overall, the methods look quite comprehensive. I was unable to review the DRYAD information using the url provided for dryad.s7h44j15p. Is it publicly available? You mention three studies
that were undertaken to obtain the data used herein. Some abbreviation of previously published methods may be warranted here. Please make sure they are cited, similar to the PNAS manuscript (reference 18). This will make it clear what methods are unique to this manuscript.
I have some other suggestions that I think are worth considering.

The introduction suggests a broad range of hazards should be considered, including floods and extreme weather events. Despite this being a focus in the introduction, it wasn't a focus on the analysis and no data reported on extreme/catastrophic events in the region studied during the time of sampling. For this reason, I suggest simplifying the introduction to focus on the risks identified in a more usual seasonal cycle in the region (as presented in the results and discussed).

Narrative in the introduction can be confusing. I would consider starting with schistosomiasis or significantly reduce the background at the start of the introduction. I don't think an extended justification for looking at risk (exposure, hazard, and vulnerability) is required (start of the introduction). Most readers would be aware of this topic. Brief explanation would be better with an introduction that is focussed on the subject (schistosomiasis).

Lines 144 to 147. Please make it clear in the introduction why demographic features were included as control here. It is in the methods, results and discussion, but the approach could be better presented in the last paragraph of the discussion.

Line 222 to 223. Something missing in this sentence
Lines 529 to 532.Please include references to support these statements.
Line 618 and Line 648. Manuscript is well written but please check for grammatical errors throughout

Discussion. Would it be better to incorporate the limitations of this study into one section and discuss how this can be resolved in the future. It feels repetitive at times and detracts from the significant results obtained in this study.

Response:

Thank you for the praise and constructive comments.

We apologize for the confusion about the Dryad dataset. The data are privately available for peer review. We meant to share the temporary "Private for Peer Review" link but mistakenly provided the public doi link (which is not yet active) in our initial submission. We have included the correct link below. Reviewers should be able to find the data set (aggregated to village level to conform with human subjects protections for individual-level infection data) at this temporary link for peer review purposes: https://datadryad.org/stash/share/7i3cLq46DaaD2eGw7FTslGP8lbBL1VStyWIxMi9WL0Q

We cited all studies from which we drew data or methods. Body surface area exposed was measured through interviews employing a diagram for measuring burn size (reference 44) and calculations for body surface area (reference 45). The duration of exposure for six common water contact activities was drawn from data reported in reference 39. The index of socio-economic status was calculated based on methods described in references 46 and 47. Aside from these and the hazard metrics developed from data also reported in reference 33, the data and the methods used to collect them are unique to this manuscript and have not been published elsewhere.

In the Introduction, we don't mean to imply that flooding and extreme weather are immediately relevant to schistosomiasis risk, only to highlight a related field that readily employs the "exposure-hazard-and vulnerability" framework. We think this framing lends itself well to the risk of environmentally-mediated infectious diseases, including schistosomiasis, but found very few papers on the subject that use it. We feel it's important to remain in conversation with the distinct but related fields that use the constructs of exposure, hazard, and vulnerability to understand risk of adverse events. For this reason, we have retained the justification at the beginning of the Introduction.

We have added language and citations to the end of the Introduction regarding the inclusion of demographic characteristics as control variables (see lines 172-173 in the tracked changes version)

We have corrected the errors identified the reviewer identified in lines 222-223, 618 and 648 of the original version of the manuscript. Thank you for spotting these! We have also supported the statements in lines 529-532 (now line 670-675 with tracked changes) with references and additional text.

Finally, as stated previously, we have consolidated methodological limitations of our study in a single section (with its own subheading) in the Discussion (see lines 876-928 in the tracked changes version).

Click here to access/download
Revised Article with Changes Highlighted
Lund_etal_EHV_PNTDs_revised_tracked.docx
