# Supplementary material for: Exposure, hazard, and vulnerability all contribute to Schistosoma haematobium re-infection in northern Senegal
Source: PLoS Negl Trop Dis. 2021 Oct 5;15(10):e0009806. doi: 10.1371/journal.pntd.0009806 (PMC8525765; doi:10.1371/journal.pntd.0009806)
Supplement: Attachment — Submitted filename: Lund_EHV_responseLetter_2.docx [file pntd.0009806.s012.docx]

Dr. Poppy H. Lamberton

Deputy Editor

Dr. Anna Last

Associate Editor

PLOS Neglected Tropical Diseases

Dear Dr. Lamberton and Dr. Last:

Thank you for considering our revised manuscript (PNTD-D-20-02100R1) “Exposure, hazard, and vulnerability all contribute to *Schistosoma haematobium* re-infection in northern Senegal” for publication in PLOS Neglected Tropical Diseases. We thank you and the three reviewers for your attention to detail in reviewing the revised version of the manuscript. We have made the requested minor changes and carefully re-read the manuscript in its entirety to double-check for typos and grammatical errors.

Below, you will find our line-by-line responses, with two versions of the manuscript (one clean and one with tracked changes) included with the submission. We hope that, with these changes, you will find this newly revised version of the manuscript to be acceptable for publication.

Once again, we reiterate that this manuscript is original and is not being considered for publication elsewhere. We have no conflicts of interest to disclose.

Thank you for your consideration.

Sincerely,

Andrea Lund, PhD MPH

On behalf of all co-authors

**Response to reviews**

Reviewer's Responses to Questions

Key Review Criteria Required for Acceptance?
As you describe the new analyses required for acceptance, please consider the following:

Methods
-Are the objectives of the study clearly articulated with a clear testable hypothesis stated?
-Is the study design appropriate to address the stated objectives?
-Is the population clearly described and appropriate for the hypothesis being tested?
-Is the sample size sufficient to ensure adequate power to address the hypothesis being tested?
-Were correct statistical analysis used to support conclusions?
-Are there concerns about ethical or regulatory requirements being met?

Reviewer #1: yes

Reviewer #2: (No Response)

Reviewer #3: As suggested in reviews for the 1st submission, the methods are overall conceived and designed. Minor issues were appropriately addressed in the revision.

**Response: Thank you. We appreciate these positive comments.**

--------------------

Result
-Does the analysis presented match the analysis plan?
-Are the results clearly and completely presented?
-Are the figures (Tables, Images) of sufficient quality for clarity?

Reviewer #1: yes

Reviewer #2: (No Response)

Reviewer #3: Well done!

**Response: Thank you very much!**

--------------------

Conclusions
-Are the conclusions supported by the data presented?
-Are the limitations of analysis clearly described?
-Do the authors discuss how these data can be helpful to advance our understanding of the topic under study?
-Is public health relevance addressed?

Reviewer #1: yes

Reviewer #2: (No Response)

Reviewer #3: Yes.

**Response: Thank you!**

--------------------

Editorial and Data Presentation Modifications?
Use  this section for editorial suggestions as well as relatively minor modifications of existing data that would enhance clarity. If the only modifications needed are minor and/or editorial, you may wish to recommend “Minor Revision” or “Accept”. 

Reviewer #1: There are many places in the ms that nouns are used as adjectives.  I marked many of these, but the authors should do a global search.  One cannot use scientific names as adjectives! The introduction should be expanded slightly to indicate that there are many types of schistosomes that infect other mammals and birds. Should clarify that the paper deals with urinary schistosomes of humans, but there are also schistosomes that infect the bowels of humans.

Reviewer #2: (No Response)

Reviewer #3: (No Response)

**Response: Thanks to Reviewer 1 for bringing these details to our attention. We have clarified in line 103 of the Introduction that this paper deals primarily with human schistosomes. In lines 135-136, we state that the study focuses on urogenital schistosomiasis and have added in parentheses that urogenital schistosomiasis is caused by *S. haematobium.***

**With the various instances where we use nouns as adjectives, we find it difficult to take Reviewer 1’s suggestion and maintain the clarity of our methods and results. We feel that repeating “models of the presence/intensity of infection with S. haematobium” rather than “models of S. haematobium infection presence/intensity” will make the paper harder to read and understand. We have taken this suggestion in some places, but have retained the more succinct, if slightly incorrect, usage in others for the sake of clarity.**

--------------------

Summary and General Comments
Use  this section to provide overall comments, discuss strengths/weaknesses of the study, novelty, significance, general execution and scholarship. You may also include additional comments for the author, including concerns about dual publication, research ethics, or publication ethics. If requesting major revision, please articulate the new experiments that are needed.

Reviewer #1: This is an excellent study and should be published.

Reviewer #2: I am happy with author's replies and therefore recommend acceptance

Reviewer #3: The authors have done a good job in addressing the reviewer's comments.

**Response: We thank all three reviewers for their positive and constructive comments in this and the previous round of review.**

--------------------

PLOS authors have the option to publish the peer review history of their article. If published, this will include your full peer review and any attached files.

If you choose “no”, your identity will remain anonymous but your review may still be made public.

Do you want your identity to be public for this peer review?</b> For information about this choice, including consent withdrawal, please see our Privacy Policy.

Reviewer #1: Yes: Jay R. Stauffer, Jr.

Reviewer #2: Yes: Benjamin Roche

Reviewer #3: No

--------------------

Editor Poppy Lamberton comments:

In line 61 in the authors summary please change from ‘is acquired, where increasing numbers of worms lead to increasing severity of disease.’ To ‘is acquired. Increasing numbers of worms is known to be positively associated with increasing severity of disease.’ As you don’t actually measure the severity of disease in your study.

**Response: Done.**

Intro line 71: respond to, or recover from,…. Please add these two commas.

**Response: Done.**

Line 126 change to hotspots and line 727, as for most of the references for it.

**Response: Done.**

Methods: line 190, please clarify that you mean urine on two days at each time point, or a single urine sample at two time points? I assume the former, but good to clarify.

**Response: Done.**

Line 235: Please change schistosome infections to Schistosoma haematobium as that was what you tested for.

**Response: Done.**

Lines 237: please clarify at what level you mean the medians, a cross the whole cohorts at each time point? Or across multiple samples from each person at each timepoint? And you can justify the median also as I assume the data were not normally distributed? Likely negative binomial distribution?

**Response: Done.**

Results

Line 432: best fit: I think this is an example of where you use a noun rather than the verb. Maybe rephrase to was the best fit for the s.h presence data.

**Response: Done.**

Discussion:

Line 607: found ‘the’ area of snail habitat to be a…: please add either a ‘the’ or inverted commas: found ‘area of snail habitat’ to be a …

**Response: Done**

Line 616: change fit to fitted.

**Response:**

Line 617: found proximity to water access points: please write either found the proximity to water access points…. Or found ‘proximity to water access points’  as above

**Response: Done.**

Line 623: fit to fitted

**Response: Done.**

Line 662: represented access to, and use of, water and sanitation

**Response: Done.**

Line 666: in ‘the’ highest and lowest SES quintiles

**Response: Done.**

Line 716: household level, most contribute…. Please change to household level, contribute most…. And for line 718

**Response: Done.**

Line 718: change parasites to S. haematobium parasites

**Response: Done. Thank you for pointing out these details! We very much appreciate the close read.**
